# Waveguide-integrated 2D-material infrared photodetectors: fundamentals, advances, and insights

**DOI:** 10.1038/s41377-026-02412-4

**Published:** 2026-11-12

**Authors:** Jiachen Wang, Zezhou Lin, Yi Ren, Yongji Wang, Ze Feng, Huashun Wen, Kunpeng Zhai, Jiazheng Sun, Sha Zhu, Ning Hua Zhu

**Affiliations:** 1 Institute of Intelligent Photonics, Nankai University, Tianjin, 300071 China; 2 Department of Applied Physics, The Hong Kong Polytechnic University, Hong Kong, China; 3 Department of Chemistry, City University of Hong Kong, Kowloon, Hong Kong, China

**Keywords:** Optoelectronic devices and components, Optical sensors

## Abstract

Photodetectors that integrate two-dimensional (2D) materials with optical waveguides are considered an ideal approach to enhance infrared detection capability and on-chip integration capability and have become a focal point of recent research. These devices benefit not only from the intrinsic optoelectronic properties and flexible hetero-integration of 2D materials but also from a range of engineering strategies that enable precise control over their electronic and optical characteristics. In addition, by combining different types of waveguide structures, the optical field distribution can be effectively tailored, further strengthening light-matter interactions and facilitating the realization of compact, high-performance infrared photodetectors with versatile strategies. This review systematically summarizes the latest progress in waveguide-integrated 2D-material infrared photodetectors, organized primarily by the type of waveguide material and further categorized according to the 2D materials employed. It discusses the advantages of various waveguide structures and 2D materials, the working mechanisms, performance parameters, common device architectures, and many key approaches to improving photodetector performance (e.g., waveguide structure optimization, electrode material and spacing design, gate electrostatic doping, substrate engineering, strain modulation, twist angle control of 2D materials, heterojunction band alignment, etc). Furthermore, a comparative analysis with conventional commercial photodetectors is provided, demonstrating that waveguide-integrated 2D-material infrared photodetectors have already achieved comparable performance in many applications, while certain challenges remain. Finally, the review outlines the key obstacles facing practical implementation and offers perspectives on future research directions.

## Abstract (web summary)

This review examines waveguide-integrated two-dimensional-material infrared photodetectors, focusing on waveguide platforms, material integration, device mechanisms, performance enhancement strategies, and remaining challenges for practical on-chip detection.

## Graphical abstract

**Figure:**
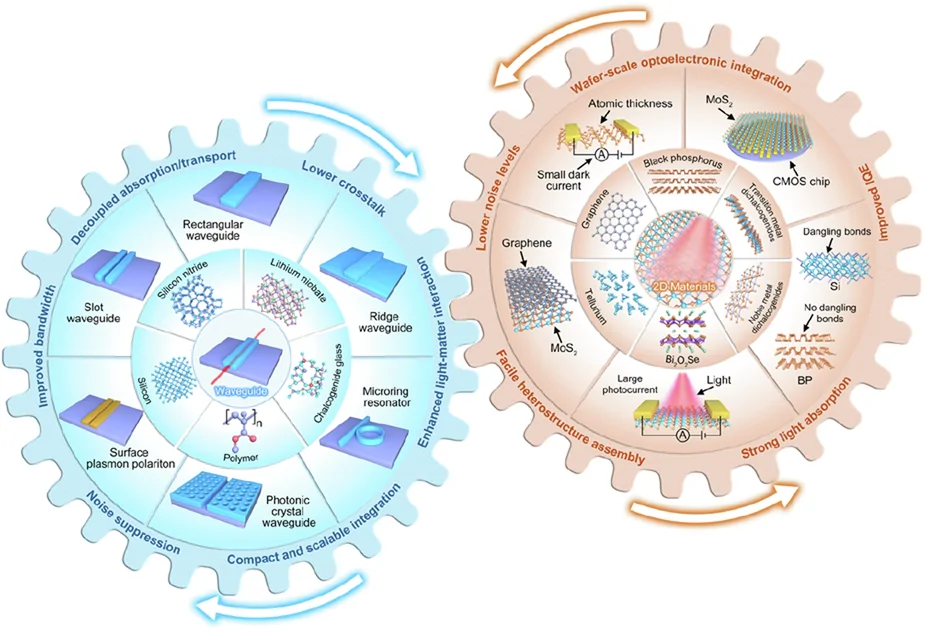

## Introduction

Owing to the accelerating progress in digital technologies, the world is witnessing exponential data growth^1,2^. Traditional electronic chips face significant challenges in enhancing performance and controlling power consumption, making it difficult to satisfy the demands of future high-speed computing, large-capacity information transmission, and low power consumption^3^. In this context, photonic integrated circuits (PICs) have become a revolutionary technology. Utilizing photons as information carriers, PICs offer remarkable advantages such as high transmission speed, broad bandwidth, strong resistance to electromagnetic interference, and low energy consumption^4,5^. These features position PICs as a crucial force in surmounting the constraints of conventional electronic chips and propelling the advancement of next-generation information technologies. Silicon has long been the cornerstone of PICs, owing to its mature fabrication processes, cost-effectiveness, strong optical confinement, and favorable compatibility with complementary metal-oxide-semiconductor (CMOS) technology, which has the capability to entirely reshape the industries for information transmission and telecommunications^6^. However, in recent years, the pursuit of higher performance, broadband operation, and multifunctional integration has driven the incorporation of a variety of emerging materials into photonic platforms^7,8^. For example, silicon nitride has been extensively employed in nonlinear optics and optical frequency comb generation due to its ultra-low loss and broad transparency window^9^; lithium niobate, with its outstanding electro-optic properties, is well suited for high-speed modulation and optical signal processing^10^; chalcogenide glasses offer excellent transparency in the mid-infrared region and possess high nonlinear coefficients, making them ideal for mid-infrared integrated devices^11^; and polymer materials, characterized by their mechanical flexibility, facile manufacturability, and economic fabrication are promising candidates for wearable optoelectronics and flexible photonic systems^12^.

Moreover, a complete PIC typically comprises many photonic elements, including low-loss waveguides, light sources, modulators, infrared photodetectors, and so on^13^. Among them, infrared photodetectors play a pivotal role in converting infrared optical signals between 0.8 and 30 μm in wavelength into electrical ones, making them indispensable in applications such as optical communication^14^, sensing^15^, and computing^16^. The performance of photodetectors critically influences the overall efficiency and reliability of PICs. To date, commercial infrared photodetectors have largely relied on traditional bulk materials such as silicon^17^. While these materials offer mature performance, they also present inherent limitations. For instance, silicon, with its indirect bandgap of ~1.1 eV, is ineffective for detection beyond the near-infrared range^18^; additionally, its high carrier concentration leads to increased leakage current and higher power consumption. In addition, the bulky form factor of conventional photodetectors often hinders seamless integration with photonic platforms, necessitating heterogeneous integration of substitute materials such as III–V semiconductors^19^, graphene^20^, transition metal dichalcogenides (TMDs)^21^, and other emerging narrow-bandgap two-dimensional (2D) materials to satisfy the increasing need for miniaturization, high performance, and on-chip compatibility^22^. However, despite the feasibility of epitaxially growing III–V materials on silicon substrates, the lattice and thermal mismatches frequently introduce interfacial defects that degrade optoelectronic properties^23^.

In contrast, 2D materials have emerged as attractive options for photonic integration, attributed to their strong light-matter interactions, broad and adjustable bandgaps (0–2.5 eV) enabling absorption over a wide spectrum, atomically thin surfaces free of dangling bonds, and superior optoelectronic properties^24^. In particular, the absence of dangling bonds not only facilitates excellent interfacial passivation, mitigating performance degradation from surface states, but also enables seamless integration with a variety of materials, enabling versatile and tunable heterojunction architectures^25–27^. Furthermore, the evolution of chemical vapor deposition (CVD) techniques has enabled the scalable and superior synthesis of 2D materials, which can be transferred at the wafer scale onto basically any other substrate and hence allows seamless integration with traditional CMOS technology and PICs^28^. Thus, researchers have employed various 2D materials and engineered heterostructures to develop advanced infrared photodetectors demonstrating performance metrics such as response speed, detection sensitivity, and operational bandwidth that equal or even exceed those of conventional commercial photodetectors^29^.

On-chip infrared photodetectors utilizing 2D materials typically fall into two categories: surface-illuminated and waveguide-integrated configurations. Surface-illuminated infrared photodetectors receive more research focus on account of their structural simplicity and enhanced performance^30–32^. Nevertheless, such devices have some shortcomings, such as intrinsically limited interaction paths and hence weak light absorption in the vertical dimension due to the atomic-scale thickness of 2D materials. Besides, such a configuration is also hindered by large footprints and weak optical confinement, reducing its photonic integration potential^33^. To address these challenges, waveguide-integrated infrared photodetectors offer a promising solution. By coupling 2D materials with optical waveguides, the light-material interaction length is significantly increased while maintaining the inherent advantages of 2D materials (e.g., low noise and high carrier mobility), thereby enhancing light absorption^34,35^. Moreover, the waveguide-integrated configuration is compatible with CMOS fabrication processes and features a compact device size, facilitating highly efficient optical integration^36^.

**Fig. 1:**
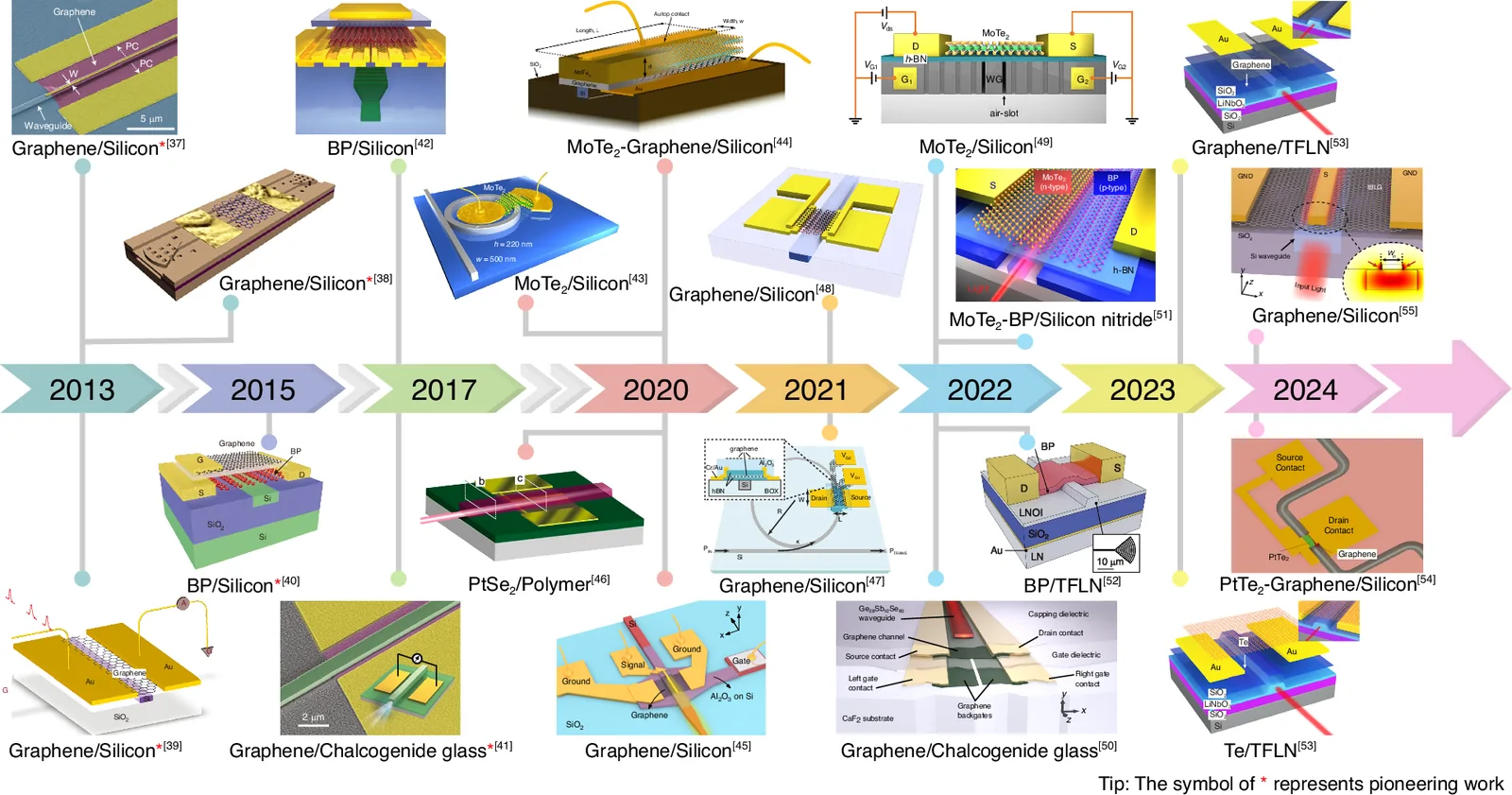
Overview of waveguide-integrated 2D-material infrared photodetectors. Developmental lineage of waveguide-integrated 2D-material infrared photodetectors classified by year: 2013^37–39^, 2015^40^, 2017^41,42^, 2020^43–46^, 2021^47,48^, 2022^49–52^, 2023^53^, and 2024^54,55^. Reproduced from ref. ^37^ with permission of Springer Nature. Reproduced from ref. ^38^ with permission of Springer Nature. Reproduced from ref. ^39^ with permission of Springer Nature. Reproduced from ref. ^40^ with permission of Springer Nature. Reproduced from ref. ^41^ with permission of Springer Nature. Reproduced from ref. ^42^ with permission of Copyright © 2017 American Chemical Society. Reproduced from ref. ^43^ with permission of Springer Nature. Reproduced from ref. ^44^ with permission of Springer Nature. Reproduced from ref. ^45^ under (CC BY 4.0). Reproduced from ref. ^46^ with permission of Copyright © 2020 American Chemical Society. Reproduced from ref. ^47^ under (CC BY 4.0). Reproduced from ref. ^48^ under (CC BY 4.0). Reproduced from ref. ^49^ with permission of Copyright © 2022 American Chemical Society. Reproduced from ref. ^50^ under (CC BY 4.0). Reproduced from ref. ^51^ under (CC BY 4.0). Reproduced from ref. ^52^ under (CC BY 4.0). Reproduced from ref. ^53^ under (CC BY 4.0). Reproduced from ref. ^54^ with permission of Copyright © 2024 American Chemical Society. Reproduced from ref. ^55^ under (CC BY 4.0)

Since researchers first integrated graphene with silicon waveguides to fabricate photodetectors in 2013, numerous on-chip waveguide structures have been successfully combined with various 2D materials, resulting in infrared photodetectors with significantly enhanced light–matter interactions and multifunctional capabilities, as illustrated in Fig. 1^37–55^. During this period, several high-quality review articles have been published^34–36,56–58^, addressing waveguide devices, 2D materials, and infrared photodetectors from different perspectives. In contrast to these existing reviews, the present work focuses specifically on the synergistic enhancement mechanisms arising from the integration of on-chip waveguide structures with 2D materials, and further incorporates comparisons with commercial photodetectors as well as discussions on commercialization-related challenges, thereby providing an application-oriented perspective on the future development of waveguide-integrated 2D-material infrared photodetectors. Building on these considerations, this review summarizes recent progress in waveguide-integrated 2D-material infrared photodetectors. We first explore the individual advantages of waveguides and 2D materials for infrared photodetectors, and then briefly introduce common waveguide structures and materials, along with types of 2D materials. Subsequently, the fundamentals of waveguide-integrated 2D-material infrared photodetectors are systematically and thoroughly discussed, including photon-type and thermal-type working mechanisms, three different on-chip infrared detector structures, and performance parameters. Next, we categorize and summarize the research advancements in these devices based on the waveguide materials employed. Particularly, multiple approaches for enhancing the performance of waveguide-integrated infrared photodetectors are comprehensively elucidated, such as waveguide structure optimization, electrode material and spacing design, gate electrostatic doping, substrate engineering, strain modulation, twist-angle control of 2D materials, heterojunction band alignment, etc. Finally, we compare the different performance characteristics of waveguide-integrated infrared devices and commercial detectors and conclude the review by proposing possible solutions to address current challenges in infrared photodetector development.

## Advantages and categories of waveguides for infrared photodetectors

### Advantages of waveguides for infrared photodetectors

**Fig. 2:**
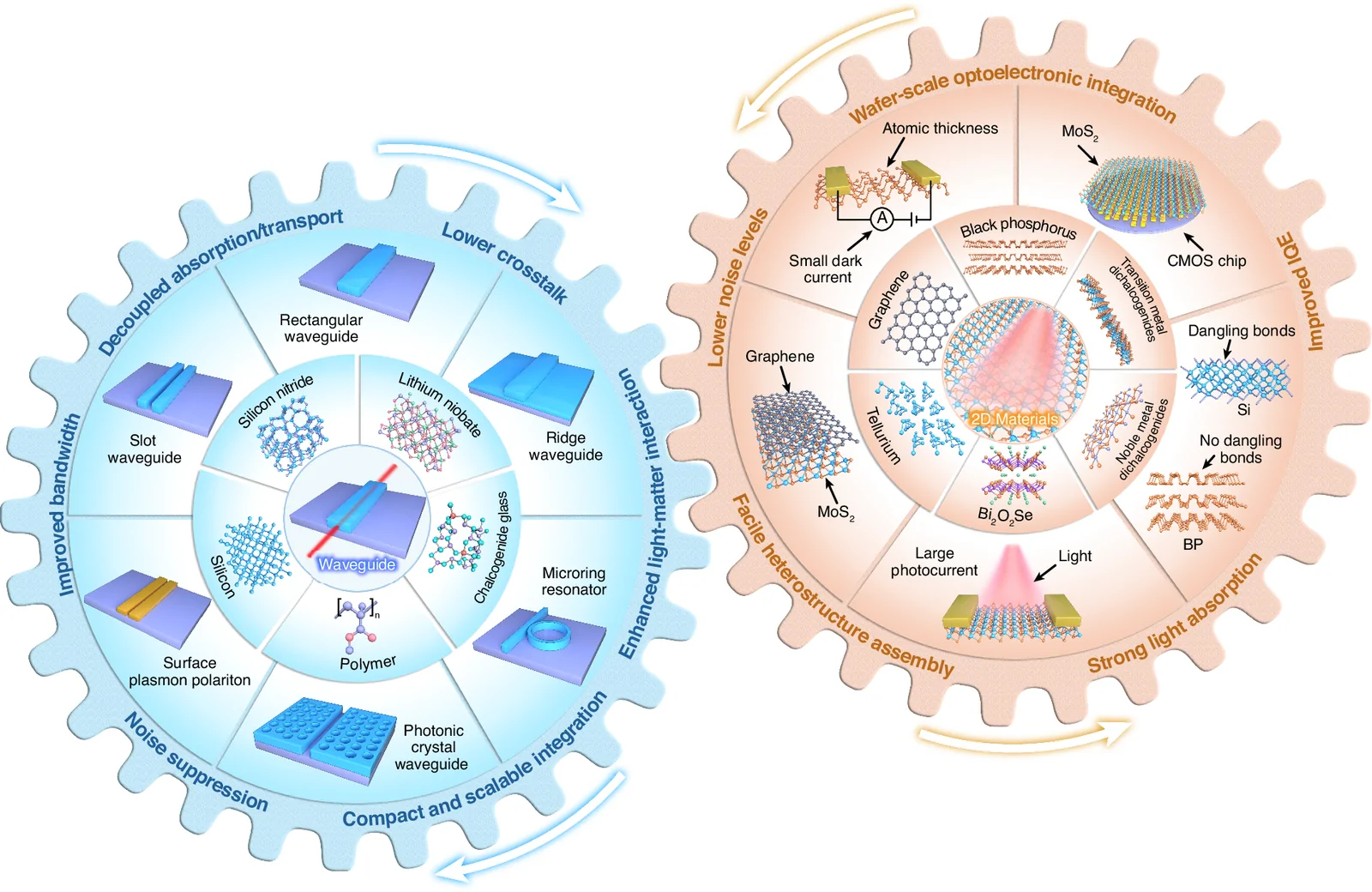
Advantages of diverse waveguides and 2D materials in photodetectors. The left gear illustrates various waveguide materials and structures utilized in photodetectors, with the outer ring highlighting the specific advantages associated with each waveguide type. The right gear depicts various 2D materials used in photodetectors, while the outer ring summarizes their respective benefits in device applications

As the fundamental component of PICs, optical waveguides can confine and guide light efficiently within or adjacent to the waveguide’s surface, enabling strong light-matter interactions, which makes them an ideal platform for integrating 2D materials to fabricate high-performance infrared photodetectors^36,57,59^. Compared to conventional surface-illuminated photodetectors, which rely on direct free-space illuminance, waveguide-integrated photodetectors utilize evanescent-field coupling or lateral absorption mechanisms and exhibit much more significant advantages, which are summarized as follows (Fig. 2). 1) Enhanced light–matter interaction and higher detection efficiency: Waveguide-integrated photodetectors utilize efficient light confinement in the waveguide structure to extend the interaction path between guided light and the absorbing material, thereby substantially promoting absorption capability, especially for infrared wavelengths with inherently weak absorption properties^36,60^. 2) Improved bandwidth and high-speed operation: Waveguide-integrated photodetectors typically display smaller resistor-capacitor (RC) time constants because of their improved impedance matching with electronic circuits and smaller device capacitance^61^. Moreover, on account of the guided transmission of light, waveguide-integrated photodetectors can significantly reduce transit-time constraints by optimizing charge transfer dynamics inside the absorption zone^62^. Consequently, they can realize greater bandwidth in comparison with surface-illuminated photodetectors, which have charge diffusion bottlenecks when exposed to broad spatial illumination. 3) Lower crosstalk: In tightly integrated photonic circuits, optical and electrical crosstalk can result in deteriorated signal integrity. Due to direct irradiation of spatial light, surface-illuminated photodetectors are often subject to undesired reflections, scattering, and interference from other adjacent optical signals^58^. However, waveguide-integrated photodetectors can control optical wave propagation in the waveguide and thereby effectively minimize the influence of external stray light. 4) Noise suppression and higher signal-to-noise ratio: The range of noise that affects the signal-to-noise ratio (e.g., shot noise, Johnson noise, and generation-recombination noise) is usually proportional to the effective detection area of the photodetector^63^. In waveguide-integrated photodetectors, when light is input through a waveguide (size factor is *n*), the active area of the photodetector can be decreased by about a factor of *n*^2^ without affecting light absorption^64^, and the noise will also be reduced linearly in the same proportion, which is crucial for reducing the high noise of narrow bandgap semiconductors in infrared photodetectors. 5) Removal of the mutual constraints between light absorption efficiency and carrier collection efficiency: In general, high light absorption efficiency depends on an adequately extended light absorption length to absorb incident photons, while effective charge collection needs a shorter carrier transmission distance, resulting in mutual constraints between these two factors^57^. For traditional surface-illuminated photodetectors, the light transmission path and the carrier collection path are usually overlapped, while the two paths of waveguide-integrated photodetectors are perpendicular to each other, thus simultaneously achieving high light absorption efficiency and carrier collection efficiency. 6) Compact and scalable integration with photonic circuits: Surface-illuminated photodetectors require free-space optics and precise alignment, which increases system complexity and footprint^35^. In contrast, waveguide-integrated photodetectors eliminate the need for bulk optical components, leading to reduced device size, improved integration density, and better compatibility with silicon photonics and other integrated platforms^36^. This makes them ideal for large-scale optical interconnects and infrared sensor arrays.

### Categories of waveguides utilized for infrared photodetectors

As mentioned above, optical waveguides offer effective optical transmission routes while facilitating the restriction and amplification of the light field, unlocking the considerable promise to improve the efficiency of conventional infrared photodetectors. Up to now, many different kinds of optical waveguide structures and materials have been utilized and investigated in waveguide-integrated photodetectors. Classified by the waveguide structure, they are usually composed of rectangular waveguides, ridge waveguides, slot waveguides, surface plasmon polariton waveguides, photonic crystal waveguides, and microring resonators. While based on the waveguide material used, they encompass silicon waveguides, silicon nitride waveguides, thin film lithium niobate (TFLN) waveguides, chalcogenide glass waveguides, and polymer waveguides, as shown in Fig. 2.

When categorized by the waveguide structure, rectangular and ridge waveguides are the most widely used waveguide designs for integrating infrared photodetectors. Rectangular waveguides, usually rectangular in shape, are composed of core and cover layers with varying refractive indices and can efficiently direct and manipulate nanoscale optical signals^65^. In contrast, a ridge waveguide is formed by locally retaining a portion of the thin-film layer on the waveguide surface to create a ridge, where the refractive-index contrast between the ridge and the underlying residual film confines the optical field^38,53,66^. Each type of waveguide has its advantages and disadvantages: rectangular waveguides offer stronger mode confinement in both the lateral and vertical directions due to their high refractive-index contrast, but they require deep etching processes and are extremely sensitive to sidewall roughness—nanometer-scale roughness can lead to propagation losses exceeding 1 dB cm^−1^^67^. Ridge waveguides, by contrast, tolerate fabrication variations more readily, since only shallow etching is needed and sidewall roughness has a relatively limited impact on loss; however, to avoid mode leakage, ridge waveguides generally require larger bending radii^68^, resulting in a lower integration density compared to rectangular waveguides. Overall, both rectangular and ridge waveguides can be manufactured through conventional nanomanufacturing methods like electron beam lithography, etching, and photolithography, which renders them appropriate for large-scale fabrication and efficient compact integration with 2D materials and many other optical components^69,70^.

In contrast to the previous two waveguide structures, which guide and propagate light through materials with high refractive index, slot waveguides uniquely restrict light to a slot filled with low refractive index substances, such as air and other similar alternatives^71,72^. They can also provide significantly enhanced light-field restriction within the middle slot area compared with rectangular and ridge waveguides, which dramatically increases the light-matter interaction between transmitted light and integrated 2D materials and thus enables high-performance infrared photodetectors^71,73^. Surface plasmon polaritons are phenomena that occur at the boundary between metallic and insulating materials, characterized by joint electron oscillations triggered by incoming optical waves^74^. The subwavelength characteristics of surface plasmon polaritons enable them to concentrate nanoscale light energy and hence substantially improve light–matter interactions^37,45,75^. Surface plasmon polariton waveguides exploit the characteristics of surface plasmon polaritons to effectively restrict and transmit light at the boundary between metallic and insulating materials^76^. These waveguides are generally constructed from a combination of metals and insulators, with Au and Ag frequently selected as the metallic components because of their superior electrical conductivity^42,77,78^. Surface plasmon polariton waveguides also exhibit a variety of distinctive optical characteristics, for instance, partial improvement of electromagnetic fields and the occurrence of surface plasmon resonance^77,79^. These attributes render them suitable for various applications such as sensing technology and integrated photonics.

Photonic crystals refer to optical components made from materials that possess periodically varying refractive indices or insulation constants^80–82^. By adjusting the configuration of photonic crystals, one can establish a forbidden band that prevents the transmission of optical waves at certain frequencies^83,84^. Photonic crystal waveguides are fabricated by incorporating defects or partial features within the photonic crystal, thereby constructing directed pathways for propagating light^80,85^. Within these waveguides, the transmission of optical signals is restricted by the forbidden band structure, which enables efficient optical signal propagation over extended distances with significantly reduced loss. In addition, the periodically changing structure of photonic crystal waveguides can result in interference and scattering during the transmission of optical signals and hence decrease their transmission speed, thereby enhancing light–matter interaction and boosting photoelectric conversion efficiency^85,86^.

Microring resonators consist of a ring-shaped high-index waveguide, where optical waves travel in a circular route and experience several reflections, leading to the overlapping of light from various trajectories^47,87,88^. When the superposed optical signals meet the criteria for coherence, they produce interference phenomena that are either intensified or diminished^87,89,90^. As a result, certain wavelengths of optical waves are intensified (known as resonant wavelengths), while minimal optical waves pass through at other wavelengths, thus resulting in optical resonance^87^. When the wavelength of optical signals aligns with the resonant wavelength of microring resonators, the optical signals can concentrate energy within the cavity, resulting in the formation of an intensely concentrated optical field at the peak of resonance. Due to their diminutive footprint, superior quality factor, and adjustable properties, microring resonators are particularly advantageous for developing advanced, integrated, and high-performance photodetectors^47,89^.

When classified by the waveguide material, silicon waveguides and silicon nitride waveguides are more commonly utilized to fabricate on-chip infrared photodetectors^37–40,43–45,47,48,51,55,91^. Silicon waveguides, possessing a large refractive index of around 3.4 and small transmission attenuation under 0.5 dB cm^−1^, can restrict and direct optical waves to propagate over long distances within the waveguide with near-lossless transmission^37,39,67,70,92^. Moreover, silicon functions as a crucial fundamental element for CMOS technology and hence is compatible and integrable with contemporary silicon PICs. Although substantial progress has been made in silicon waveguides, this platform still suffers from intrinsic drawbacks, including nonlinear losses at high optical power levels due to two-photon absorption. Alternatively, silicon nitride waveguides display small propagation attenuation as low as 0.008 dB cm^−1^, a refractive index of 1.98, as well as low relative permittivity, which is beneficial for the miniaturization of optical components and the reduction of device capacitance (conducive to the fabrication of high-speed photonic devices)^51,91,93–95^. Besides, the high Kerr nonlinearity of silicon nitride waveguides renders them particularly suitable for exploring nonlinear photonics. Moreover, silicon nitride waveguides can also show extremely low optical transmission loss in a bent waveguide structure, only 0.04 dB/90°^93,94^.

TFLN waveguides are key components in integrated photonics, offering excellent electro-optic, nonlinear, and optical properties, such as small propagation attenuation, large refractive indices, pronounced nonlinearity, thermal and chemical stability, and extensive transparent window (0.4–5 μm)^96–99^. It is precisely these properties that enable TFLN waveguides to serve as a stable and reliable optical platform for heterogeneous integration, thereby enhancing synergistic performance with other materials. Integrating them with 2D materials can effectively realize high-performance infrared photodetectors^52,53,100,101^, for example, TFLN waveguide-integrated photodetectors based on tellurium demonstrate large responsivities of 7 A W^−1^ under 1550 nm illumination while also enabling GHz frequency responses^53^. Photonic chalcogenide glass, composed of sulfur, selenium, or tellurium, also possesses many unique optical characteristics, for instance, broad transparency from visible to infrared, strong Kerr nonlinearity, as well as large and consecutively tunable refractive indices between 2 and 3.5^41,50,102,103^. They can be directly patterned onto diverse 2D materials to function as waveguides without harming the structural integrity or optoelectronic characteristics of the 2D material substrates^41^. Due to the large electro-optic coefficient, ease of fabrication, and strong compatibility with current manufacturing procedures, polymer waveguides have also been extensively studied^46,104–107^, for example, various polymer waveguide materials are suitable for chip-level light interconnects on silicon substrates and have achieved notable progress in applications like infrared photodetectors and optical fibers^46,106,108^. For clarity, the key advantages, limitations, and typical application scenarios of these waveguide structures and material platforms are summarized in Table 1.

**Table 1:** Advantages, disadvantages, and application scenarios of various waveguide structures and material platforms

	Category	Advantage	Disadvantage	Application scenario
**Structure**	Rectangular	Strong confinement	Highly sensitive to sidewall roughness	High-density integration
	Ridge	Good fabrication tolerance, low loss	Larger bend radius, weaker confinement	Low-loss routing
	Slot	Strongly enhanced field	Tight tolerances, narrow gap	Ultra-compact, high-responsivity detectors
	SPP	Subwavelength confinement	High metal absorption loss	Plasmonic sensing
	PhC	Slow light, low loss	Complex fabrication	Compact narrowband detectors
	MRR	Compact footprint, resonant enhancement	Detuning-sensitive	Wavelength-selective detectors
**Material**	Silicon	CMOS-compatible	Strong nonlinear absorption	High-density integration
	Silicon nitride	Low loss	Lower index contrast	Low-noise routing for detector circuits
	TFLN	Wide transparency	High cost	EO modulation-detection platform
	Chalcogenide glass	MIR transparent	Lower mechanical robustness	MIR integrated detectors
	Polymer	Flexible substrate	Limited thermal stability	Wearable and flexible sensors

*SPP* surface plasmon polariton, *PhC* photonic crystal, *MRR* microring resonator, *TFLN* thin film lithium niobate, *CMOS* complementary metal-oxide-semiconductor, *EO* electro-optic, *MIR* mid-infrared

## Advantages and categories of layered 2D materials for infrared photodetectors

### Advantages of layered 2D materials for infrared photodetectors

In contrast to traditional bulk materials (e.g., Ge and Hg_*x*_Cd_1−*x*_Te), 2D materials have several superior properties due to their unique atomic structure, which can be summed up as follows (Fig. 2). 1) Although only a few atoms thick, 2D materials interact intensely with light, allowing photon energies near their bandgap to efficiently produce electron–hole pairs and achieve outstanding optical absorption^109,110^. For instance, single layers of graphene and molybdenum disulfide can effectively absorb approximately 2.3% and 10%, respectively, of vertically incoming light even though their thicknesses are less than 1 nm^111–115^. Relatively thick BP flakes can realize approximately 40% light absorption under polarized irradiation^116,117^, while 300 nm molybdenum disulfide flakes can absorb up to 95% of incident optical signals^109^. 2) Interlayer van der Waals interactions leave 2D material surfaces lacking dangling bonds and intrinsically passivated^26,118^, facilitating their seamless integration with diverse optical waveguides and materials for high-performance photodetection^35,36,57,119–122^. In addition, their passivation nature helps avoid performance deterioration caused by surface effects, such as the defect-induced recombination of generated electrons and holes, which facilitates an increase in the photoelectric conversion efficiency of absorbed photons^123^. 3) Neighboring layers in 2D material heterostructures are connected with van der Waals forces, similar to their bulk counterparts^24,118,124,125^. This enables the creation of diverse vertical heterostructures by readily assembling various 2D materials, circumventing the challenges of lattice misalignment and growth temperature typically encountered in traditional heterojunctions^126^. 4) In contrast to infrared photodetectors made from traditional 3D bulk materials, those utilizing 2D materials typically display significantly reduced device sizes and hence lower noise levels, which enables the optical devices to achieve a large signal-to-noise ratio at ambient temperatures, eliminating the requirement for active cooling systems^127^. 5) 2D materials can be grown into wafer-scale, large-area thin films on various substrates, such as fluorophlogopite mica, sapphire, and gold, using the CVD method^128–131^, which is more economical and efficient than conventional epitaxial growth approaches utilized for preparing 3D bulk materials (e.g., In_*x*_Ga_1−*x*_As). Moreover, they can be transferred at the wafer scale onto basically any other substrate^132^. This capability allows seamless integration with traditional CMOS technology and PICs, enabling practical, real-world applications^133^.

However, in practical integrated photodetection applications, there remain several engineering issues that need to be carefully addressed for 2D materials. Despite recent progress toward wafer-scale fabrication, current synthesis and integration processes are still limited in terms of film uniformity and fabrication complexity^134^. At the same time, owing to their atomically thin nature, unavoidable structural defects in 2D materials (such as vacancies, dislocations, and grain boundaries) can reduce the photoluminescence quantum yield and carrier mobility, and may also prolong the response time of photodetectors through defect-induced trap states^135^. Substrate compatibility is another critical consideration. Although the absence of dangling bonds provides considerable freedom for heterogeneous integration, transfer-based approaches are prone to introducing wrinkles, blisters, and contamination^136^, whereas direct growth typically requires elevated temperatures and is difficult to implement on photonic integration platforms with a limited thermal budget^137,138^. Therefore, while leveraging the intrinsic advantages of 2D materials, it is essential to develop improved processing routes and integration strategies to address these challenges, which will be key to sustaining progress in waveguide-integrated infrared photodetectors.

### Categories of layered 2D materials utilized for infrared photodetectors

**Fig. 3:**
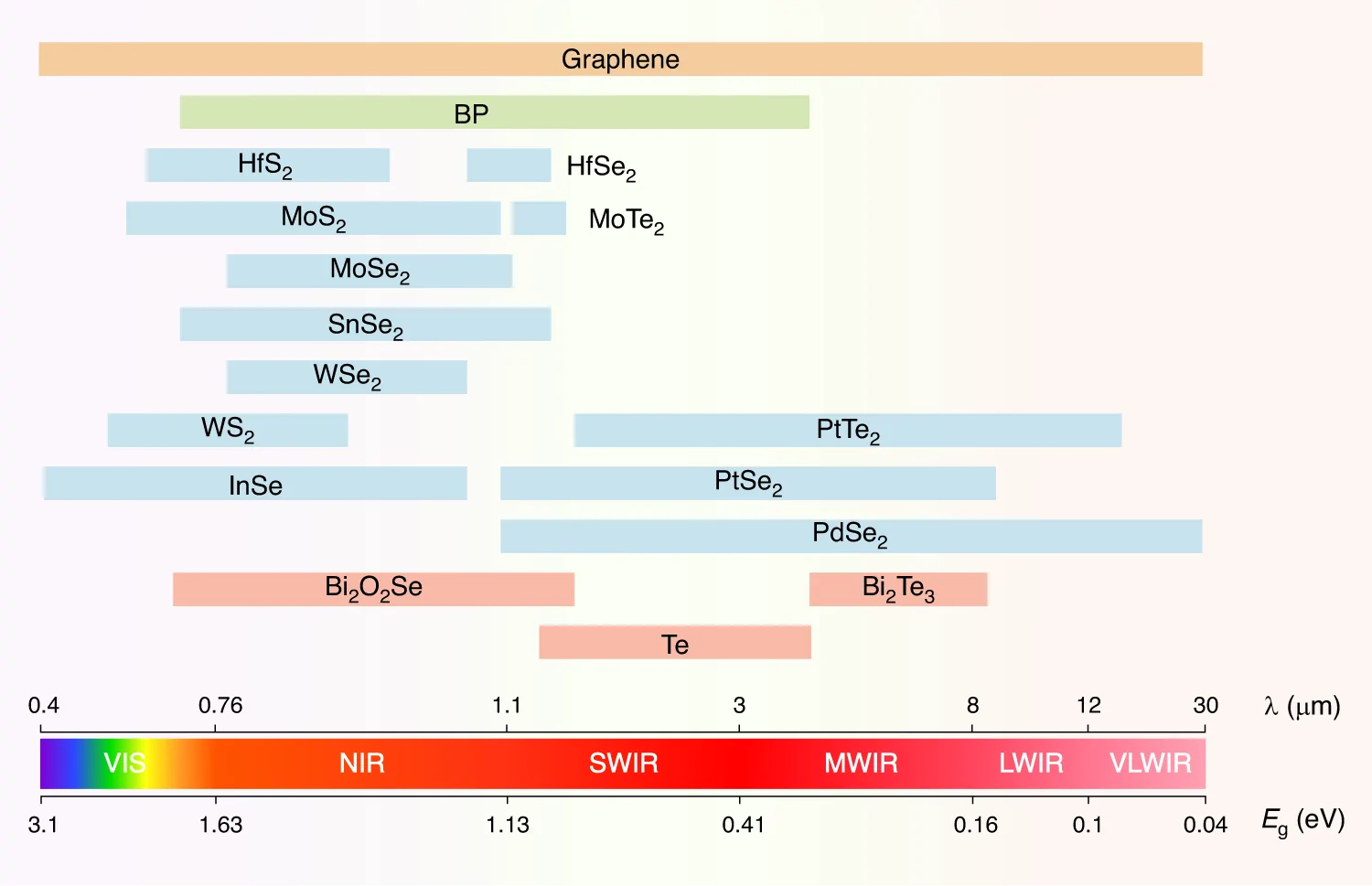
Spectral coverage of representative 2D infrared materials. The horizontal bars indicate the optical absorption wavelength ranges of representative 2D materials across the visible and infrared spectral regions.

Following graphene’s isolation in 2004^139^, researchers have synthesized and studied a vast array of 2D materials, yet only a select few are compatible with optical waveguide integration for high-performance infrared photodetectors^26,35,57^. The most widely utilized 2D materials for infrared sensing consist of graphene, BP, TMDs (such as MoTe_2_ and WS_2_), noble metal dichalcogenides (such as PtSe_2_, PtTe_2_, and PdSe_2_), and some other emerging 2D materials, such as tellurium (Te) and Bi_2_O_2_Se. Figure 3 provides an overview of the spectral range these materials can absorb and their associated bandgap ranges. The infrared band usually can be categorized into near-infrared (NIR, 0.8–1 µm), short-wave infrared (SWIR, 1–3 µm), mid-wave infrared (MWIR, 3–5 µm), long-wave infrared (LWIR, 8–12 µm), and very long-wave infrared (VLWIR, 12–30 µm) according to the wavelength range^26^.

Graphene was the inaugural 2D material employed for infrared sensing, and its electro-optic characteristics have been extensively researched^37,39,112^. It is a carbon allotrope in which carbon atoms are bound through sp^2^ hybridization, creating a monolayer of hexagonal honeycomb lattice, with interlayer connections facilitated by van der Waals forces^140,141^. Graphene exhibits exceptional stability, corrosion resistance, flexibility, a high melting point, and a ductile structure under mechanical stress. Graphene also exhibits low resistivity and exceptionally great carrier mobility, reaching up to 200,000 cm^2 ^V^−1^ s^−1^ at ambient temperature, and is less influenced by temperature, maintaining mobility of approximately 15,000 cm^2 ^V^−1^ s^−1^ at temperatures between 50 and 500 K, and enabling graphene to quickly convert photons into currents or voltages^142^. Moreover, graphene possesses distinctive optical characteristics, featuring a zero band gap that enables it to demonstrate an exceptionally broad spectral response range spanning frequencies from the visible spectrum to terahertz^112,143^. The absorption of graphene is primarily governed by interband transitions, enabling an absorbance of approximately 2.3% within the response spectral range^144^. Furthermore, the absorption characteristics of graphene are adjustable, and researchers frequently modify the Fermi energy level of graphene using electrical gating^144^, chemical doping^145^, geometric shearing^146^, or mechanical stretching^147^ to alter its absorption properties. Despite benefiting from its zero bandgap to achieve broadband detection, the gapless nature leads to a high intrinsic carrier density and large dark current, thereby constraining the ultimate detectivity of graphene-based photodetectors^148^. These drawbacks necessitate mitigation through heterostructure engineering or the design of novel device architectures^149^.

BP is another layered 2D material with light detection abilities^150,151^. The monolayer of BP displays a folded honeycomb structure in which each phosphorus atom is covalently connected to three adjacent atoms through sp^3^ hybridized orbitals^152^. The existence of two different orientations in the BP crystal (armchair and zigzag directions) leads to significant in-plane anisotropy in conductivity and photoconductivity^153^, distinguishing it from the semi-metallic characteristics of graphene. Additionally, BP has direct bandgap tunability, attainable by manipulating the number of layers, with bandgaps spanning 0.3–2 eV^116,154^. It can also form bulk alloys with arsenic (As_*x*_P_1−*x*_), which further reduce the bandgap to about 0.15–0.3 eV^155^, thereby enabling the development of photodetectors operating over a broad wavelength range^156^. Notably, BP also has an excellent carrier mobility, and at ambient temperature, its carrier mobility surpasses 1000 cm^2 ^V^−1^ s^−1^ ^154^. These characteristics make BP an excellent choice for optoelectronic and rapid mid-infrared photodetection applications. However, BP generally demonstrates poor chemical and physical stability, necessitating encapsulation or passivation to safeguard BP devices^157^.

In contrast to graphene and BP, TMDs constitute a category of 2D materials characterized by the sandwich configuration X-M-X, with a typical chemical formula represented as MX_2_, where M signifies a transition metal from groups IV–VI (e.g., Mo and W) and X represents a chalcogen element (e.g., S, Se, and Te)^158^. Most TMD semiconductors exhibit a bandgap spanning roughly 1.0–2.5 eV^57,159^, rendering them suitable for absorbing visible and near-infrared light, and this substantial bandgap also leads to a reduced dark current relative to graphene. In addition, the facile assembly of various TMDs can construct van der Waals heterojunctions with controllable energy level arrangement and hence realize highly efficient infrared sensing. For instance, 2D WS_2_ or MoTe_2_ flakes can be aligned with MoS_2_ to create a staggered-gap heterojunction^160,161^, enabling effective infrared photodetection through interactions between the conduction band minimum of one material and the valence band maximum of the other. Another subset of frequently studied 2D materials is highly air-stable noble metal dichalcogenides, such as PtSe_2_, PtTe_2_, and PdSe_2_^162–165^. These materials exhibit a relatively large bandgap in their monolayer form (1.2, 1.3, and 0.5 eV for single-layer PtSe_2_, PdSe_2_, and PtTe_2_, respectively), displaying semiconductor properties, whereas in their bulk form, the bandgap becomes extremely small, showing metallic or semi-metallic characteristics^162,164,165^. The significant layer-dependent transition in bandgap and physical properties, along with the small bandgap in multilayer forms (i.e., less than 0.1 eV), enables noble metal dichalcogenides to integrate with optical waveguides for realizing high-performance MWIR and LWIR photosensing^163,164,166^.

In addition, some emerging 2D materials, like Te and Bi_2_O_2_Se, have recently demonstrated significant promise for implementing efficient on-chip infrared detection^53,167,168^. Like BP, atomically thin 2D Te flakes display a thickness-dependent bandgap, covering the 0.31–1.04 eV range^169^, and possess a high carrier mobility of approximately 900 cm^2 ^V^−1^ s^−1^, which facilitates SWIR and MWIR photodetection with fast photoresponse^170^. But unlike BP, Te exhibits superior air stability for several months^171^. Moreover, Te can further expand the tunable range of its bandgap (between 0.31 eV of Te and 1.87 eV of Se) without altering its crystal structure by forming alloy materials with Se^172,173^. Such alloys can also be utilized to achieve SWIR photosensing. Another promising representative 2D material for infrared sensing is air-stable layered Bi_2_O_2_Se, which possesses a 0.8 eV indirect bandgap and an extremely large Hall charge mobility of roughly 29,000 cm^2 ^V^−1^ s^−1^ under ultralow temperature^168,174^. Its large charge mobility and narrow bandgap enable it to realize a large responsivity of 3.5 A W^−1^ under 1.26 μm incident light and a low noise of approximately 15.1 pW∙Hz^−0.5^ when integrated with silicon waveguides as SWIR photodetectors^168^. Table 2 summarizes and compares the main advantages, limitations, and application scenarios of representative 2D materials employed in waveguide-integrated infrared photodetectors.

**Table 2:** Advantages, disadvantages, and application scenarios of representative 2D materials utilized in waveguide-integrated infrared photodetectors

Category	Advantage	Disadvantage	Application scenario
Graphene	Broadband absorption, high mobility	Zero bandgap, relatively high dark current	High-speed broadband detectors
BP	Direct, tunable bandgap, in-plane anisotropy	Poor ambient stability	MIR and polarization-sensitive detection
TMDs	Tunable bandgap, low dark current	Relatively low mobility	Low-noise, wavelength-selective NIR detectors
Te	Narrow bandgap, good ambient stability	Relatively high dark current	SWIR and MWIR photodetection
Bi_2_O_2_Se	High mobility, good ambient stability	Complex synthesis	SWIR photodetection with low noise

*BP* black phosphorus, *TMDs* transition metal dichalcogenides, *Te* tellurium, *MIR* mid-infrared, *NIR* near-infrared, *SWIR* short-wave infrared, *MWIR* mid-wave infrared

## Fundamentals of waveguide-integrated 2D-material infrared photodetectors

**Fig. 4:**
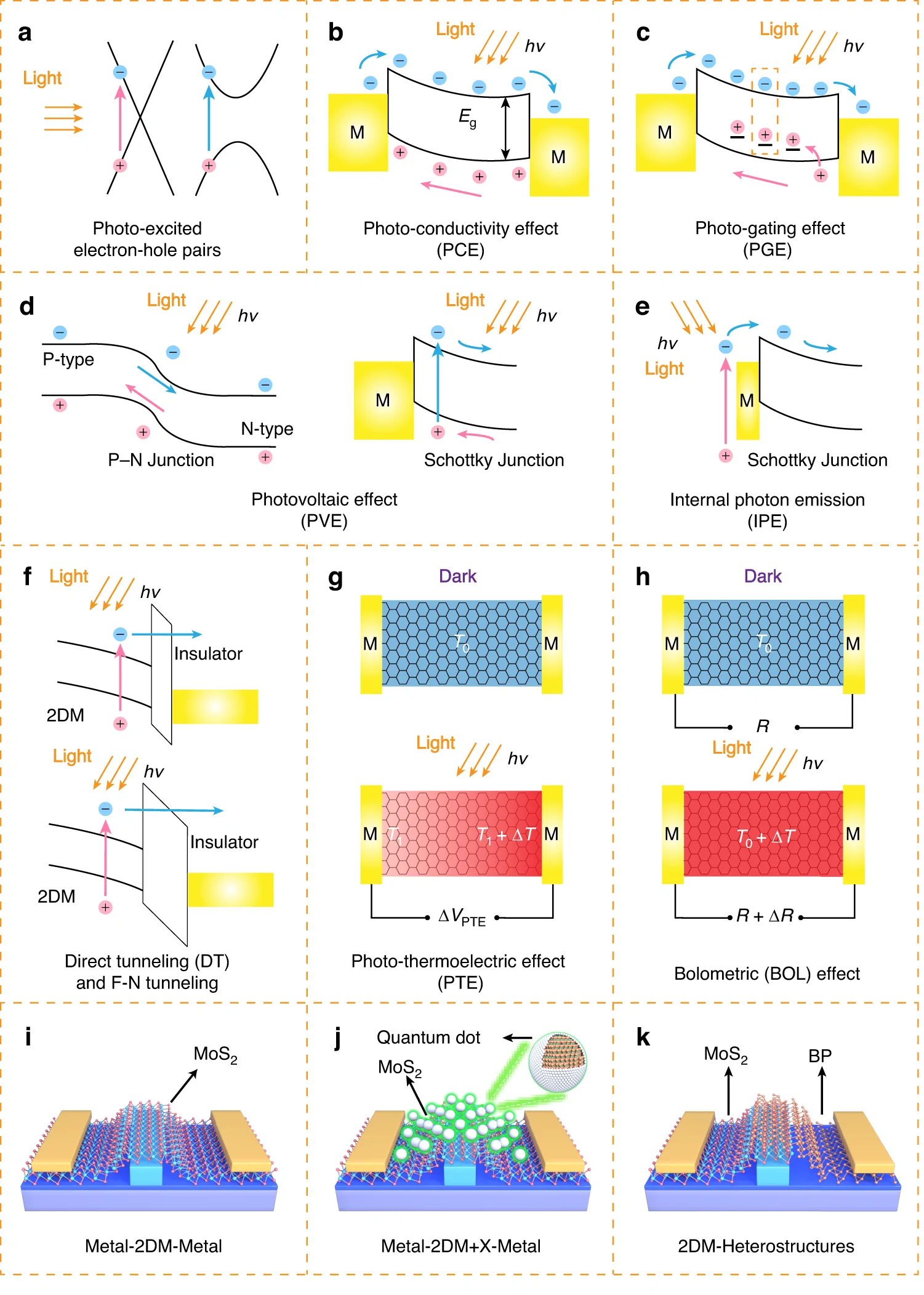
Schematic of the working mechanisms and device structures for waveguide-integrated 2D-material infrared photodetectors. **a** Schematic illustration of electron–hole pair generation by photoexcitation. **b–h** Schematic illustration of the PCE **b**, PGE **c**, PVE **d**, IPE **e**, DT and F-N tunneling **f**, PTE **g**, and BOL effect **h**, respectively. **i** Configurations of metal-2DM-metal, where the 2D material is MoS_2_. **j** Configurations of metal-2DM + X-metal, where the 2D material is MoS_2_ and X represents quantum dots. **k** Configurations of 2DM-heterostructures, where the heterojunction is composed of MoS_2_ and BP

Infrared photodetectors are optical components designed to sense infrared signals by transforming incoming infrared illumination into electrical signals (e.g., photocurrent), where the photoelectric conversion is achieved primarily through the electron-hole pair generation induced by single-photon absorption in the effective light-absorption region of 2D materials (Fig. 4a). In this part, the fundamentals of waveguide-integrated 2D-material infrared photodetectors are outlined, including working mechanisms, device structures, and performance parameters. First, according to the presence or absence of thermal effects, infrared photodetectors typically operate via two categories of working mechanisms, one is the generation of free charges through light excitation, consisting of the photo-conductivity effect (PCE), photo-gating effect (PGE), photovoltaic effect (PVE), internal photon emission (IPE), direct tunneling (DT), and Fowler-Nordheim (F-N) tunneling, the other is variable electrical signals and hence infrared sensing caused by thermal effects, composed of the photo-thermoelectric effect (PTE) and bolometric (BOL) effect. Then, Several common waveguide-integrated photodetector structures are summarized and compared. Finally, we present the essential performance parameters of infrared photodetectors to facilitate a more effective comparison of devices with various structures and working mechanisms.

### Working mechanisms

#### PCE

In the photo-conductivity mechanism, when the photodetector operates in darkness, the number of free charges in the semiconductor channel that can be driven by an external bias voltage is limited, resulting in only a small dark current. However, under infrared irradiation, if the photon energy surpasses the bandgap of the semiconductor, a large number of carriers excited by light can be effectively separated and transported under the drive of external source-drain bias, which can dramatically increase the density of free charges in the infrared semiconductor channel, thereby enhancing its conductivity, and generate a large photocurrent^175,176^ (Fig. 4b). The channel conductivity increases by Δ*σ* = Δ*neμ*, where Δ*σ*, Δ*n*, *e*, and *μ* represent changes in channel conductance, changes in charge concentration, elementary charge, and charge mobility, respectively, due to the plenty of carriers excited by light^36^. Crucially, the generation of photocurrent in the photo-conductivity mechanism necessarily requires the application of an external voltage^177^.

Photoconductive infrared materials are capable of achieving a photoconductive gain exceeding one. The photoconductive gain can be explained by noting that, in the majority of semiconductors, electrons exhibit substantially higher mobility compared to holes. As a result, the free electrons excited by light can be transported to the electrodes much more quickly than free holes when a bias voltage is applied. To ensure carrier neutrality, electrons need to repeatedly circulate within the semiconductors multiple times before eventually recombining with holes that have been collected at the electrodes, resulting in an increased tested photocurrent. Therefore, the photoconductive gain is defined as the ratio of the photocarrier lifetime to the electron transit time, and a large photoconductive gain can be attained by increasing the lifetime of charges excited by light or decreasing the electron transit time^57^. Nevertheless, an extended lifetime of charges excited by light indicates that the time for electrons and holes to recombine is prolonged, which can hinder the rapid photoresponse of infrared photodetectors. Thus, it is essential to fine-tune these parameters to achieve a balanced photoconductive gain and response time.

#### PGE

The PGE is a specific type of PCE that is commonly seen in infrared sensing materials. Under light irradiation, the active region of 2D materials effectively absorbs photons, generating numerous electron-hole pairs. If these separated electrons or holes are captured by surface defect states within the 2D material, the trapped carriers can function as confined floating gates, regulating the resistance of the semiconducting channel and thereby producing a significant photocurrent^178,179^ (Fig. 4c). Free electrons (or free holes) in the semiconductor channel are consecutively cycled and moved until they recombine with trapped holes (or electrons), giving this type of photodetector a very large photoconductive gain^111^. Nevertheless, the detrapping process of the captured charges is slow, leading to an extended charge lifetime and a delayed recombination process. As a result, photodetectors based on the PGE usually exhibit slower response speeds, usually within the millisecond range.

#### PVE

According to Fig. 4d, in the case of PVE, the internal electrical field created by the depletion region of the p–n junction^111^ or the Schottky junction^180^ at the semiconductor-metal boundary can effectively split and transport electron-hole pairs generated under infrared illumination and hence determine the direction of the photocurrent. In 2D infrared sensing materials, the internal electrical field can be established through methods such as assembling 2D heterojunctions, split-gate modulating, chemical doping, and utilizing drain and source metal electrodes with varying Fermi levels^181–183^. The output characteristics of these PVE-based photodetectors exhibit obvious nonlinear rectifying behavior. Under light exposure, when light energy exceeds the 2D material’s bandgap, the incident photons are efficiently absorbed, generating numerous charge carriers. These carriers move in a direction determined by the internal electrical field, resulting in the short-circuit current observed in the rectifying characteristics^184^. When the short-circuit current is zero, electrons and holes will be collected on both sides of the photodetector, generating an open-circuit bias. Due to the internal electrical field, this kind of infrared device typically operates at zero or reverse voltage polarity, allowing for small dark current as well as enhanced photoelectric conversion efficiency. In specific situations, for instance, avalanche photodiodes^185^, if a substantial reverse polarity voltage is applied to a p-n diode with a high concentration of dopants, the intense internal electrical field can accelerate the creation of a huge number of charges, which can lead to more free charges produced by impact ionization, thus causing a large photoconductive gain for detecting dim optical signals and single photons.

#### IPE

IPE, commonly referred to as thermionic emission, serves as the fundamental operation principle for conventional Schottky infrared photodetectors that contain Schottky junctions formed at the semiconductor-metal interface. In this type of photodetector, the hot charges excited by light possessing adequate energy can overstep the Schottky potential barrier and hence induce a large photocurrent^180^ (Fig. 4e). However, during the continuous optical absorption process within the metal, only a very minor fraction of the created hot electrons possess a momentum component perpendicular to the interface with enough energy to surpass the Schottky potential barrier and produce an effective photocurrent^186^. Consequently, the internal quantum efficiency of this type of photodetector is relatively small, usually less than 1%^36^.

#### DT and F-N tunneling

DT and F-N tunneling are often utilized to explain the working principles of conventional floating gate memory and several other optical devices with metal-insulator-semiconductor configurations^187,188^ (Fig. 4f), including infrared photodetectors utilizing 2D materials and metal tunneling diodes. When the insulating layer is sufficiently thin, DT can occur, allowing photoexcited charges to pass directly through the insulating layer to reach the metal side^188^. In the case of F-N tunneling, the numerous charges excited by light can traverse the triangular potential barrier of the dielectrics, and this process typically requires a large external electrical bias^188^. In these types of tunneling photodetectors, the dielectrics can efficiently lower the dark current (around nA) while increasing the signal-to-noise ratio.

#### PTE

PTE refers to a thermal-related mechanism caused by exposure to light. As exhibited in Fig. 4g, when the light beam is narrower than the channel size of the photodetector, the focused optical irradiation generates a temperature variation (Δ*T*) between diverse regions or both terminals of the device channel, which can create a photovoltage, enabling photocurrent to flow through the semiconductor channel with no external bias applied^189^. At the microscopic level, absorbed photons first excite charge carriers to higher energy states. These photoexcited carriers then rapidly relax back towards the Fermi level via electron–electron and electron–phonon scattering, forming a hot-carrier distribution and locally elevating the electronic temperature^190^. The resulting temperature gradient drives electrons or holes to diffuse from the hot region to the cold region and, in the presence of a spatially varying Seebeck coefficient, this thermally driven carrier diffusion establishes a lateral thermoelectric potential, referred to as the PTE-induced photovoltage. The photovoltage produced by PTE can be formulated as follows:

1 $${V}_{{{\rm{photo}}}}=({S}_{2}-{S}_{1})\Delta T$$

in which *V*_photo_, *S*_2_, *S*_1_, and Δ*T* represent the photovoltage induced by PTE, Seebeck coefficients, and temperature gradient, respectively. These Seebeck coefficients are described by the equation below:

2 $$S=-\frac{{\pi }^{2}{k}_{{{\rm{B}}}}^{2}T}{3q\sigma }\frac{\partial \sigma }{\partial \varepsilon }$$

in which *S*, *k*_B_, *T*, *q*, *σ*, and *ε* represent the Seebeck coefficient, Boltzmann constant, electron temperature, elementary charge, electrical conductivity, and Fermi energy, respectively. In addition to localized light illumination, temperature gradients can also arise from global optical irradiation if there is a substantial disparity in optical absorption across various regions of the photodetector. Typically, the value of photovoltage produced by PTE is relatively low, ranging from tens of microvolts to tens of millivolts, and thereby the photoresponse caused by PTE can be basically ignored in comparison with other photoconductive photodetectors that require external bias^45^.

#### BOL effect

As illustrated in Fig. 4h, the BOL effect occurs when a thermal-sensing 2D material undergoes conductivity variations due to uniform heating induced by optical absorption. When exposed to heat radiation, the conductance of the channel may either rise or fall, causing a corresponding increase or decrease in current flow through the photodetector under an applied external bias^191^. The essential parameters of the BOL effect include the thermal resistance, quantified by the ratio between temperature change and optical absorption, and shows the photothermal sensitivity of the device, and the heat capacity, whose product with thermal resistance governs the response speed of the device. The key distinction between PTE and the BOL effect is that PTE produces a self-powered photocurrent, similar to the aforementioned photodetectors based on PVE, whereas the BOL effect requires an applied voltage to generate any observable photocurrent. In addition, the polarity of the photocurrent also varies between these two effects. The BOL effect relies on conductivity changes induced by temperature variations. Nevertheless, for PTE, the polarity of the photocurrent depends on the variation in Seebeck coefficients across diverse channel areas. The BOL effect has been realized in several 2D material devices, such as graphene with a high concentration of dopants and BP, and it is particularly significant under high-voltage operation^181,192^. Moreover, since the thermal-related mechanism is not influenced by wavelength^193^, the BOL effect can also be applied in waveguide-integrated photodetector devices to enable broadband light sensing, surpassing the constraints of the semiconductor’s bandgap. For instance, the 2D SnSe with the BOL effect could sense light (10.6 μm wavelength) below its bandgap with a decent responsivity^194^.

#### Brief discussion on working mechanisms

Importantly, the photocurrent production of waveguide-integrated 2D-material infrared photodetectors often arises from the combined effects of several working mechanisms. For instance, the BOL effect is a common mechanism in diverse infrared sensing devices because the large number of photons generated by optical absorption can cause the lattice of 2D materials to oscillate, which in turn alters its resistance and unavoidably produces photocurrent. In practice, even for photodetectors nominally designed to operate *via* nonthermal mechanisms, such as PCE, PGE, and PVE, thermal-related processes are often inevitably involved. In general, thermal-related mechanisms governed by lattice heating and thermal diffusion tend to introduce slower response components compared with carrier-dominated nonthermal processes. Notably, recent studies have demonstrated that the PTE effect, although thermal in origin, can exhibit ultrafast photoresponse when dominated by hot-carrier dynamics rather than lattice heat diffusion. In particular, time-resolved electrical measurements have revealed that PTE photocurrent generation and decay can occur on picosecond timescales, corresponding to bandwidths exceeding 200 GHz in graphene-based photodetectors^195^. Despite such exceptions, response-speed analysis remains a useful empirical criterion for identifying dominant photocurrent generation mechanisms. In particular, frequency-dependent photoresponse measurements can provide valuable insights when distinguishing lattice-heating-dominated thermal effects from carrier-driven nonthermal processes, provided that the underlying physical mechanisms are carefully considered.

### Device structures

Since Avouris et al. first demonstrated a metal-graphene-metal photodetector in 2009^194^, many devices incorporating different 2D materials and architectures have been gradually developed and investigated. In this section, the different device structures of 2D material-based photodetectors are introduced and compared, and, generally speaking, there are three most common photodetector structures. The first category is the most common and basic structure, which consists of a 2D material and two contact electrodes (Fig. 4i), and may also have one or several gates to electrostatically control the carrier concentration of the channel.

The second type is also composed of 2D semiconductor materials and two contact electrodes in contact with them. Additionally, the 2D semiconductor is in contact with other materials located above it (Fig. 4j), such as quantum dots^178,196^ and 3D bulk materials^197,198^. In this structure, both the 2D semiconductor channel and the assembled materials above it have the potential to absorb optical signals. Typically, the material above the 2D semiconductor, for instance, quantum dots, can serve as sensitizers to absorb incoming optical signals, while the semiconductor channel below can transfer photogenerated charges to the electrodes at both ends. In this configuration, under light illumination, the hole-electron pairs produced by the quantum dots are segregated, and one of them, such as holes, is transferred to the semiconductor channel, while the other, for instance, electrons, is retained and captured in the quantum dots. These trapped carriers can function as confined floating gates, similar to the aforementioned PGE, regulating the resistance of the semiconducting channel and significantly increasing the gain of the device, particularly under dim light environments^199^. However, this structure has been rarely reported in waveguide-integrated on-chip infrared photodetectors and still requires further research and exploration.

The third category is the heterojunction configuration, where the 2D semiconductor and another material (e.g., another kind of 2D material) are in contact with the source and drain metal electrodes, respectively (Fig. 4k). In this structure, the heterojunction combination of different materials can lead to various energy level arrangements at the interface, which can effectively regulate the transfer dynamics of interface charges and thus realize high-performance infrared photodetectors.

### Performance parameters

To facilitate a more effective comparison of photodetector performance across varying dimensions and measurement conditions, several performance parameters have been established. In the subsequent part, we will provide a brief overview of these key parameters.

#### Responsivity

Responsivity is characterized as the ratio of the electrical current or bias generated by light illumination to the incoming optical power, described by the following equations:

3 $${R}_{{{\rm{I}}}}=\frac{{I}_{{{\rm{p}}}}}{P}$$

4 $${R}_{{{\rm{V}}}}=\frac{{V}_{{{\rm{p}}}}}{P}$$

in which *R*_I_ refers to the current responsivity, *I*_p_ is the electrical current generated by light illumination, *P* represents the incoming optical power, *R*_V_ is the voltage responsivity, and *V*_p_ represents the electrical bias produced by light irradiation. Apart from photodetectors based on the PVE, which exhibit an open-circuit bias under light irradiation and hence can be assessed by voltage responsivity, most photodetectors are solely evaluated utilizing current responsivity. The current responsivity is given by the equation:

5 $${R}_{{{\rm{I}}}}=\frac{{N}_{{{\rm{C}}}}e\lambda }{{N}_{{{\rm{I}}}}hc}$$

in which *N*_C_, *e*, *λ*, *N*_I_, *h*, and *c* represent the quantity of photogenerated carriers produced by the photodetector, electron charge, the wavelength of incoming optical signals, the number of incoming photons, Planck constant, and light speed, respectively. From this equation, it can be observed that under the condition of a constant ratio between *N*_C_ and *N*_I_, the device’s responsivity gradually increases as the wavelength of the incoming optical signals becomes longer. However, when the wavelength increases to the point where its corresponding energy is less than the 2D semiconductor’s bandgap, the optical absorption efficiency of the photodetector will significantly decrease, leading to a notable reduction in responsivity.

#### Quantum efficiency (QE)

QE can be separated into internal QE (IQE) and external QE (EQE). EQE can be expressed as the ratio of the quantity of photogenerated carriers produced by the photodetector (*N*_C_) to the quantity of photons incident on it (*N*_I_), which can be described as follows:

6 $${{\rm{EQE}}}=\frac{{N}_{{{\rm{C}}}}}{{N}_{{{\rm{I}}}}}=\frac{hcR}{e\lambda }$$

It is worth mentioning that some incoming photons are often not effectively absorbed due to inevitable optical dissipation, and the number of effectively absorbed incident photons may be represented by the equation below:

7 $${N}_{{{\rm{A}}}}=\eta {N}_{{{\rm{I}}}}={N}_{{{\rm{I}}}}(1-R-T)$$

in which *N*_A_, *η*, *R*, and *T* represent the quantity of absorbed incident photons, absorption coefficient, reflection, and transmission, respectively. Differently, IQE is described as the ratio of the quantity of photogenerated carriers produced by the photodetector (*N*_C_) to the quantity of photons absorbed by it (*N*_A_), which can be represented by the equation below:

8 $${{\rm{IQE}}}=\frac{{N}_{{{\rm{C}}}}}{{N}_{{{\rm{A}}}}}=\frac{{{\rm{EQE}}}}{\eta }$$

IQE primarily originates from the inner dissipation effect of photons in the 2D material, for instance, unavoidable charge recombination and parasitic losses. The majority of commercially available infrared photodetectors exhibit a large IQE over a broad wavelength range. For instance, for infrared devices based on Hg_*x*_Cd_1-*x*_Te, the IQE exceeds 90% across a broadband light absorption (1–14 μm)^200^.

#### Response time

Response time is a critical performance metric for various infrared photodetectors, which describes the response speed of the device under light illumination. It usually consists of the rise time and fall time, generally measured as the duration taken for the generated photocurrent to shift between 10% and 90% or 90% and 10%, respectively, under external light pulses. The response time of infrared photodetectors is mainly dominated by the 2D semiconductor used and the working principle. For instance, for PCE-based photodetectors, the response speed mainly depends on charge mobility, the distance between electrodes, and the applied voltage. For PVE-based photodetectors, the response speed is primarily dominated by charge mobility and the magnitude of the built-in electric field. For BOL effect-based photodetectors, the response speed is mostly affected by the photothermal response, and the rate of conductivity changes with temperature. Moreover, the intrinsic defects within the semiconducting channel can also impact the response speed of infrared photodetectors. The response duration of commercial infrared devices is generally in the order of ns; for instance, the response durations of photodetectors based on InSb and HgCdTe are approximately 30 and 500 ns, respectively. In contrast, the infrared device based on graphene can realize an ultrafast response speed of about 34 ns in the communication band^201^.

#### Bandwidth

For the majority of infrared photodetectors, their responsivity is mainly dominated by the optical modulation frequency, which may be represented by the following equation:

9 $$R(f)=\frac{{R}_{0}}{\sqrt{1+{(2\pi f\tau )}^{2}}}$$

in which *R*_0_, *f*, and *τ* represent the responsivity measured when light is constantly shining on the infrared photodetector, optical modulation frequency, and time parameter, respectively. As can be seen, as the optical modulation frequency continues to increase, the responsivity of the photodetector will gradually reduce. When the optical frequency increases to the point where the responsivity of the device drops to 0.707*R*_0_, this frequency is defined as the response bandwidth of the device.

Accordingly, using Eq. (9), the response bandwidth can be written as:

10 $${f}_{{{\rm{bw}}}}=\frac{1}{2\pi \tau }$$

In particular, when the response is dominated by the electrical charging and discharging of the detector and readout circuit, *τ* corresponds to the RC time constant, leading to an RC-limited bandwidth *f*_RC_ = 1/(2πRC). Alternatively, when carrier transport across the active region is the dominant limiting factor, *τ* is governed by the carrier transit time *τ*_tr_, resulting in a transit-time-limited bandwidth *f*_tr_ = 1/(2π*τ*_tr_)^202^.

If the frequency response of certain infrared photodetectors closely resembles that of a low-pass resistor-capacitor circuit, the bandwidth and response duration of the device may be calculated via the following formula:

11 $${f}_{{{\rm{bw}}}}=\frac{0.35}{{\tau }_{{{\rm{r}}}}}$$

in which *f*_bw_ and *τ*_r_ represent the bandwidth and response time of the device, respectively.

#### Photoconductive gain

Photoconductive gain serves as a metric to assess the capability of producing multiple charges from an absorbed photon, as shown in the equation below:

12 $$G=\frac{{\tau }_{{{\rm{life}}}}}{{\tau }_{{{\rm{tran}}}}}$$

in which *G*, *τ*_life_, and *τ*_tran_ represent the photoconductive gain, lifetime of carriers excited by light, and transit time of charges, respectively. *τ*_tran_ can be further described by the following formula:

13 $${\tau }_{{{\rm{tran}}}}=\frac{{L}^{2}}{\mu {V}_{{{\rm{bias}}}}}$$

where *L*, *μ*, and *V*_bias_ refer to the distance between two electrodes, charge mobility, and the implemented external bias, respectively. As described in Section “PCE”, a large photoconductive gain can be attained by extending the lifetime of charges excited by light or decreasing the electron transit time. However, the carrier lifetime should not be too long; otherwise, it would influence their recombination procedure, finally resulting in a very slow response time.

#### Noise

When fabricating high-performance infrared devices, noise is a key factor to consider. Typically, the noise in infrared photodetectors can be divided into four different categories, including Johnson (or thermal) noise, photon noise, flicker noise, and shot noise^63^. The first type is Johnson noise, which occurs in every resistor and is caused by the movement of charges inside the material caused by the heat effect. The electrical current induced by Johnson noise is represented by the equation below:

14 $${i}_{{{\rm{N}}},{{\rm{Johnson}}}}={\left(\frac{4kT\Delta f}{R}\right)}^{0.5}$$

in which *i*_N, Johnson_, *k*, *T*, Δ*f*, and *R* represent the Johnson noise current, Boltzmann constant, measured temperature of the device, obtained bandwidth, and tested resistance of the device, respectively. The second category is photon noise, which refers to the impact of variations in light illumination from the external background on the performance of infrared photodetectors. Photon noise originates from external sources rather than the photodetector itself, making it unavoidable and capable of determining the ultimate photosensing performance of the device. The third type is flicker noise, which is commonly observed in 2D semiconductors. It gradually decreases as the frequency increases and typically originates from the stochastic capture and release of carriers. The electrical current induced by flicker noise can be expressed as below:

15 $${i}_{{{\rm{N}}},{{\rm{flicker}}}}=\sqrt{{\int }_{0}^{\Delta f}K\frac{{I}^{n}}{{f}^{m}}df}$$

in which *i*_N, flicker_, *K*, *I*, *n*, *f*, and *m* represent the flicker noise current, the flicker constant associated with the characteristics of materials, the dark current of the device, an exponential power roughly equivalent to 2, the frequency, and an exponential power roughly equivalent to 1, respectively. To minimize *i*_N, flicker_, the modulation frequency of the device should be large enough. The fourth category is shot noise, and the reasons for its generation vary across different devices. Shot noise arises from the stochastic arrival of charges in the junction region of diode devices or the stochastic production recombination of charges in photoconductive devices. The electrical current induced by shot noise in various photosensing devices can be expressed as below:

16 $${i}_{{\rm{N}}},\,{{\rm{shot}}},\,{{\rm{photodiode}}}={(2Ie\Delta f)}^{0.5}$$

17 $${i}_{{\rm{N}}},\,{{\rm{shot}}},{{\rm{photoconductor}}}={(4IeG\Delta f)}^{0.5}={(4IeR\Delta f)}^{0.5}$$

where *I*, *e*, *G*, and *R* represent the dark current of the device, electron charge, charge production rate, and charge recombination rate, respectively.

**Fig. 5:**
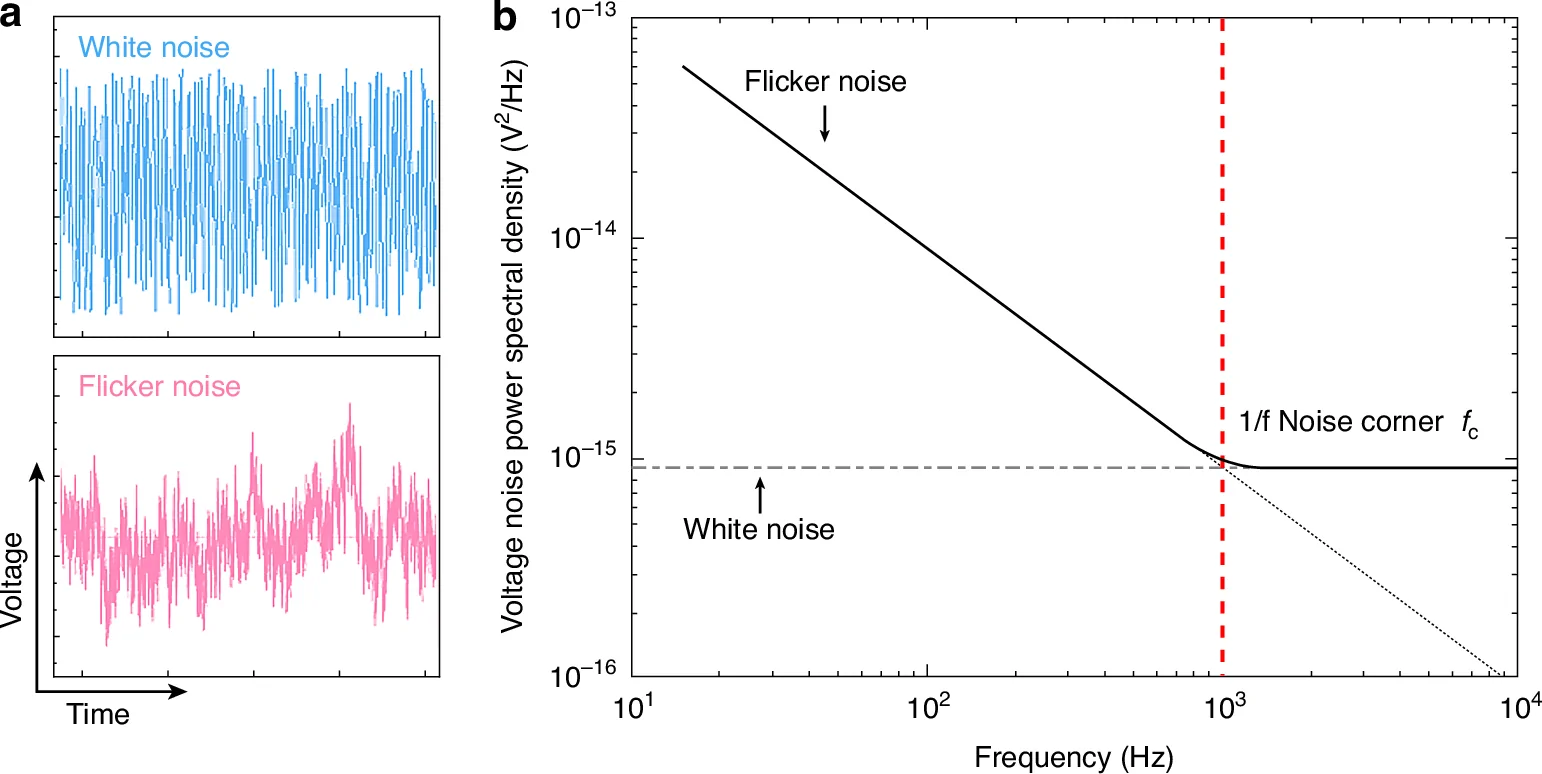
White and flicker noise in the time and frequency domains. **a** Representative time-domain voltage trace. The upper panel shows white noise, and the lower panel shows flicker noise^205^. Reproduced from ref. ^205^ under (CC BY 4.0). **b** Corresponding noise power spectral densities, showing a flat white-noise floor and a 1/*f*-like flicker-noise spectrum that dominates at low frequencies^206^. Reproduced from ref. ^206^ under (CC BY 4.0)

In practical photodetectors, Johnson noise, photon noise, and shot noise are commonly regarded as frequency-independent white-noise components within the relevant measurement bandwidth, and they exhibit an approximately constant power spectral density^203,204^. As a result, their time-domain voltage (or current) traces are statistically similar, as schematically illustrated in the upper panel of Fig. 5a. The corresponding fluctuations are centered around a zero mean and consist of rapid and irregular jitter without a noticeable baseline drift or long-term trend. For brevity, these three contributions are collectively referred to as white noise in the following discussion. By contrast, flicker noise contains a substantially stronger low-frequency component and gives rise to pronounced long-range temporal correlations^205^. A representative time trace is shown in the lower panel of Fig. 5a. In this case, the baseline varies slowly over time and leads to evident long-term drifts, while intermittent larger excursions are superimposed on a weaker high-frequency jitter. Compared with white noise, the waveform therefore evolves more slowly and appears less stationary, consistent with the increased spectral weight at low frequencies. These distinctions are more clearly captured in the frequency domain, as summarized in Fig. 5b. The power spectral density of white noise remains essentially flat over the frequency range of interest, forming an approximately constant noise floor. In contrast, flicker noise follows an approximate 1/*f* dependence at low frequencies. The two spectra intersect at the 1/*f* corner frequency, *f*_c_. Below *f*_c_, detector performance is primarily limited by flicker noise, whereas above *f*_c_, it is dominated by white-noise contributions^206^.

#### Noise equivalent power (NEP)

NEP refers to the smallest optical power that an infrared photodetector can sense or differentiate from the overall noise and is usually given by the following formula:

18 $${{\rm{NEP}}}=\frac{{i}_{{{\rm{N}}}}}{R}$$

in which *i*_N_ and *R* represent the electrical current induced by noise and the responsivity of the photodetector, respectively. From the formula, it can be seen that the unit of NEP should be W Hz^−1/2^. *i*_N_ can be further assessed and calculated based on the following equation:

19 $${i}_{{{\rm{N}}}}=\sqrt{{\int }_{0}^{\Delta f}{S}_{{{\rm{f}}}}df}$$

where *S*_f_ and Δ*f* represent the spectral density of the current noise power of the device and the obtained bandwidth, respectively. Moreover, *i*_N_, excluding photon noise, can be evaluated and calculated using the equation provided below:

20 $${i}_{{{\rm{N}}}}= 	\sqrt{{i}_{{{\rm{N}}},\,{{\rm{Johnson}}}}^{2}+{i}_{{{\rm{N}}},\,{{\rm{flicker}}}}^{2}+{i}_{{{\rm{N}}},\,{{\rm{shot}}},\,{{\rm{photodiode}}}}^{2}}\\ = 	\sqrt{\frac{4kT\Delta f}{R}+{\int }_{0}^{\Delta f}K\frac{{I}^{n}}{{f}^{m}}df+2Ie\Delta f}$$

As mentioned in Section “Advantages of layered 2D materials for infrared photodetectors”, in contrast to infrared photodetectors made from traditional 3D bulk materials, those utilizing 2D materials typically display significantly reduced device sizes and hence lower noise levels, which enables the optical devices to get an excellent signal-to-noise ratio at ambient temperatures.

#### Specific detectivity (D*)

Specific detectivity (D*), which indicates the photosensitivity of infrared devices, is among the key performance metrics. It is commonly expressed by the equation below:

21 $${D}^{\ast }=\frac{\sqrt{A\Delta f}}{NEP}=\frac{\sqrt{A\Delta f}R}{{i}_{{{\rm{N}}}}}$$

in which *A* refers to the active area of infrared devices. From this formula, it can be observed that the unit of D* is cm Hz^0.5^ W^−1^ (Jones), and D* can effectively eliminate the impact of bandwidth, size, and the active area of the photodetector on the device performance, thereby enabling a better contrast of the performance differences between various photodetectors. If the noise current originates solely from shot noise and the device structure is that of photodiodes, D* can be further simplified into the following formula:

22 $${D}^{\ast }=\sqrt{\frac{A}{2Ie}}R$$

The D* values of commercial infrared devices are typically on the order of ~10^12^ Jones, which is quite similar to that of some infrared devices based on 2D semiconductors.

#### Linear dynamic range (LDR)

Linear dynamic range (LDR) is commonly expressed through the following equation:

23 $$LDR=20\,\log \frac{{I}_{{{\rm{p}}}}}{{I}_{{{\rm{dark}}}}}$$

in which *I*_p_ and *I*_dark_ represent the generated photocurrent under a power density of 1 mW cm^−2^ and the observed dark current of the device, respectively. The LDR values of commercially available infrared photodetectors based on InGaAs and Si are approximately 66 and 120 dB, respectively^207^.

## Progress in waveguide-integrated 2D-material infrared photodetectors

To address the inherent limitations in the light absorption efficiency of atomically thin 2D materials, waveguide-integrated 2D-material photodetectors present an ideal solution. This innovative design significantly enhances the light-matter interaction length through waveguide structures, thereby enhancing light absorption effectively. With the further strengthening of light-matter interactions, waveguide-integrated 2D-material infrared photodetectors demonstrate promising potential for next-generation PICs, particularly in optical communications and interconnect applications. In the subsequent sections, this review presents an extensive overview of the latest progress in waveguide-integrated 2D-material infrared photodetectors, categorized according to different waveguide platforms.

### Silicon waveguides

#### Graphene

**Fig. 6:**
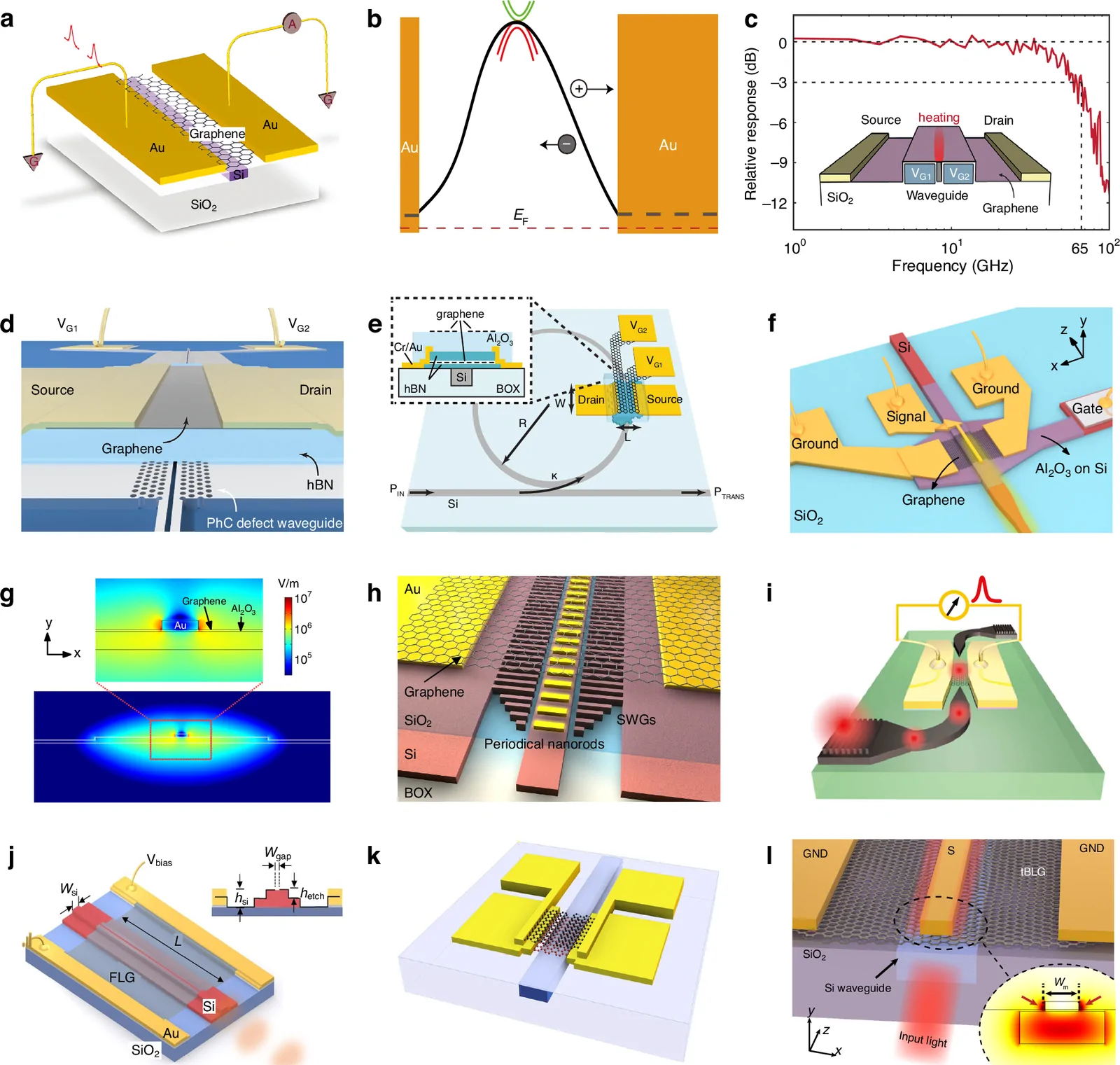
Graphene infrared photodetectors based on silicon waveguides. **a** The structure of a graphene-integrated photodetector utilizing a rectangular silicon waveguide^39^. Reproduced from ref. ^39^ with permission of Springer Nature. **b** Carrier transport process under the built-in electric field^39^. Reproduced from ref. ^39^ with permission of Springer Nature. **c** Structural schematic and bandwidth characteristics of a graphene photodetector employing a silicon slot waveguide^71^. Reproduced from ref. ^71^ with permission of Copyright © 2016 American Chemical Society. **d** Graphene photodetector configuration employing a photonic crystal defect silicon waveguide^80^. Reproduced from ref. ^80^ with permission of Copyright © 2018, American Chemical Society. **e** Schematic of a graphene photodetector implemented using a silicon microring resonator^47^. Reproduced from ref. ^47^ under (CC BY 4.0). **f** Architecture of a graphene photodetector utilizing a wide metal strip to induce surface plasmon polariton waveguide modes^45^. Reproduced from ref. ^45^ under (CC BY 4.0). **g** Surface plasmon polariton waveguide modes induced by metallic nanostructures^45^. Reproduced from ref. ^45^ under (CC BY 4.0). **h** The structure of a graphene photodetector using periodic metal strips to induce surface plasmon polariton waveguide modes^213^. Reproduced from ref. ^213^ with permission of Copyright © 2024 American Chemical Society. **i** Graphene photodetector integrated with a surface plasmon polariton slot waveguide^62^. Reproduced from ref. ^62^ under (CC BY 4.0). **j** Architecture of a graphene photodetector incorporating the metal-graphene-metal sandwich contact^66^. Reproduced from ref. ^66^ with permission of John Wiley and Sons. **k** The structure of a graphene photodetector incorporating a split-gate electrostatic modulation design^48^. Reproduced from ref. ^48^ under (CC BY 4.0). **l** Architecture of a twisted bilayer graphene photodetector integrated with the rectangular silicon waveguide^55^. Reproduced from ref. ^55^ with permission of Springer Nature

Silicon waveguides, benefiting from their suitability for established CMOS fabrication techniques and a high refractive index of 3.4 that enables efficient optical field confinement within the waveguide core, have been widely utilized in on-chip optoelectronic devices^208^. Meanwhile, graphene, as a widely studied 2D material, exhibits wideband light absorption covering the ultraviolet to the terahertz range, along with an extraordinary carrier mobility of up to ~10^6 ^cm^2 ^V^−1^ s^−1^^189^. Leveraging these properties, waveguide-integrated graphene-based infrared photodetectors were among the first to be successfully demonstrated by researchers. In 2013, three research teams independently demonstrated waveguide-integrated graphene-based infrared photodetectors^37–39^. Pospischil et al. demonstrated a CMOS-compatible graphene photodetector integrated onto silicon waveguides, employing a ground-signal-ground (GSG) electrode configuration and highlighting ultrafast response and broadband operation across all telecommunication bands^37^. In contrast, Wang et al. focused on a graphene/silicon heterostructure waveguide photodiode, where the built-in junction played a critical role in suppressing dark current and enhancing responsivity, particularly toward longer infrared wavelengths^38^. Notably, Gan et al. adopted an asymmetric metal-doped graphene junction coupled to a silicon waveguide, enabling efficient photocarrier separation under zero-bias operation while simultaneously achieving high responsivity and ultrafast bandwidth^39^. As shown in Fig. 6a, a 53 μm-long bilayer graphene sheet was transferred onto a rectangular silicon waveguide and contacted by asymmetric metal electrodes. The resulting asymmetric Schottky contacts establish a built-in electric field across the graphene channel, allowing the photodetector to operate at zero bias with a frequency response exceeding 20 GHz. Figure 6b illustrates the electron transport process under the built-in electric field. It demonstrated a responsivity above 0.1 A W^−1^ under bias voltage, with a flat optical response across the 1450–1590 nm wavelength range.

Although graphene photodetectors based on conventional rectangular waveguides have demonstrated promising performance, their optical field distribution and light-matter interaction remain somewhat constrained. To further enhance photodetector performance, researchers have begun exploring waveguide structures with unique optical properties, such as slot waveguides^71^, photonic crystal waveguides^80^, and microring resonators^47^. These structures not only enhance light-field confinement and increase the light-graphene interaction but also optimize field distribution, leading to improved detection responsivity and response time. In 2016, Schuler et al. first developed a graphene photodetector integrated with a silicon slot waveguide^71^. Figure 6c illustrates the device’s schematic construction along with its frequency response. In this work, the slot waveguide served two key functions: first, the two silicon strips acted as local gates, enabling the creation of a tunable p-n junction in graphene without introducing additional radio-frequency (RF) capacitance that could affect the bandwidth. Second, the slot confined the optical field within a narrow region, enhancing its interaction with the graphene layer. This photodetector operated under zero-bias voltage based on the PTE mechanism, achieving a responsivity of 3.5 V W^−1^. Additionally, benefiting from the ultrafast relaxation of hot electrons, the device attained an impressive 3 dB bandwidth as high as 65 GHz. In recent years, Alaloul et al. employed similar structures for on-chip sensing in the MWIR range. The device featured miniaturization and high sensitivity, enabling sub-ppb-level detection of gases like carbon dioxide, methane, and nitrous oxide^209^. In 2018, Schuler et al. proposed an integration approach combining graphene with a silicon photonic crystal defect waveguide, where the line defect was formed by an air slot, as shown in Fig. 6d^80^. Similar to their previous work, the photonic crystal defect waveguide enhanced light-graphene interaction while functioning as an effective gate, enabling the creation of a tunable p–n junction within the light absorption region. Compared to traditional slot waveguides, the photonic crystal defect waveguide extended the control range of the gate, effectively improving the conversion efficiency of the electronic temperature gradient into photovoltage, thereby increasing the responsivity to 4.7 V W^−1^. Later, Schuler et al. once again introduced a method for integrating graphene with a silicon microring resonator, as depicted in Fig. 6e^47^. Benefiting from the high energy density within the microring resonator cavity, more than 90% of the light was absorbed with a graphene coverage length of only about 6 μm, increasing the intensity of the light–graphene interaction by about ten. This photodetector demonstrated a 3 dB bandwidth of 12 GHz and a peak responsivity of 90 V W^−1^.

Apart from the aforementioned waveguide structure, introducing a waveguide structure that can generate surface plasmon polaritons provides a promising strategy for strengthening light–matter interaction. Surface plasmon polaritons mainly refer to the fluctuation generated when the metal surface interacts with electromagnetic waves, and their energy is mainly concentrated near the metal-medium interface^210^. In addition, the fluctuation characteristics of surface plasmon polaritons can be modulated by engineering the shape of metals and the size of nanostructured metals^76^. Currently, waveguides that can generate surface plasmon polaritons can generally be classified into two categories: hybrid surface plasmon polariton waveguides and surface plasmon polariton slot waveguides^35^. Hybrid surface plasmon polariton waveguides mainly comprise nanostructured metal, 2D materials, and waveguides. The researchers induced surface plasmon polariton generation through the metal nanostructures on top of the waveguide and 2D materials to enhance the 2D materials’ ability to absorb light. Dating back to 2013, Pospischil et al. utilized metal strips as signal electrodes on top of silicon waveguides and graphene, doubling the total photocurrent using the surface plasmon polariton mode induced by the metal structure^37^. In 2016, Goykhman et al. reported a metal-graphene-silicon photodetector that exhibited high responsivity and an avalanche multiplication effect at the 1.55 μm telecommunication wavelength^211^. In this structure, the metal formed a Schottky contact with graphene, facilitating carrier injection into silicon, while also supporting surface plasmon polariton waveguide modes that confined the optical field at the Schottky interface, significantly enhancing light absorption in graphene. Under reverse bias, the device exhibited avalanche multiplication, leading to effective photocurrent amplification and achieving a responsivity of 0.37 A W^−1^. In 2018, Ma et al. introduced periodically arranged bowtie-shaped metal nanostructures on top of a silicon waveguide and graphene, which exhibited enhanced plasmonic fields at the edges and gaps of the bowtie structure, enabling the device to achieve a responsivity of 0.5 A W^-1^ and a bandwidth exceeding 110 GHz with a length of 6 μm^212^. As illustrated in Fig. 6f, Guo et al. transferred graphene onto a 100 nm-thick silicon ridge waveguide and exploited its weak mode field confinement in the perpendicular direction to enhance the material’s light absorption^45^. Figure 6g presents the mode field distribution of the device, revealing localized optical field enhancement near the broad metal strip. They chose a broader metal strip to decrease the metal-graphene contact resistance without introducing significant metal absorption loss. The structure simultaneously achieved low metal loss and high graphene optical absorption, achieving a responsivity of 70 mA W^-1^ with a 3 dB bandwidth surpassing 20 GHz at 2 μm and a responsivity of 400 mA W^−1^ alongside a 3 dB bandwidth over 40 GHz at 1.55 μm. In the most recent studies, Cai et al. demonstrated two different shapes of hybrid surface plasmon polariton waveguides with ultra-low optical loss at 2 μm wavelength and polarization-independent light detection^213^. They achieved ultra-low metal loss by employing a metal rod nanostructure with a period spacing of 150 nm, simultaneously increasing the light-graphene interaction intensity by a factor of three, as shown in Fig. 6h. Furthermore, they successfully realized polarization-independent light detection by employing a dual-metal-strip structure with a width of 150 nm and a spacing of 600 nm.

In the above work, hybrid surface plasmon polariton waveguides often confine the light field at the interface between the metal and the waveguide, which improves the strength of the interaction between 2D materials and optical waves. However, hybrid surface plasmon polariton waveguides also have some limitations, such as insufficient light-field confinement effect and higher contact resistance^35^. To address these issues, researchers proposed a surface plasmon polariton slot waveguide with enhanced light confinement capabilities, allowing the optical field to be concentrated within a smaller region. For example, in 2019, Ding et al. used a 120 nm wide narrow plasma slot structure to enhance both the photodetector’s responsivity and bandwidth^62^, as demonstrated in Fig. 6i. In this work, the light was coupled into the optical waveguide through a grating coupler and then directed into the surface plasmon polariton slot waveguide, which was composed of two asymmetric metal contacts via a short conical silicon waveguide structure. The 120 nm-wide slot significantly enhanced light confinement, thereby boosting the light absorption of graphene. Simultaneously, the ultra-short carrier transport path enhanced the photodetector’s response speed. Finally, the photodetector achieved a bandwidth surpassing 110 GHz and 360 mA W^−1^ responsivity. In 2022, Yan et al. proposed a novel dual-slot structure by cleverly combining a silicon slot waveguide with a metal surface plasmon polariton slot waveguide and realized a photodetector demonstrating both 603.92 mA W^−1^ responsivity and 78 GHz bandwidth by optimizing the structural parameters^73^.

Although certain specialized waveguides can effectively enhance light-graphene interaction due to their unique properties, they also have some limitations. For instance, slot waveguides can effectively confine the optical field within the slot, enhancing photoelectric conversion efficiency. However, due to the narrow slot width, edge roughness introduced during fabrication may lead to significant scattering loss, resulting in substantial optical attenuation^214^. Photonic crystal waveguides leverage Bragg reflection to confine the guided mode within crystal defects, but their fabrication process is complex and costly^215^. Microring resonators can achieve high optical field density through resonance effects, but their effectiveness is limited to a narrow wavelength range^216^. Meanwhile, metallic nanostructures can facilitate the generation of surface plasmon polaritons, yet their considerable metal losses remain a critical drawback^217^. Therefore, besides optimizing the waveguide structure, the electrode, an indispensable component of photodetectors, offers a viable and efficient strategy for improving device performance through design optimization. A well-designed electrode configuration not only enhances carrier collection efficiency and reduces contact resistance but also minimizes parasitic capacitance effects, further enhancing the photodetector’s comprehensive performance^218^. In 2014, Schall et al. achieved a silicon waveguide photodetector built on wafer-scale CVD-grown graphene, where the electrode contact spacing was controlled at 1.4 μm. This design shortened the carrier transport path, enabling a 3 dB bandwidth as high as 41 GHz at a wavelength of 1550 nm^219^. Since bandwidth is influenced by device resistance and capacitance, in 2017, Schall et al. further minimized the area of the contact pads, reducing capacitance and increasing bandwidth to 76 GHz^61^. Lastly, Guo et al. developed a graphene-silicon heterostructure photodetector operating under IPE and photo-assisted thermionic field emission mechanisms, incorporating a metal-graphene-metal sandwiched contact structure, as illustrated in Fig. 6j^66^. In this construction, the spacing between the graphene layers was controlled at 300 nm, making sure that carriers were mainly transported within the graphene layer. Additionally, by sandwiching the graphene between metal electrodes, the metal electrodes were prevented from directly contacting the silicon waveguide, reducing contact resistance and consequently enhancing the photodetector’s bandwidth to 30 GHz.

Furthermore, gate modulation significantly improves photodetector performance. For instance, in photodetectors operating under the PTE mechanism, the gate enables tuning of the Fermi level, optimizing the term voltage generated by the temperature gradient and thereby increasing the thermally driven photocurrent^220^. In photodetectors based on the PVE mechanism, the gate can adjust the p-n junction, improving the division rate of photogenerated carriers and enhancing the built-in electric field to boost photoelectric conversion efficiency^221^. In both mechanisms, gate control effectively reduces dark current and increases the signal-to-noise ratio, leading to superior photodetector performance^149^. In 2015, Shiue et al. spin-coated a polymer electrolyte across the entire chip to serve as a top gate for modulating the Fermi level of graphene, enabling independent control of the carrier concentration in graphene and optimizing the photoelectric response^222^. Under the combined influence of the gate-source voltage and drain-source voltage, the photodetector exhibited the typical working mechanism of PTE, significantly enhancing the photocurrent generation efficiency. It was experimentally observed that the photodetector achieved a responsivity of 0.36 A W^−1^ and a 3 dB bandwidth of 42 GHz, representing an improvement of more than three times compared to previously reported waveguide-integrated graphene photodetectors. In 2021, Marconi et al. implemented a split-gate electrostatic modulation structure where the upper graphene layer electrostatically induced a step change in the Seebeck coefficient of the lower graphene layer, as shown in Fig. 6k^48^. To electrically isolate the two graphene layers, they introduced a low-resistance silicon nitride layer, enabling impedance matching with the 50 Ω test equipment. The resulting photodetector exhibited a responsivity of 3.5 V W^−1^ and achieved a 3 dB bandwidth of approximately 70 GHz.

Apart from the aforementioned approaches, researchers can further improve photodetectors’ performance by changing the substrate. Restricted by the buried oxide layer’s high absorption, the operating wavelength of conventional silicon-on-insulator platforms is typically restricted to 4 µm. In contrast, calcium fluoride (CaF_2_), a halide with a transparent window extending to 8 µm with a refractive index as low as 1.4, offers new possibilities for extending the operating wavelength of on-chip photodetectors. In 2021, Ma et al. proposed a superior efficiency transfer printing approach for the flexible integration of waveguide and substrate materials^223^. They heterogeneously integrated a silicon waveguide on a CaF_2_ substrate and fabricated the resulting Si-on-CaF_2_ waveguide with low attenuation in a wide LWIR wavelength range of 6.3–7.1 μm. Additionally, they achieved plasmonic enhancement through a hybrid surface plasmon polariton waveguide structure, ultimately enabling the photodetector to attain a responsivity of approximately 8 mA W^−1^.

Optimizing infrared 2D material structure is also an effective approach to enhancing photodetector performance, yet a recent study explored an alternative strategy by tuning the structural properties of graphene to achieve further optimization. In 2024, Wu et al. showed a waveguide-integrated twisted bilayer graphene (tBLG) photodetector, as displayed in Fig. 6l^55^. By designing the twist angle of graphene to be 4.1°, the researchers aligned the energy difference between the van Hove singularities in the tBLG band structure with the photon energy of the 1550 nm telecommunication wavelength, significantly enhancing light coupling efficiency. Additionally, the nearly linear band dispersion of tBLG near the Dirac point enabled it to maintain ultrahigh carrier mobility comparable to that of monolayer graphene, ensuring excellent high-frequency performance. Simulation results indicated that tBLG exhibited approximately three times the coupling efficiency of monolayer graphene, allowing for a shorter channel length and improved responsivity. Finally, the device achieved a responsivity of 0.65 A W^−1^ at 1550 nm and a 3 dB bandwidth exceeding 65 GHz.

Despite the impressive broadband response and high-speed performance achieved by the aforementioned silicon waveguide-integrated graphene photodetectors through waveguide and material engineering, the zero-bandgap nature of graphene generally leads to relatively large dark currents^213^. This increases static power consumption and raises the noise level, which in turn limits further improvements in signal-to-noise ratio and detectivity. This issue is not unique to graphene, as for infrared operation, certain narrow-bandgap 2D semiconductors can also exhibit increased thermally generated dark currents, especially when their bandgaps are engineered toward longer wavelengths. As a result, achieving effective dark-current suppression while preserving high responsivity and large bandwidth has become one of the key challenges on the path toward practical waveguide-integrated 2D-material infrared photodetectors. In response to this challenge, a variety of device concepts and engineering strategies have recently been proposed, and their representative advances and underlying mechanisms are systematically discussed in Section 7.

#### BP

**Fig. 7:**
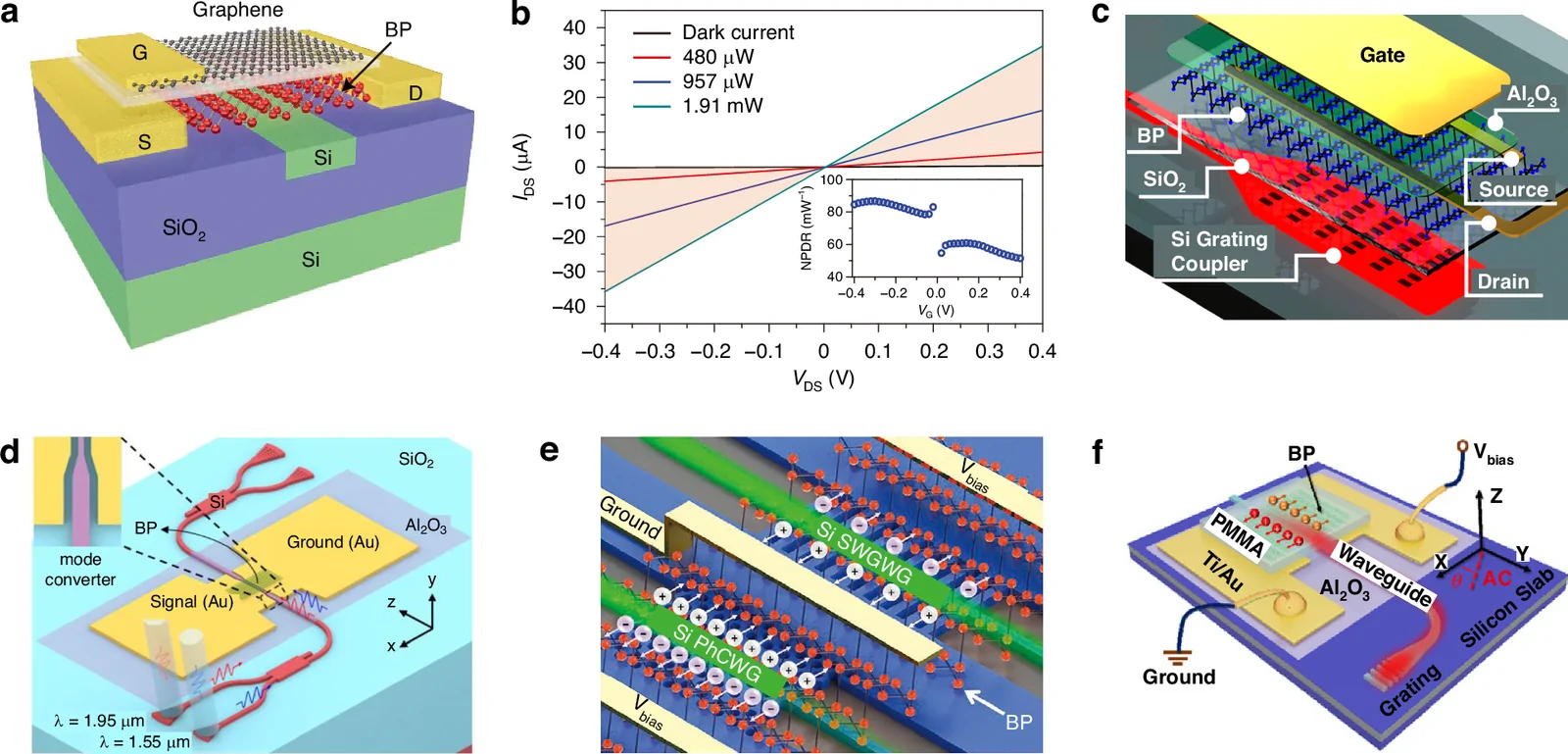
BP infrared photodetectors based on silicon waveguides. **a** Architecture of a BP photodetector coupled with a rectangular silicon waveguide^40^. Reproduced from ref. ^40^ with permission of Springer Nature. **b** The BP photodetector’s photoresponse curves with various bias voltages. The inset shows its NPDR^40^. Reproduced from ref. ^40^ with permission of Springer Nature. **c** BP photodetector structure employing a silicon waveguide and a grating coupler^225^. Reproduced from ref. ^225^ with permission of Copyright © 2018 American Chemical Society. **d** BP photodetector structure employing a silicon waveguide combined with a surface plasmon polariton slot^78^. Reproduced from ref. ^78^ with permission of Copyright © 2022 American Chemical Society. **e** Design layout of a BP photodetector employing a photonic crystal waveguide in conjunction with a subwavelength grating waveguide^83^. Reproduced from ref. ^83^ with permission of John Wiley and Sons. **f** The structure of a BP photodetector encapsulated with PMMA for environmental protection^229^. Reproduced from ref. ^229^ with permission of John Wiley and Sons

Although graphene’s zero-bandgap characteristic enables broadband photodetection, it also introduces a significant dark current, which can not be overlooked, particularly in photodetectors operating based on the PCE mechanism. In contrast, BP, with its tunable bandgap, the wide range of visible to mid-infrared light spectral response, and carrier mobility of roughly 1000 cm^2 ^V^−1^ s^−1^, stands out as a viable candidate for broadband photodetection applications^224^. In 2015, Youngblood et al. first demonstrated a silicon waveguide-integrated BP photodetector with a maximum responsivity of 657 mA W^−1^ and a response bandwidth of more than 3 GHz^40^, as depicted in Fig. 7a. The photodetector utilized the top graphene layers as a tunable gate electrode, enabling control over the carrier type and concentration within the BP channel. Remarkably, when operated at an optimized bias voltage of −0.4 V, the photodetector demonstrated an exceptionally low dark current of merely 220 nA, which was more than three orders of magnitude lower than that of previously reported graphene photodetectors under similar conditions. Figure 7b presents the current response of the photodetector under various source-drain bias voltages, while the inset shows the normalized photo-to-dark current ratio (NPDR), which is four orders of magnitude higher than typical graphene-based devices^37^.

Although the minimum theoretical bandgap of BP is roughly 0.3 eV, equivalent to a 4.13 μm cutoff wavelength, its light absorption and responsivity decrease significantly when the wavelength exceeds 3 μm and approaches the cutoff. In 2019, Huang et al. proposed a design that integrated a silicon waveguide with a grating structure to address the limited light absorption of BP near its band edge, as shown in Fig. 7c^225^. The waveguide structure enabled potential integration with other photonic components, while the grating converted the in-plane guided mode into an out-of-plane coupled wave, thereby significantly enhancing the interaction between light and BP layer. Furthermore, they optimized the orientation of the BP crystal to align with both the carrier transport direction and the polarization of the waveguide optical mode. By tuning the drain and gate biases, they modulated the carrier concentration, hole barrier height, and the involvement of trap-assisted photoconductive gain mechanisms, which helped reduce the dark current and boost the responsivity. Consequently, the device achieved excellent responsivities of 23 A W^−1^ at 3.68 μm and 2 A W^−1^ at 4 μm. Similarly, Liu et al. developed a high-efficiency BP avalanche photodetector founded on a surface plasmon polariton slot and silicon waveguide, as depicted in Fig. 7d^78^. In this design, a 200 nm-wide metallic slot served a dual purpose: enhancing optical field localization and serving as the source and drain electrodes to shorten the carrier transport length, thereby improving the bandwidth of the photodetector. Under high-bias voltage, the device exhibited nonlinear enhancement in photocurrent, suggesting the involvement of an avalanche multiplication mechanism. Benefiting from this mechanism, the device achieved ultrahigh responsivities of 66 and 125 A W^−1^ at 1.55 and 1.95 μm, respectively, while maintaining a bandwidth of up to 1.05 GHz.

Gratings and metallic nanostructures have been shown as efficient approaches to increasing the responsivity of BP photodetectors. However, the relatively large size of grating structures poses challenges for device miniaturization, while the absorption loss associated with metallic nanostructures remains a critical issue^42^. To address these limitations, photonic crystal waveguides offer a promising alternative by leveraging their unique slow-light effect to improve BP photodetector performance. In photonic crystal waveguides, the photonic bandgap effect leads to a flattening of the dispersion curve near the band edge, significantly reducing the group velocity of light. Additionally, multiple reflections and interference within the structure, induced by Bragg reflection, further slow down the phase advancement of the optical wave, thereby enhancing the slow-light effect. This extreme reduction in group velocity compresses the optical energy spatially, substantially strengthening light–matter interactions. In 2020, Ma et al. presented a strategy to enhance BP photodetector responsivity through the slow-light effect in photonic crystal waveguides^83^. To investigate its impact, they designed two waveguide structures of equal length on the same chip for comparative analysis: one with the slow-light effect (a photonic crystal waveguide) and the other without it (a subwavelength grating waveguide), as displayed in Fig. 7e. Experimental results showed that at the band edge of 3.8 μm, the 10 μm-long photonic crystal waveguide improved the responsivity of the BP photodetector by an order of magnitude compared to the subwavelength grating waveguide. Under a 0.5 V bias voltage, the photodetector achieved a responsivity of 11.31 A W^−1^, validating the role of the slow-light effect in improving photodetector performance. Unlike the previous work, Tian et al. incorporated two air holes into a photonic crystal waveguide to form a 15 μm-long cavity, enabling multiple reflections of light within the cavity and further extending the light–matter interaction length^82^. As a result, under a 0.5 V bias voltage, the device exhibited a dark current of only 20 nA, a responsivity of 125 mA W^−1^, and a 3 dB bandwidth surpassing 1.42 GHz.

Unfortunately, owing to the presence of chemically active lone electron pairs on the surface, BP is intrinsically vulnerable to environmental degradation^226^. Previous studies have shown that BP readily undergoes oxidation upon exposure to oxygen, while moisture plays a critical catalytic role by interacting with oxidized surfaces and accelerating the breaking of P-P bonds^227^. Moreover, optical illumination can further promote photo-assisted oxidation processes, and degradation is often initiated at defect sites and edges, making few-layer BP particularly susceptible under ambient operating conditions^228^. As a result, the stability of BP-based photodetectors remains a major concern for practical applications. To overcome this challenge, in 2019, Yin et al. spin-coated a 500 nm-thick poly(methyl methacrylate) (PMMA) layer onto the chip, introducing an upper cladding that effectively isolated BP from oxygen and moisture^229^, as shown in Fig. 7f. Experimental results demonstrated that the PMMA encapsulation had a negligible impact on the 3 dB bandwidth and responsivity, while significantly enhancing device robustness, enabling stable operation even after six months of use. Apart from the PMMA-based organic encapsulation scheme demonstrated on waveguide-integrated BP devices, a variety of other material-level passivation strategies have been developed for BP devices, including dielectric capping layers^230^, surface-oxidation-derived protective layers^231^, and van der Waals heterostructures^232^. Although most of these approaches have so far been investigated in non-waveguide-integrated configurations, they could in principle be adapted to waveguide platforms. These methods provide complementary routes for improving the long-term environmental stability of BP and offer valuable guidance for the design of robust BP-based infrared photodetectors.

#### TMDs

Although BP photodetectors exhibit performance comparable to graphene-based photodetectors and offer advantages in dark current suppression, their environmental instability remains a critical factor limiting their development. Alternatively, TMDs have been identified as strong candidates to succeed graphene, primarily owing to their intrinsic bandgap typically spanning the visible to near-infrared range, which effectively mitigates the zero-bandgap issue of graphene while enabling higher on/off ratios and lower dark currents. Moreover, TMDs possess strong light absorption capabilities, bandgap tunability through layer number modulation, and outstanding stability, exhibiting exceptional suitability for optoelectronic detection applications^233^. Among the TMD family, 2H-MoTe_2_ stands out since its tunable electronic behavior, exceptional mechanical flexibility, and excellent optical response characteristics. Additionally, its narrow bandgap (ranging from 0.93 to 1.05 eV) exhibits layer-dependent behavior, demonstrating exceptional potential for near-infrared photodetection^234^.

**Fig. 8:**
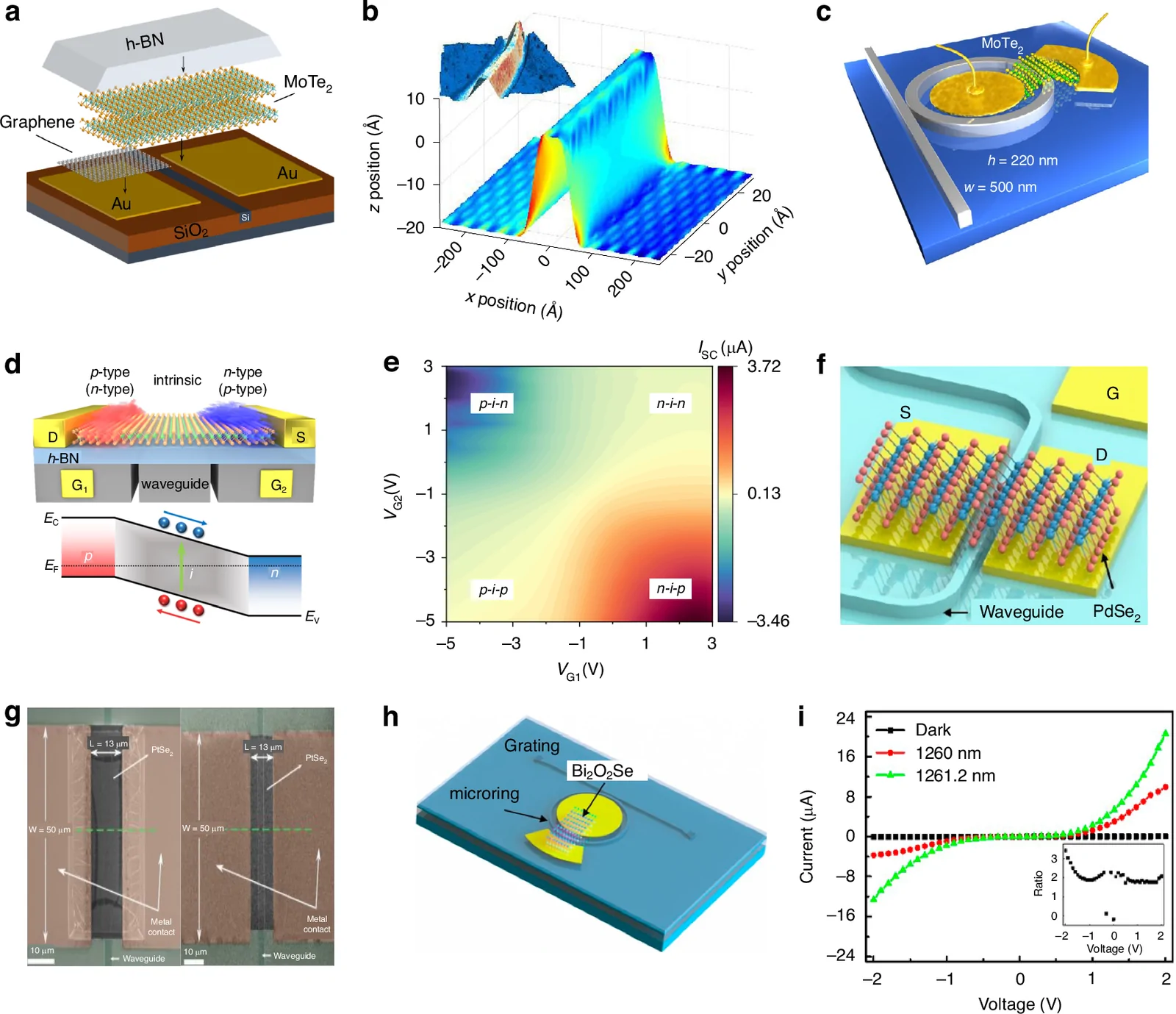
Silicon waveguide-integrated infrared photodetectors based on TMDs, noble metal dichalcogenides, and other emerging 2D materials. **a** The structure of a MoTe_2_ photodetector integrated with the rectangular silicon waveguide^235^. Reproduced from ref. ^235^ under (CC BY 4.0). **b** Simulated strain distribution in MoTe_2_ under a non-planar waveguide configuration^43^. Reproduced from ref. ^43^ with permission of Springer Nature. **c** The structure of a strain-engineered MoTe_2_ photodetector integrated with the silicon microring resonator^43^. Reproduced from ref. ^43^ with permission of Springer Nature. **d** MoTe_2_ photodetector structure incorporating a p-i-n homojunction and a photonic crystal waveguide^49^. Reproduced from ref. ^49^ with permission of Copyright © 2022 American Chemical Society. **e** Photoresponse of a MoTe_2_ photodetector under gate modulation^49^. Reproduced from ref. ^49^ with permission of Copyright © 2022 American Chemical Society. **f** The structure of a PdSe_2_ photodetector integrated with the silicon waveguide^237^. Reproduced from ref. ^237^ with permission of Copyright © 2022 American Chemical Society. **g** Scanning electron microscope image (false-colored) of a PtSe_2_ photodetector integrated onto a silicon waveguide through transfer and direct growth approaches^239^. Reproduced from ref. ^239^ under (CC BY-NC-ND 4.0). **h** The structure of a Bi_2_O_2_Se photodetector integrated with the silicon microring resonator^168^. Reproduced from ref. ^168^ with permission of Copyright © 2021 American Chemical Society. **i** Photocurrent responses of the device at resonant and non-resonant wavelengths^168^. Reproduced from ref. ^168^ with permission of Copyright © 2021 American Chemical Society

In 2018, Ma et al. successfully fabricated a silicon waveguide-integrated MoTe_2_ photodetector with a responsivity of 23 mA W^−1^ and a frequency response exceeding 500 MHz, as illustrated in Fig. 8a^235^. Notably, they introduced graphene as an interfacial layer between MoTe_2_ and the metal electrode on one side. This structure was designed because the Fermi level of graphene was closer to the valence band of MoTe_2_ than metals, thus forming asymmetric Schottky junctions on both sides. This asymmetry could not only suppress carrier injection into MoTe_2_ and hence enable ultralow dark current in the nA range but also generate a built-in electric field that facilitates carrier segregation at zero external bias. Moreover, this design eliminated the need for an externally integrated gate, simplifying the fabrication process while avoiding parasitic absorption losses from gate metal electrodes, which would otherwise degrade quantum efficiency.

However, since the telecommunication wavelength of 1550 nm is near the absorption band edge of MoTe_2_, MoTe_2_-based photodetectors exhibit relatively low responsivity at this wavelength, limiting their further development. To deal with this problem, in 2020, Maiti et al. presented a strain-engineering method to modulate the bandgap of MoTe_2_ by depositing it onto a non-planar waveguide^43^. Owing to the van der Waals attraction between the suspended monolayer MoTe_2_ and the substrate, localized strain was induced in specific regions, as illustrated in Fig. 8b. Both experimental and simulation results demonstrated that tensile strain could reduce the MoTe_2_ bandgap from 1.06 eV to approximately 0.8 eV, enabling effective optical response at the 1550 nm telecommunication wavelength. Moreover, to further reduce device footprint and enhance light-matter interaction, the authors heterogeneously integrated multilayer MoTe_2_ onto a silicon microring resonator, as illustrated in Fig. 8c. The high optical field energy density of the microring resonator structure further enhanced MoTe_2_ absorption at 1550 nm. With −2 V applied bias, the photodetector exhibited exceptional performance metrics, including a peak responsivity of 0.5 A W^−1^, a low dark current of 13 nA, and a response frequency extending to 35 MHz. This study highlights strain engineering as a new degree of freedom for tuning the properties of 2D materials and provides an alternative strategy for extending the applications of MoTe_2_ in optical communication.

To achieve both high responsivity and large bandwidth in MoTe_2_-based photodetectors, in 2022, Li et al. proposed an infrared photodetector employing the MoTe_2_ p-i-n homojunction integrated with a silicon photonic crystal waveguide^49^, as shown in Fig. 8d. The photonic crystal waveguide could not only utilize Bragg reflection to constrain the guided mode within the central line defect and extend the propagation path of light within MoTe_2_ and enhance light–matter interaction, but also make contact with the electrodes and hence form two independent gates that enabled a reconfigurable MoTe_2_ p-i-n homojunction (Fig. 8e). The intrinsic region (i-region) could also effectively reduce junction capacitance, increase the operational frequency while suppressing the off-state current, lower power consumption, and expand the optically active area to enhance photoelectric conversion efficiency. Thus, the photodetector displayed a responsivity of 0.4 A W^−1^ and a calculated dynamic response bandwidth of 34 GHz.

#### Noble metal dichalcogenides

Although numerous methods have been presented to enhance the infrared absorption of MoTe_2_ photodetectors, the intrinsic bandgap of MoTe_2_ still limits further applications beyond the MWIR range. In recent years, noble metal dichalcogenides like PdSe_2_ and PtSe_2_ have received growing attention because of their superior air stability and tunable bandgaps, as well as their smaller bandgaps compared to MoTe_2_. For instance, the bandgaps of PdSe_2_ and PtSe_2_ can be as low as 0.03 eV, covering the spectral range from near-infrared to mid-infrared, making them promising candidates for infrared photodetection^164,236^. In 2022, Wu et al. integrated PdSe_2_ with a silicon waveguide for on-chip photodetection, which exhibited great responsivity in the 1260 to 1565 nm band, up to 1758.7 mA W^−1^, as well as a low noise equivalent power of 4.0 pW Hz^−0.5^ and a response bandwidth of 1.5 GHz (Fig. 8f)^237^. To increase the detection efficiency of PdSe_2_ photodetectors, in 2023, Jian et al. integrated them with a surface plasmon polariton slot waveguide, leveraging its short carrier transport path and optical field localization enhancement effect to achieve a response bandwidth of 17.5 GHz^238^. Additionally, Gong et al. recently performed a comprehensive investigation on the impact of electrode spacing on the capability of PdSe_2_ photodetectors^218^. By analyzing devices integrated with rectangular waveguides, ridge waveguides, and slot waveguides, they theoretically determined the optimal electrode positions for different waveguide configurations. Simulation results showed that under a 5 V bias voltage, the optimized device achieved a peak responsivity of 9.24 A W^−1^ at 1550 nm, representing a 481% improvement compared to devices with unoptimized electrode positioning. Apart from PdSe_2_, Parhizkar et al. recently fabricated infrared detectors by integrating PtSe_2_ with silicon waveguides^239^. In their work, they compared the effects of direct growth integration and wet transfer technology on device performance, as displayed in Fig. 8g. The experimental results demonstrated that the wet transfer process could introduce a gas gap at the material-waveguide interface, leading to undesirable structural folds. In contrast, the direct growth integration method demonstrated a perfect fit between PtSe_2_ and silicon waveguide without wrinkles and hence presented superior device performance, including a responsivity of 11 mA W^−1^ and a rapid response time below 8.4 μs.

#### Other emerging 2D materials

Besides the aforementioned 2D materials that have demonstrated successful integration in waveguide-based photodetectors, emerging 2D materials with exceptional properties are gaining significant research interest. Particularly, Te and Bi_2_O_2_Se have surfaced as attractive options due to their remarkable characteristics, including environmental stability, narrow bandgap, and great carrier mobility. Bi_2_O_2_Se is a 2D material that remains stable in air and possesses an indirect bandgap of approximately 0.8 eV, along with an excellent carrier mobility of roughly 29,000 cm^2 ^V^-1^ s^−1^, offering distinct advantages for NIR photodetection^240^. In 2021, Wu et al. employed a polycarbonate/polydimethylsiloxane-assisted transfer technique to reliably transfer high-quality Bi_2_O_2_Se onto a silicon-based microring resonator, enabling the device to achieve a responsivity of 3.5 A W^−1^ at a wavelength of 1260 nm, as displayed in Fig. 8h^168^. The microring resonator structure not only allowed the device to maintain photoelectric conversion efficiency comparable to that of straight-waveguide photodetectors with longer and thicker Bi_2_O_2_Se layers but also effectively suppressed the dark current. Further analysis revealed that the photocurrent response at the resonant wavelength was enhanced by approximately three times compared to off-resonance conditions, highlighting the device’s strong potential for wavelength-selective on-chip spectroscopy and sensing applications (Fig. 8i). Te is another air-stable 2D material that features a thickness-dependent tunable bandgap and can be produced via inexpensive solution-based methods, enabling large-area and high-quality material fabrication^170^. In 2022, Ma et al. produced high-quality Te nanoplates by an inexpensive hydrothermal method^241^. They integrated them with silicon waveguides and combined the synergistic effect of the carrier collection path and crystal orientation with the highest carrier mobility of Te, which finally resulted in high-performance photodetectors with an excellent responsivity of 2.3 A W^−1^ in the 2015 nm band and a wide 3 dB bandwidth of 4 GHz.

#### 2D Material heterostructures

Although waveguide-integrated photodetectors based on single 2D materials have exhibited exceptional optoelectronic characteristics, they still face inherent limitations. For example, zero-bandgap materials such as graphene exhibit ultrafast response but suffer from weak light absorption and high dark current^242^. In contrast, intrinsic-bandgap TMDs and noble metal dichalcogenides like MoTe_2_ and PdSe_2_ offer higher light absorption and on/off ratio but have lower carrier mobility, which restricts device response speed^243^. Additionally, the spectral response of a single material is typically constrained by its bandgap, making it challenging to achieve broadband photodetection. To address these challenges, researchers have begun exploring heterostructure photodetectors that integrate multiple different 2D materials, which not only combine the benefits of different materials, like high mobility, strong optical absorption, and tunable bandgaps, but also optimize interfacial properties, enhance carrier transport, and improve photoelectric conversion efficiency^244^. Moreover, from a fabrication perspective, heterostructures can be realized through van der Waals stacking or transfer techniques, ensuring compatibility with existing silicon photonics technology^245,246^.

**Fig. 9:**
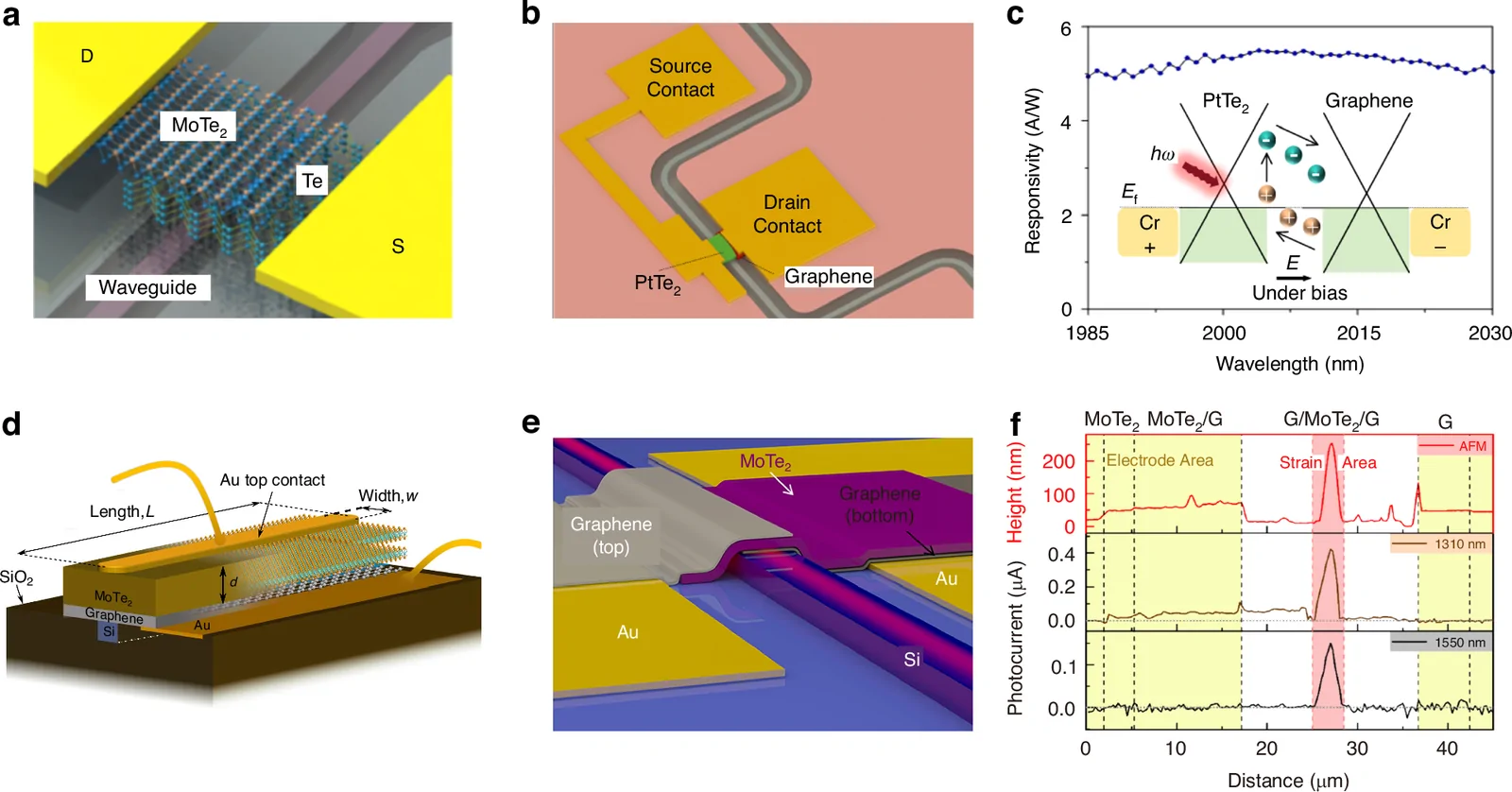
2D material heterojunction infrared photodetectors based on silicon waveguides. **a** The structure of a Te-MoTe_2_ heterojunction photodetector^247^. Reproduced from ref. ^247^ with permission of Copyright © 2024 American Chemical Society. **b** Schematic layout of the PtTe_2_-graphene heterojunction photodetector^54^. Reproduced from ref. ^54^ with permission of Copyright © 2024 American Chemical Society. **c** Responsivity characteristics and schematic of the operating principle of the PtTe_2_-graphene heterojunction photodetector^54^. Reproduced from ref. ^54^ with permission of Copyright © 2024 American Chemical Society. **d** The structure of a MoTe_2_-graphene vertical heterojunction photodetector^44^. Reproduced from ref. ^44^ with permission of Springer Nature. **e** The structure of a strain-engineered MoTe_2_-graphene vertical heterojunction photodetector^248^. Reproduced from ref. ^248^ with permission of Copyright © 2025 American Chemical Society. **f** Photocurrent responses from different regions of the MoTe_2_-graphene vertical heterojunction photodetector under 1310 and 1550 nm illumination^248^. Reproduced from ref. ^248^ with permission of Copyright © 2025 American Chemical Society

Ma et al. fabricated high-performance heterojunction infrared photodetectors operating beyond the telecommunication band by first integrating Te-MoTe_2_ p–n heterostructure and silicon waveguides (Fig. 9a)^247^. Benefiting from the depletion layer at the interface, the diffusion of the majority of carriers was effectively suppressed, resulting in a reduced dark current and a high detectivity of 2 × 10^9 ^cm Hz^1/2^ W^−1^. Moreover, due to stronger optical density within silicon waveguides, a confined pathway for capturing photoinduced charges, and large charge mobility of Te, the photodetector also displayed an excellent responsivity and bandwidth of 94.16 mA W^-1^ and 36 MHz, respectively. More recently, Ma et al. combined silicon waveguides with single PtTe_2_ (utilize a Mach-Zehnder interferometer) and PtTe_2_-graphene heterostructure (utilize a bending waveguide) to construct on-chip infrared photodetectors (Fig. 9b), which mainly generated photoinduced charges and photocurrents based on the PCE (Fig. 9c)^54^. By comparison, it was found that the PtTe_2_-graphene heterojunction-based device could present much higher responsivity, bandwidth, and EQE of around 5.5 A W^−1^, 35 MHz, and 119% under 2 μm light illumination because graphene has a high carrier mobility.

Compared to the aforementioned lateral heterostructure photodetectors, vertical heterojunction structures offer a shorter carrier transport path, significantly enhancing carrier separation and collection efficiency and thereby enabling faster response time. In 2020, Flöry et al. fabricated a MoTe_2_-graphene vertical heterostructure on a silicon waveguide, as illustrated in Fig. 9d^44^. This photodetector combined the significant optical absorption of MoTe_2_ with the high carrier mobility of graphene, utilizing a vertical heterostructure to effectively reduce the transport path of photogenerated carriers. Additionally, the design of narrow metal contacts optimized the device’s RC time constant, resulting in an ultrahigh response bandwidth of 24 GHz. Further improvements, such as increasing the bias voltage or using thinner MoTe_2_ layers, could potentially extend the bandwidth to 50 GHz. Most recently, Yang et al. presented a strain-engineered MoTe_2_-graphene vertical heterojunction photodetector, as shown in Fig. 9e^248^. By introducing a rectangular waveguide structure, the device leveraged the van der Waals attraction between the suspended region of MoTe_2_ and the substrate to induce localized strain, which in turn modulated the material’s bandgap and extended its operational wavelength to 1550 nm. As displayed in Fig. 9f, the device’s photocurrent characteristics were characterized under 1310 and 1550 nm laser illumination. The results showed that the photodetector exhibited a weak response at 1310 nm in the unstrained region and almost no photocurrent at 1550 nm. In contrast, the strained region demonstrated a clear photocurrent response at both wavelengths, further validating the effectiveness of strain engineering in modulating the MoTe_2_ bandgap and broadening its spectral response range. Furthermore, multilayer graphene, acting as a transparent electrode, encapsulated the ultrathin MoTe_2_ layer. Under an applied vertical electric field, this configuration effectively promoted the separation and extraction of electron-hole pairs, reducing the carrier transport path and enhancing the response speed. As a result, the photodetector achieved 3 dB bandwidths of 4.81 GHz at 1310 nm and 3.85 GHz at 1550 nm, setting a new record for the broadband performance of MoTe_2_-based photodetectors in the telecommunication band. These findings highlight that constructing 2D material heterostructures provides a broader design space and greater potential for performance optimization in photodetectors, making it a key direction for the creation of next-generation high-efficiency infrared photodetection technologies. For comparison, Table 3 lists the structures and important performance metrics of the silicon waveguide-integrated 2D-material photodetectors discussed in this section.

**Table 3:** Structures and key properties of silicon waveguide-integrated 2D-material infrared photodetectors

Waveguide			2D material Performance						Ref.
Material	Structure		Mechanism	Wavelength	Responsivity	Bandwidth	Dark current	NEP or D*	
Silicon	Hybrid SPP	Graphene	PVE/PTE	1.31–1.65 μm	30 mA W^−1^ (0 V)	~18 GHz	—	—	^37^
	Ridge	Graphene	IPE	2.75 μm	0.13 A W^−1^ (1.5 V)	4 kHz	—	—	^38^
	Rectangular	Graphene	PVE	1.45–1.59 μm	0.108 A W^−1^ (1 V)	>20 GHz	—	—	^39^
	Slot	Graphene	PTE/PCE/BOL	1.55 μm	3.5 V W^−1^	65 GHz	—	—	^71^
	PhC	Graphene	PTE/PCE	1.55 μm	4.7 V W^−1^	>18 GHz	—	—	^80^
	MRR	Graphene	PTE	1.55 μm	90 V W^−1^	~12 GHz	—	—	^47^
	Hybrid SPP	Graphene	IPE	1.55 μm	85 mA W^−1^ (1 V)	—	~ 20 nA	—	^211^
	Hybrid SPP	Graphene	BOL	1.48–1.62 μm	0.5 A W^−1^ (0.4 V)	>110 GHz	—	—	^212^
	Hybrid SPP	Graphene	PTE/BOL/PCE	1.55 μm	0.4 A W^−1^ (0.3 V)	>40 GHz	Few mA	4.86 pW Hz^−1/2^	^45^
				2 μm	70 mA W^−1^ (0.3 V)	>20 GHz	Few mA	61.7 pW Hz^−1/2^	
	Hybrid SPP	Graphene	BOL	2 μm	122 mA W^−1^ (0.26 V)	>14 GHz	Few mA	—	^213^
	SPP slot	Graphene	PVE	1.54 μm	360 mA W^−1^ (2.2 V)	>110 GHz	—	—	^62^
	Slot & SPP slot	Graphene	BOL	1.55 μm	603 mA W^−1^ (1.9 V)	78 GHz	—	—	^73^
	Rectangular	Graphene	PVE	1.55 μm	16 mA W^−1^ (0 V)	41 GHz	—	—	^219^
	Rectangular	Graphene	BOL	1.55 μm	1 mA W^−1^ (1 V)	>76 GHz	—	—	^61^
	Ridge	Graphene	IPE	1.55 μm	0.15 mA W^−1^ (6 V)	~30 GHz	<100 pA	—	^66^
				1.96 μm	0.015 mA W^−1^ (0 V)				
	Rectangular	Graphene	PTE/PVE	1.5–1.8 μm	0.36 A W^−1^ (1.2 V)	42 GHz	—	—	^222^
	Rectangular	Graphene	PTE	1.55 μm	3.5 V W^−1^	65 GHz	—	—	^48^
	Hybrid SPP	Graphene	PVE/PTE	6.3–7.1 μm	8 mA W^−1^ (0 V)	>10 kHz	—	2.3 nW Hz^−1/2^	^223^
	Hybrid SPP	Graphene	PCE	1.55 μm	0.65 A W^−1^ (0.5 V)	>65 GHz	—	—	^55^
	PhC	Graphene	PTE	1.56–1.63 μm	193 mA W^−1^(0 V)	17 GHz	—	—	^249^
	Rectangular	BP	PVE/BOL	1.55 μm	135 mA W^−1^ (0.4 V)	>3 GHz	220 nA	—	^40^
	Grating	BP	PGE	3.68–4.03 μm	23 A W^−1^ (1 V)	—	Few μA	<1 nW Hz^−1/2^	^225^
	Grating & SPP slot	BP	PCE/PGE	1.55 μm	10 A W^−1^ (1.5 V)	150 MHz	Few μA	—	^42^
	Rectangular & SPP slot	BP	PVE	1.55 μm	66 A W^−1^ (1 V)	1.05 GHz	<1 mA	—	^78^
				1.95 μm	125 A W^−1^ (1 V)				
	PhC	BP	PGE	3.825 μm	11.31 A W^−1^ (0.5 V)	1 GHz	<1.06 μA	0.012 nW Hz^−1/2^	^83^
	PhC	BP	PVE	1.55 μm	125 mA W^−1^ (0.5 V)	1.42 GHz	<20 nA	—	^82^
	Ridge	BP	PVE	2 μm	306 mA W^−1^ (0.4 V)	1.33 GHz	Few μA	—	^229^
	Rectangular	MoTe_2_	PVE/PGE	1.31 μm	23 mA W^−1^ (3 V)	>500 MHz	<20 nA	—	^235^
	MRR	MoTe_2_	PCE	1.55 μm	0.5 A W^-1^ (2 V)	~35 MHz	13 nA	90 pW Hz^−1/2^	^43^
	PhC	MoTe_2_	PVE	1.26–1.34 μm	0.4 A W^−1^ (1 V)	34 GHz	<90 pA	—	^49^
						Calculated			
	Ridge	PdSe_2_	PVE	1.26–1.56 μm	1.76 A W^−1^ (5 V)	1.5 GHz	38 μA	4 pW Hz^−1/2^	^237^
	SPP slot	PdSe_2_	PCE	1.55 μm	560.1 mA W^−1^ (3 V)	17.5 GHz	4.5 μA	17.9 pW Hz^−1/2^	^238^
	Rectangular	PdSe_2_	—	1.55 μm	9.24 A W^−1^ (5 V)	23.1 GHz	Few μA	—	^218^
					Simulated	Simulated	Simulated		
	Ridge	PdSe_2_	—	1.55 μm	3.34 A W^−1^ (5 V)	14.2 GHz	Few μA	—	
					Simulated	Simulated	Simulated		
	Slot	PdSe_2_	—	1.55 μm	4.52 A W^−1^ (5 V)	14.9 GHz	Few μA	—	
					Simulated	Simulated	Simulated		
	Ridge	PtSe_2_	—	1.55 μm	11 mA W^−1^ (0 V)	—	—	—	^239^
	MRR	Bi_2_O_2_Se	PCE	1.26–1.31 μm	3.5 A W^−1^ (2 V)	—	15.3 nA	15.1 pW Hz^−1/2^	^168^
	Rectangular	Te	PCE	2.015 μm	2.3 A W^−1^ (1.5 V)	4 GHz	Few μA	—	^241^
	Ridge	Te-MoTe_2_	PVE/PGE	2 μm	94.16 mA W^−1^ (0 V)	36 MHz	<1 nA	1.76 pW Hz^−1/2^	^247^
	Ridge	PtTe_2_	PCE	2 μm	2.7 A W^−1^ (10 mV)	0.6 MHz	Few μA	—	^54^
		PtTe_2_-Graphene	PCE	2 μm	5.5 A W^−1^ (0.5 V)	36 MHz	Few μA	—	
	Rectangular	MoTe_2_-Graphene	PVE/PGE	1.26–1.31 μm	0.2 A W^−1^ (3 V)	24 GHz	2 μA	—	^44^
	Ridge	BP-MoTe_2_	PVE	2.8–4 μm	0.85 A W^−1^ (0 V)	>10 MHz	Few μA	8.2 pW Hz^−1/2^	^245^
	Rectangular	MoTe_2_-Graphene	PCE	1.31 μm	567 mA W^−1^ (1 V)	4.81 GHz	4.16 μA	0.048 nW Hz^−1/2^	^248^
	Rectangular	MoTe_2_-Graphene	PCE	1.55 μm	20 mA W^-1^ (1 V)	3.85 GHz	4.16 μA	1.37 nW Hz^−1/2^	^248^
	Ridge	WS_2_-HfS_2_	PVE	3.8-5.5 μm	50 μA W^−1^ (3 V)	—	Few μA	1 μW Hz^−1/2^	^246^

*NEP* noise equivalent power, *SPP* surface plasmon polariton, *PhC* photonic crystal, *MRR* microring resonator, *PVE* photovoltaic effect, *PCE* photo-conductivity effect, *PGE* photo-gating effect, *IPE* internal photon emission, *PTE* photo-thermoelectric effect, *BOL* bolometric

### Silicon nitride waveguides

Silicon waveguide-integrated 2D-material infrared photodetectors have seen significant advancements, with various optimization strategies proposed to enhance their performance^249^. However, silicon’s relatively high carrier concentration as a semiconductor material leads to leakage currents, which degrade the signal-to-noise ratio, raise energy consumption, and reduce both device stability and efficiency^250^. Additionally, while silicon’s bandgap of approximately 1.1 eV allows for low transmission loss in the near-infrared range, losses increase significantly at longer or shorter wavelengths, limiting its applicability across a broader spectral range. To overcome these challenges, silicon nitride waveguides offer an effective solution owing to their large bandgap of approximately 5 eV and outstanding insulating properties^251^. In photodetectors, silicon nitride waveguides simultaneously suppress carrier injection and minimize optical transmission losses, thereby enhancing overall photodetection efficiency. Thus, silicon nitride waveguides emerge as an attractive alternative for facilitating the progress of 2D-material infrared photodetectors.

#### Graphene

**Fig. 10:**
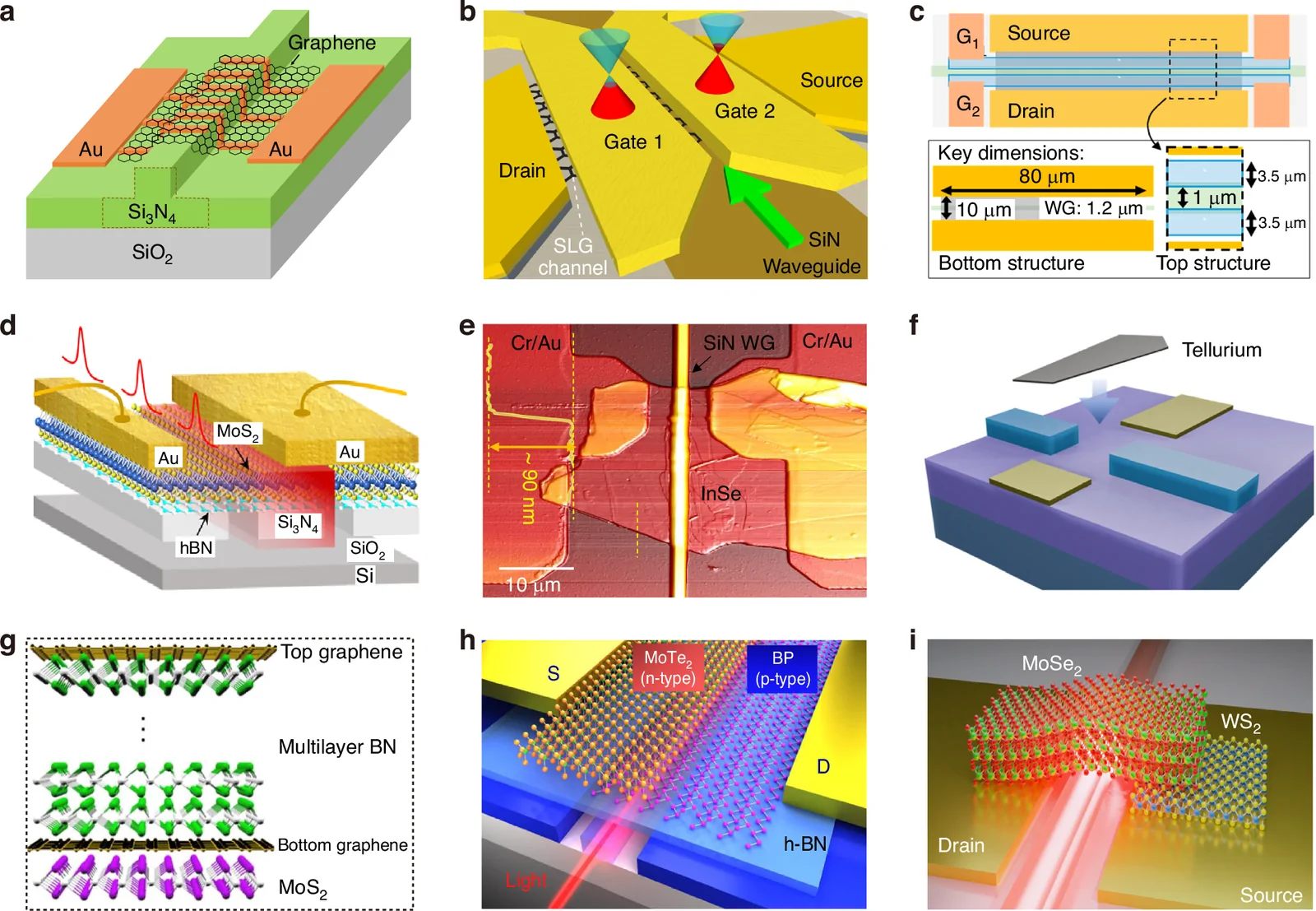
2D material infrared photodetectors based on silicon nitride waveguides. **a** Architecture of a graphene photodetector featuring interdigitated metal electrodes^254^. Reproduced from ref. ^254^ with permission of AIP Publishing. **b** Architecture of a graphene photodetector featuring a split-gate design^257^. Reproduced from ref. ^257^ with permission of Copyright © 2019 American Chemical Society. **c** Architecture of an infrared photodetector employing a graphene-polymer-graphene configuration^258^. Reproduced from ref. ^258^ under (CC BY 4.0). **d** The structure of a MoS_2_ photodetector operating via the hot-electron-assisted mechanism^260^. Reproduced from ref. ^260^ with permission of Copyright © 2022 American Chemical Society. **e** Atomic force microscope (AFM) image of an InSe photodetector integrated with the silicon nitride waveguide^262^. Reproduced from ref. ^262^ under (CC BY 4.0). **f** The structure of a Te photodetector integrated with the planar waveguide^263^. Reproduced from ref. ^263^ with permission of © The Royal Society of Chemistry 2024. **g** Heterostructure consisting of monolayer MoS_2_/monolayer graphene/multilayer h-BN/monolayer graphene^91^. Reproduced from ref. ^91^ under (CC BY 4.0). **h** Schematic layout of the BP-MoTe_2_ heterojunction photodetector coupled to a silicon nitride waveguide^51^. Reproduced from ref. ^51^ under (CC BY 4.0). **i** Schematic illustration of the MoSe_2_-WS_2_ heterostructure photodetector integrated on a silicon nitride waveguide^265^. Reproduced from ref. ^265^ under (CC BY 4.0)

In 2015, Wang et al. presented a graphene photodetector integrated with a silicon nitride waveguide^252^. The photodetector functioned via the PTE mechanism under zero bias and the BOL mechanism under nonzero bias, achieving a maximum responsivity of 126 mA W^−1^ and a dynamic response of 1 kHz within the telecommunication band. As previously discussed, optimizing the electrodes is essential for improving photodetectors’ performance. Building on this concept, in 2018, Gao et al. proposed various strategies for optimizing electrode-graphene contact to further improve the device’s photoelectric conversion efficiency and response characteristics^253–255^. First, they implemented a GND-Signal-GND geometry, in which a 70 nm-wide metal strip was placed atop the ridge waveguide as the signal electrode, with two GND electrodes positioned on either side of the ridge^253^. This configuration enabled the generation of surface plasmon polaritons by the top metal strip, enhancing the light-graphene interaction. Although silicon nitride had a lower relative permittivity than silicon, which could reduce device capacitance by roughly half compared to conventional silicon waveguide photodetectors and improve bandwidth, certain limitations remained. Specifically, the relatively high contact resistance between graphene and metal, combined with a carrier transport channel length of approximately 3 μm, resulted in a total device resistance of around 300 Ω and limited the carrier transit speed, thereby constraining the device’s bandwidth performance. Nevertheless, this photodetector still achieved a 30 GHz bandwidth at a wavelength of 1550 nm, demonstrating excellent high-speed response. To further shorten the carrier transport path, Gao et al. proposed an interdigitated metal contact design, as illustrated in Fig. 10a^254^. In this design, finger electrodes were periodically arranged, each with a width and spacing of 200 nm, effectively limiting the carrier transport length to 200 nm. This optimized structure enabled the photodetector to have a bandwidth of up to 33 GHz at 1550 nm. Furthermore, they simulated carrier transport within the channel using classical diffusion equations. The simulation results showed that when the channel length exceeded 1 μm, approximately 21.7% of the photogenerated holes extended across the entire channel. These holes not only trapped carriers along their path but also, when combined with graphene’s short carrier lifetime, further decreased the chances of carriers successfully crossing the channel to generate photocurrent. In contrast, when the channel length was scaled down to 0.2 μm, the proportion of holes spanning the entire channel dropped to just 3.7%, allowing more than 94.2% of carriers to reach the electrodes and participate in photoelectric conversion. These results demonstrate the critical importance of minimizing channel spacing for achieving high-performance graphene photodetectors. In addition, they introduced a grating-like metal contact structure design to minimize the transport channel length further, pushing the bandwidth of silicon nitride waveguide-integrated graphene photodetectors to 38 GHz^255^.

Although the aforementioned graphene photodetectors have achieved high bandwidth performance through electrode structure optimization, they still face the challenge of significant dark current under an applied bias voltage. To overcome this limitation, researchers have begun exploring graphene photodetectors integrated with silicon nitride waveguides based on the PTE mechanism, enabling zero-bias operation and thereby effectively eliminating dark current. Importantly, graphene’s exceptional thermal conductivity and tunable Seebeck coefficient make it an excellent candidate for building high-bandwidth, low-noise, and zero-bias PTE-based photodetectors^256^. In 2019, Muench et al. demonstrated a silicon nitride waveguide-integrated graphene photodetector featuring two metal split gates with a 100 nm gap, as shown in Fig. 10b^257^. The split-gate structure served a dual purpose. First, it enabled doping modulation in graphene to create an asymmetric structure, which was essential for photocurrent generation via the PTE mechanism. Second, its narrow spacing facilitated the generation of surface plasmon polaritons, enhancing the localized optical field and significantly improving graphene’s light-absorption efficiency. By tuning the gate voltages to establish an asymmetric p-n structure, the device achieved a peak responsivity of 12.2 V W^-1^ and a 3 dB bandwidth of 42 GHz. In 2020, Mišeikis et al. proposed another silicon nitride waveguide-integrated photodetector with a graphene-polymer-graphene architecture, in which the top graphene layer served as a split gate to electrostatically create a p–n junction, as presented in Fig. 10c^258^. Notably, unlike conventional dielectric materials such as aluminum oxide, which were commonly obtained by deposition processes, the polymer polyvinyl alcohol did not degrade graphene’s carrier mobility or residual carrier concentration. Instead, it could enhance the Seebeck coefficient, ultimately improving photoelectric conversion efficiency under the PTE mechanism and realizing a bandwidth of 67 GHz.

#### TMDs

In recent years, several other 2D materials originally integrated into silicon waveguide photodetectors have been gradually applied to silicon nitride waveguide platforms. These integrations retain the outstanding optoelectronic characteristics of 2D materials while leveraging the benefits of silicon nitride waveguides, like low optical loss and broad spectral response, thereby delivering promising infrared photodetection performance. Recently, Wang et al. implemented a high-efficiency MoTe_2_ photodetector integrated with a silicon nitride waveguide, reaching a large responsivity of 41.9 A W^−1^, a detectivity of 1.62 × 10^11^ Jones, and an EQE as high as 3360% at a wavelength of 1550 nm under a bias of 50 mV^259^. This impressive performance was mainly due to two key factors. First, the silicon nitride waveguide structure significantly extended the optical propagation length within MoTe_2_, enhancing light absorption. Second, the creation of an Ohmic contact between metal electrodes and MoTe_2_ thin film effectively lowered the contact barrier, allowing for more optimized collection of photogenerated carriers and resulting in a stronger photocurrent. Nevertheless, due to the bandgap of some TMDs exceeding 1.0 eV, they are unable to absorb light in the communication band directly. To address this limitation, Li and Zhang et al. developed a hot electron-assisted optical detection scheme in 2022^89,260^. Thin-film MoS_2_ served as a promising hot-electron acceptor because of its multiple trap interfaces that facilitated internal optical amplification. Based on this mechanism, Li et al. developed a device through integrating a few-layer MoS_2_ with a silicon nitride waveguide, where one metal electrode was vertically positioned above the waveguide while the other was laterally placed away from the waveguide^260^, as presented in Fig. 10d. The top metal electrode interacted with the evanescent field of the telecom band waveguide mode, generating hot electrons through surface plasmon excitation. When the energy of hot electrons emitted from the metal electrode exceeded the Schottky barrier created at the metal-MoS_2_ interface, MoS_2_ could capture these hot electrons through an internal photoelectric process, converting them into photocurrent. This photodetector demonstrated a responsivity of 15.7 mA W^−1^ at 1550 nm wavelength. To boost the responsivity of MoS_2_-based photodetectors, Zhang et al. proposed a MoS_2_ hot electron photodetector built on a silicon nitride microring resonator^89^. The evanescent field in the silicon nitride microring resonators was coupled to the Au-MoS_2_ Schottky junction region, effectively enhancing the injection efficiency of hot electrons. This optimized design achieved a significantly improved responsivity of 154.6 mA W^−1^ at 1516 nm wavelength, with rapid optical response characteristics demonstrated by rise and fall times of 9.1 and 11.3 μs, respectively.

#### Other emerging 2D materials

Recently, layered InSe has garnered growing interest owing to its remarkable characteristics, like thickness-dependent tunable bandgap, anisotropic optical response, and large carrier mobility of approximately 10^4 ^cm^2 ^V^−1^ s^−1^, demonstrating its applicability to high-performance infrared photodetectors^261^. For instance, Tamalampudi et al. integrated a 90 nm-thick multilayer InSe flake onto the surface of a silicon nitride waveguide, as displayed in Fig. 10e^262^. Leveraging the high refractive index contrast (~0.4) between InSe and the silicon nitride waveguide, they enhanced the local electric field and increased both the mode penetration depth and optical power density within the InSe layer, thereby significantly improving its absorption coefficient to 0.11 dB μm^−1^. Ultimately, the device achieved a low dark current density and a responsivity of 0.38 A W^−1^ at a wavelength of 976 nm. Moreover, Te has been effectively incorporated into silicon nitride platforms, making it possible to create high-efficiency photodetectors. In 2024, Xiao et al. introduced an innovative structure for the integration of Te with silicon nitride waveguides^263^, as shown in Fig. 10f. The researchers deposited titanium dioxide onto a silicon nitride substrate to fabricate a single transverse magnetic mode waveguide. They designed a narrow 15 μm gap between the input and output waveguides and positioned a thin film of Te within this gap. This design provided two key advantages. First, it prevented cracks caused by non-planar deformation when depositing 2D materials on the waveguide surface, preserving material quality. Second, it enabled strong optical-field coupling and enhanced Te absorption efficiency, achieving high responsivity with an impressively short device length of just 15 μm. Consequently, the device delivered a responsivity of 0.5 A W^−1^ and a bandwidth of 12 GHz at the wavelength of 1570 nm.

#### 2D Material heterostructures

While single-type 2D materials have shown promising detection capabilities on silicon nitride waveguides, the integration of heterojunctions using multiple 2D materials offers potential pathways for further enhancing photodetector performance^264^. In 2019, Gao et al. presented the first realization of a heterostructured tunnel photodiode integrated on a silicon nitride waveguide^91^. The heterostructure, composed of a monolayer MoS_2_, a monolayer graphene, a multilayer boron nitride, and another monolayer graphene, was sequentially stacked on the waveguide from bottom to top, as illustrated in Fig. 10g. In this structure, MoS_2_ passivated the interfacial states between graphene and silicon nitride and introduced n-doping to the bottom graphene layer, while the top graphene layer was p-doped through contact with air and metal. A multilayer boron nitride layer served as a tunnel barrier between the two graphene layers, forming a vertically oriented p-i-n tunnel photodiode on the waveguide. The tunneling barrier effectively suppressed dark current to the nA level, thereby reducing noise, while the high carrier mobility of the top and bottom graphene layers significantly enhanced the device’s response speed. In addition, unlike conventional heterostructures where light absorption was mainly confined to the narrow junction region, this device placed the entire heterostructure above the waveguide, effectively expanding the light-material interaction area and improving absorption efficiency. As a result, it presented a responsivity of 0.24 A W^−1^ at 1550 nm, a bandwidth of 28 GHz, and an on/off current ratio greater than 10^4^, demonstrating outstanding overall performance. In 2022, Tian et al. demonstrated a heterojunction photodetector consisting of naturally p-type BP and n-type MoTe_2_, requiring no additional doping, as shown in Fig. 10h^51^. The photodetector exploited a built-in electric field directed from MoTe_2_ to BP, which effectively suppressed the dark current and enabled self-powered photodetection. Furthermore, the heterojunction structure allowed for electrical tuning via a highly doped silicon substrate serving as a back gate. As the gate voltage increased from −60 to 60 V, the responsivity of the device continuously improved from 279 to 709 mA W^−1^, and the dark current was gradually reduced from 54 to 8.5 nA, which was approximately two orders of magnitude lower than that of previously mentioned waveguide-integrated BP photodetectors. In 2023, Gherabli et al. reported a MoSe_2_/WS_2_ van der Waals type-II junction photodiode integrated on a silicon nitride waveguide for near-infrared detection at 780 nm^265^, as illustrated in Fig. 10i. In this device, an n-type monolayer WS_2_ is inserted between a p-type multilayer MoSe_2_ and one metal contact, which provides an efficient transport path for electrons while introducing a sizeable barrier for hole injection, thereby strongly suppressing the dark current. The photodiode achieves a dark current as low as about 50 pA, whereas a MoSe_2_-only reference device exhibits a dark current of around 0.5 nA. Under reverse bias, the detector delivers a responsivity of approximately 1 A W^−1^ and a 3 dB bandwidth of about 20 MHz, with a noise level corresponding to an NEP on the order of 10^−12 ^W Hz^−1/2^. Benefiting from the low dark current and on-chip integration, the authors further cascaded the detector with a silicon nitride microring resonator on the same waveguide and used the photocurrent readout to characterize the ring response, demonstrating the suitability of such heterojunction detectors for integrated sensing and filtering functions. For comparison, Table 4 outlines the structures and key performance metrics of the silicon nitride waveguide-integrated 2D-material infrared photodetectors discussed in this section.

**Table 4:** Structures and key properties of silicon nitride waveguide-integrated 2D-material infrared photodetectors

Waveguide		2D material	Mechanism	Performance					Ref.
Material	Structure			Wavelength	Responsivity	Bandwidth	Dark current	NEP or D*	
Silicon nitride	Ridge	Graphene	BOL	1.55 μm	126 mA W^−1^ (8 V)	1 kHz	Few μA	—	^252^
	Hybrid SPP	Graphene	PVE	1.55 μm	15 mA W^−1^ (0 V)	30 GHz	—	—	^253^
	Ridge	Graphene	PCE	1.55 μm	2.36 A W^−1^ (1 V)	33 GHz	Few μA	3.33 pW Hz^−1/2^	^254^
	Ridge	Graphene	PCE	1.55 μm	13 mA W^−1^ (0.1 V)	38 GHz	<100 μA	—	^255^
	SPP slot	Graphene	PTE	1.51−1.58 μm	12.2 V W^−1^	42 GHz	None	600 pW Hz^−1/2^	^257^
	Rectangular	Graphene	PTE	1.55 μm	6 V W^−1^	>67 GHz	None	—	^258^
	Rectangular	MoTe_2_	PCE	1.55 μm	41.9 A W^−1^ (50 mV)	—	169.7 nA	16.2 × 10^10^ Jones	^259^
			PCE	0.8−1 μm	15.4 A W^−1^ (50 mV)	—	9.7 nA	59.6 × 10^9^ Jones	
	Ridge	MoS_2_	IPE	1.55 μm	15.7 mA W^−1^ (0.3 V)	1.37 GHz	Few μA	—	^260^
	MRR	MoS_2_	IPE	1.5−1.63 μm	154.6 mA W^−1^ (1 V)	—	Few μA	—	^89^
	Rectangular	InSe	PCE	0.976 μm	0.38 A W^−1^ (5 V)	85 MHz	Few μA	4.7 nW Hz^−1/2^	^262^
	Slab	Te	PCE	1.57 μm	0.5 A W^−1^ (1 V)	12 GHz	44.1 μA	—	^263^
	Ridge	MoS_2_-Graphene	DT/F-N tunneling	1.55 μm	0.24 A W^−1^ (10 V)	28 GHz	Few nA	—	^91^
	Rectangular	MoTe_2_-BP	PVE/PCE	1.5−1.63 μm	709 mA W^−1^ (1 V)	~1 GHz	<7 nA	—	^51^
	Rectangular	MoS_2_-BP	PVE	1.5274 μm	11.26 mA W^−1^ (0 V)	—	0.19 nA	—	^264^
	Rectangular	MoSe_2_-WS_2_	PGE	0.78 μm	1 A W^−1^ (2 V)	20 MHz	50 pA	1 pW Hz^−1/2^	^265^

*NEP* noise equivalent power, *SPP* surface plasmon polariton, *MRR* microring resonator, *PVE* photovoltaic effect, *PCE* photo-conductivity effect, *IPE* internal photon emission, *PTE* photo-thermoelectric effect, *BOL* bolometric, *DT* direct tunneling, *F-N tunneling* Fowler-Nordheim tunneling

### TFLN waveguides

As an emerging platform for PICs, lithium niobate has attracted significant attention for its excellent electro-optic modulation capabilities, large spectral transparency window, low optical loss, and strong nonlinear optical properties^266^. These characteristics make it ideal for applications in high-speed modulation, frequency conversion, and quantum information processing. However, traditional bulk lithium niobate platforms typically rely on waveguide fabrication techniques such as titanium diffusion or proton exchange, which result in weak mode confinement and large device footprints, thereby limiting their scalability and integration density in advanced photonic chips^267^. To address these issues, researchers have developed TFLN platforms by transferring ultrathin lithium niobate films onto insulating substrates, enabling the formation of waveguides with high refractive index contrast and strong mode confinement^268^. Owing to its exceptional linear electro-optic coefficient and broad optical transparency window, TFLN has become the platform of choice for high-speed electro-optic modulation. However, its intrinsic transparency from the visible to the near-infrared suppresses light absorption, making conventional photodetection schemes infeasible on TFLN. To overcome this, researchers have explored various photoconversion approaches, such as depositing amorphous silicon on TFLN for visible-light detection^269^ or integrating InP to capture communication-band photons^270^. These methods introduce high-loss interfaces and lattice-mismatch challenges, which limit process compatibility, response speed, and scalability. By contrast, heterointegration of 2D materials, with their dangling-bond-free surfaces and superior optoelectronic properties, onto TFLN waveguides has emerged as a highly promising route for on-chip photodetection. Leveraging the excellent optical properties and design flexibility of the TFLN platform, 2D materials have demonstrated great potential in achieving strong light-matter coupling, high bandwidth, and high responsivity^271^. This integration offers new strategies and technological pathways for developing next-generation, high-performance, on-chip 2D material infrared photodetectors.

**Fig. 11:**
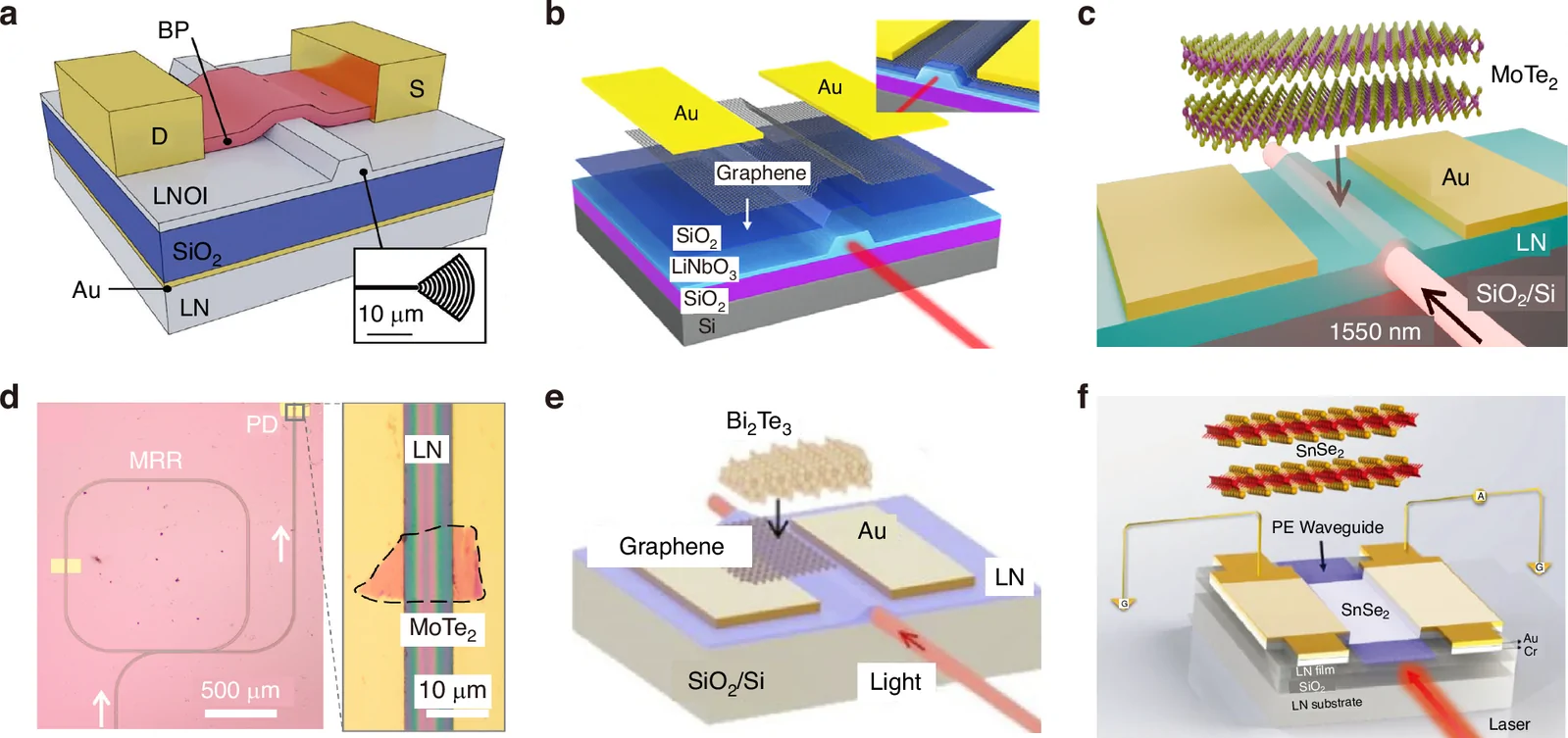
2D material infrared photodetectors based on TFLN waveguides. **a** Schematic layout of a BP photodetector implemented on a TFLN ridge waveguide platform^52^. Reproduced from ref. ^52^ under (CC BY 4.0). **b** Schematic of a graphene-based photodetector coupled to a TFLN ridge waveguide^53^. Reproduced from ref. ^53^ under (CC BY 4.0). **c** Schematic layout of a MoTe_2_-based photodetector implemented on a TFLN ridge waveguide^273^. Reproduced from ref. ^273^ under (CC BY 4.0). **d** Microscopic image of a MoTe_2_ photodetector interconnected with the microring resonator^273^. Reproduced from ref. ^273^ under (CC BY 4.0). **e** Schematic layout of a Bi_2_Te_3_-graphene heterojunction photodetector implemented on a TFLN ridge waveguide^275^. Reproduced from ref. ^275^ with permission of Optica Publishing Group. **f** Schematic layout of a SnSe_2_-based photodetector coupled to a proton-exchanged TFLN waveguide^100^. Reproduced from ref. ^100^ with permission of John Wiley and Sons

In 2022, Wang et al. integrated a TFLN waveguide with a 15 nm-thick, 10 μm-long BP flake to realize photodetection in the communication band (1510–1640 nm), as displayed in Fig. 11a^52^. Apart from investigating the influence of BP thickness and length on light absorption, the researchers also explored BP’s in-plane anisotropy, particularly the angle between the armchair crystal orientation and the direction of waveguide mode propagation. Their findings demonstrated that aligning the armchair direction with the waveguide mode propagation could significantly improve the light absorption of BP, which resulted in a responsivity of 148 mA W^−1^ and a lower frequency bandwidth of 3.5 MHz at 1550 nm under a bias voltage of 0.3 V. Subsequently, Xue et al. implemented a TFLN waveguide-integrated BP photodetector with improved light absorption by modulating the BP thickness to 40 nm and extending the interaction length with the waveguide to 40 μm^272^. This optimization resulted in a significantly enhanced responsivity of 2.64 A W^−1^ and a 3 dB bandwidth of 1.97 GHz, further confirming the effectiveness of structural parameter tuning in improving device performance. In 2023, Zhu et al. successfully fabricated TFLN waveguide-based infrared photodetectors using both graphene and Te as active materials, broadening the range of materials available for the TFLN photodetection platform, with the graphene photodetector structure presented in Fig. 11b^53^. Thanks to the outstanding carrier mobility of graphene, the device achieved a responsivity of 17.27 mA W^−1^ and a bandwidth of 40 GHz at 1550 nm, while the built-in electric field efficiently suppressed the dark current to 5 nA. In contrast, the Te-based photodetector leveraged its strong optical absorption to achieve a significantly higher responsivity of 7 A W^−1^ and a bandwidth exceeding 2 GHz. More recently, Dong et al. presented a MoTe_2_ photodetector integrated with a TFLN waveguide, where the waveguide was made using femtosecond laser photolithography-assisted chemical-mechanical etching, as shown in Fig. 11c^273^. They systematically examined the impact of MoTe_2_ thickness on both responsivity and dark noise and found that light absorption saturated at a thickness of 20 nm, presenting a peak responsivity of 32.6 mA W^−1^. Besides, the increase in thickness led to a gradual decrease in the Schottky barrier height and a noticeable rise in dark current, indicating the need to balance responsivity and dark current in practical applications based on specific performance requirements. Additionally, they integrated a microring resonator with the MoTe_2_ photodetector on the TFLN platform (Fig. 11d), further demonstrating the compatibility of the photodetector with a variety of photonic components.

Recently, heterostructures formed from 2D materials have been investigated on TFLN platforms for photodetection, leveraging their synergistic optoelectronic advantages and revealing a new direction for development. For example, in 2024, Xia et al. fabricated a van der Waals heterostructure photodetector on the TFLN platform by combining p-type BP and n-type MoS_2_ layers^274^. The photodetector showed a dark current of 0.21 nA and a switching ratio of over 200 at zero bias. Under a −1 V bias, it demonstrated a responsivity of 1.46 A W^−1^ with a dark current of 2.33 μA. Compared to previously reported BP-MoS_2_ photodetectors integrated on silicon nitride waveguides, this device exhibits superior responsivity but slightly lower frequency response, making it more suitable for applications where high-speed operation is not critical, like optical sensing. Furthermore, Yuan et al. developed infrared photodetectors using heterostructures composed of graphene and the topological insulator Bi_2_Te_3_, seamlessly integrated with the TFLN waveguide (Fig. 11e)^275^. The topological insulator Bi_2_Te_3_, characterized by its unique Dirac surface states, warping effects, narrow bandgap, and high carrier mobility, formed a heterojunction with graphene to enhance light absorption and suppress dark current, enabling highly efficient photodetection. The photodetector demonstrated a responsivity of 2.54 mA W^−1^ at a wavelength of 1550 nm and a bandwidth of 4.7 GHz under zero bias voltage, representing a 700% improvement in responsivity compared to the pure graphene-based photodetector.

In addition to ridge TFLN waveguides with slanted sidewalls, proton-exchanged TFLN waveguides offer an alternative platform for 2D material photodetection. Their relatively flat surface profile effectively minimizes stress and deformation in the 2D material, although they impose comparatively weaker confinement on the optical mode^276^. The fabrication process of proton-exchanged TFLN waveguides can be described as follows: at high temperatures, hydrogen ions from a proton source exchange with lithium ions in lithium niobate, thereby altering the refractive index of the material and forming a waveguide, which is similar to the fabrication of bulk lithium niobate^100^. In 2023, Hu et al. employed the proton-exchanged method to fabricate a TFLN planar waveguide and integrated it with BP to construct high-performance infrared photodetectors^277^. Benefiting from the planar waveguide structure, which could reduce stress and defects in the 2D material, the photodetector exhibited a high responsivity of 147 A W^−1^ and a 3 dB bandwidth of 0.86 GHz at the wavelength of 1550 nm. In 2024, Chen et al. successfully integrated SnSe_2_ onto proton-exchanged TFLN waveguides, developing a high-sensitivity bidirectional optical response waveguide photodetector (Fig. 11f)^100^. As an emerging TMD, SnSe_2_ has a wide bandgap of 1.07 eV and displays efficient optical absorption, particularly for wavelengths below 1159 nm. Consequently, the device displayed distinct response mechanisms depending on the incident wavelength: at 850 nm, the photodetector operated primarily through the PCE mechanism, producing a positive photoresponse, whereas under 1550 and 1310 nm illumination, the BOL effect dominated, resulting in a negative photoresponse. The photodetector realized impressive responsivities of 761.78 and 3.13 A W^−^^1^ at 850 and 1550 nm, respectively. Lately, Yang et al. presented a MoTe_2_ photodetector based on a proton-exchanged TFLN waveguide, featuring low dark current, high responsivity, and self-powered operation enabled by a built-in electric field arising from a slight asymmetry in the contacts between MoTe_2_ and two metal electrodes^101^. At zero bias and a wavelength of 1310 nm, the photodetector achieved a dark current of approximately 20 pA and an on/off ratio exceeding 10^5^. Under a bias voltage of 1 V, it exhibited a dark current of 1.13 nA and a responsivity of 309 mA W^−1^. Although proton-exchanged waveguides provide a smooth and continuous transfer interface for 2D materials, which helps preserve their structural quality to some extent, the relatively low refractive index contrast results in weak optical confinement, limiting the strength of light-material interaction. In addition, this waveguide structure faces compatibility challenges when integrated with other advanced lithium niobate photonic devices, which hinders its potential for use in high-density integrated platforms^278^.

### Other waveguides

#### Chalcogenide glass waveguides

**Fig. 12:**
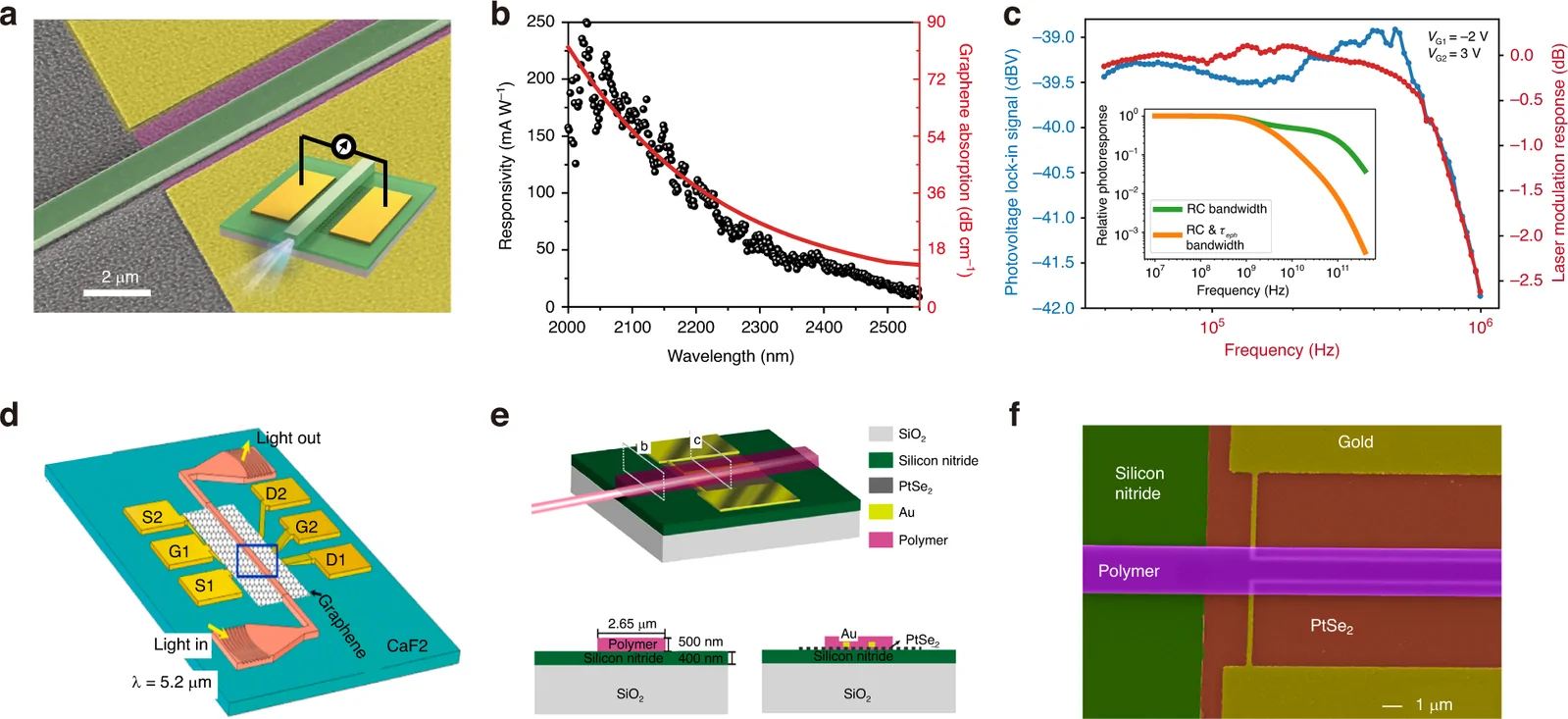
2D material infrared photodetectors based on chalcogenide glass and polymer waveguides. **a** Schematic layout of a graphene-based photodetector implemented on a chalcogenide glass waveguide^41^. Reproduced from ref. ^41^ with permission of Springer Nature. **b** Responsivity profile of the graphene photodetector integrated with the chalcogenide glass waveguide^41^. Reproduced from ref. ^41^ with permission of Springer Nature. **c** Measured bandwidth of a graphene photodetector supported by a CaF_2_ substrate^50^. Reproduced from ref. ^50^ under (CC BY 4.0). **d** Schematic layout of a graphene photodetector coupled to a dual-core chalcogenide glass waveguide^102^. Reproduced from ref. ^102^ with permission of © 2024 Elsevier Ltd. **e** Schematic layout of a PtSe_2_-based photodetector implemented on a polymer waveguide platform^46^. Reproduced from ref. ^46^ with permission of Copyright © 2020 American Chemical Society. **f** Scanning electron micrograph (false color) of the PtSe_2_ photodetector implemented on a polymer waveguide^46^. Reproduced from ref. ^46^ with permission of Copyright © 2020 American Chemical Society

To address the potential issue of fracture that may occur when transferring 2D materials onto waveguides, directly growing an optical-thickness dielectric film on the 2D material is an ideal solution. Chalcogenide glass is an emerging waveguide platform that can be deposited onto 2D materials at high rates through single-source thermal evaporation under near-room temperature conditions without compromising the structure or optoelectronic properties of the 2D material^279^. In 2018, Lin et al. showed an infrared photodetector with the chalcogenide glass waveguide structure directly deposited on graphene^41^, as shown in Fig. 12a. Benefiting from the excellent infrared transparency of chalcogenide glass and the broadband absorption enabled by graphene’s zero bandgap, the photodetector displayed a wide spectral response spanning the 2.0–2.55 μm range, with a peak responsivity of 250 mA W^−1^ at a wavelength of 2.03 μm (Fig. 12b). By fitting the experimental data with three potential photocurrent generation mechanisms, the analysis identified the PVE as the dominant operating mechanism. Notably, this work demonstrated a waveguide-integrated graphene photodetector on a flexible polymer substrate for the first time, achieving mechanical flexibility in integrated photonic systems. However, owing to the high mid-infrared absorption of SiO_2_ substrates, the operational wavelength of chalcogenide glass waveguide-integrated graphene photodetectors fabricated on SiO_2_ was limited to below 3.8 μm. To overcome this limitation, Goldstein et al. incorporated a CaF_2_ substrate with extended infrared transparency of up to 8 μm into the chalcogenide glass waveguide-integrated graphene photodetector, and employed a gate-tunable PTE design, thereby enhancing both the operational wavelength range and the zero-bias sensitivity of the device^50^. The resulting device not only extended the response wavelength to 5.2 μm but also exhibited an anticipated bandwidth exceeding 1 GHz (Fig. 12c), offering new opportunities for developing compact, on-chip mid-infrared spectroscopic sensing systems. Recently, Hashemnezhad et al. further optimized this structure by proposing an innovatively dual-core chalcogenide glass waveguide, as presented in Fig. 12d^102^. This enhancement doubled the interaction distance between graphene and light while also achieving a twofold increase in responsivity compared to the work of Goldstein et al., reaching a maximum of 6.3 V W^-1^. In addition to graphene, Deckoff-Jones et al. successfully achieved a BP photodetector employing a chalcogenide glass waveguide, which demonstrated a responsivity of 40 mA W^−1^ at the wavelength of 2185 nm^280^. This achievement robustly validates the technical feasibility of integrating chalcogenide glass with various 2D materials.

#### Polymer waveguides

Similar to chalcogenide glass waveguides, polymer waveguides offer high platform compatibility by allowing the flexible selection of substrate materials. Unlike conventional waveguides that rely on total internal reflection, polymer waveguides guide light based on the concept of bound states in the continuum (BIC) by utilizing their intrinsically low refractive index on top of a high-index substrate^281^. This mechanism enables a fabrication process in which 2D materials are first transferred onto a flat substrate surface, followed by the deposition of the polymer waveguide structure. This approach minimizes the risk of material rupture caused by surface irregularities or stress concentration and also simplifies the overall device fabrication. As a result, it offers a potential strategy for developing high-efficiency, flexible, and highly compatible optoelectronic devices. In 2020, Wang et al. showed a PtSe_2_ photodetector built on a polymer waveguide, as demonstrated in Fig. 12e^46^. In this design, the planar silicon nitride structure offered a complete platform for the integration of 2D materials, while light was guided through the low-refractive-index polymer waveguide based on the concept of BIC. The two metal strips could not only enhance optical field localization to strengthen light–material interaction but also provide an electric field to efficiently separate and collect photogenerated electron-hole pairs (Fig. 12f). This approach preserved the quality of the 2D materials while simultaneously improving the overlap between the waveguide’s evanescent field and the 2D material layer. Consequently, the photodetector demonstrated a responsivity of 12 mA W^−1^ and a bandwidth of 35 GHz at the wavelength of 1550 nm under 8 V bias voltage. In 2022, Chen et al. proposed a self-powered photodetector built on defect-engineered WSe_2_-graphene heterojunctions integrated with a polymer waveguide^106^. In this structure, ion beam irradiation of WSe_2_ created sulfur vacancies, which formed a Schottky junction with the unirradiated region, enabling self-powered operation. This photodetector exhibited a broadband optical response from 450 to 1550 nm and an on/off ratio of 10^4^ at zero bias. However, polymer waveguides suffer from relatively high optical losses and poor thermal stability, thus requiring further optimization for high-performance photonic integration applications. For comparison purposes, Table 5 summarizes the structures as well as key performance metrics of 2D-material infrared photodetectors integrated with TFLN waveguides, chalcogenide glass waveguides, and polymer waveguides.

**Table 5:** Structures and key properties of TFLN, chalcogenide glass, and polymer waveguide-integrated 2D-material infrared photodetectors

Waveguide		2D material	Mechanism	Performance					Ref.
Material	Structure			Wavelength	Responsivity	Bandwidth	Dark current	NEP or D*	
TFLN	Ridge	BP	PCE	1.51–1.64 μm	148 mA W^−1^ (0.3 V)	>3.5 MHz	Few μA	—	^52^
	Ridge	BP	PCE	1.52–1.58 μm	2.64 A W^−1^ (2 V)	1.97 GHz	Few mA	1.49 × 10^8^ Jones	^272^
	Ridge	Graphene	PVE	1.5–1.63 μm	49 mA W^−1^ (0.3 V)	~40 GHz	5 nA	3.37 × 10^11^ Jones	^53^
		Te	PCE	1.5–1.63 μm	7 A W^−1^ (0.5 V)	2 GHz	<300 μA	—	
	Ridge	MoTe_2_	PVE	1.55 μm	1 mA W^−1^ (20 V)	—	1.6 nA	—	^273^
	Ridge	BP-MoS_2_	PVE	1.48–1.58 μm	1.46 A W^−1^ (1 V)	—	0.21 nA	—	^274^
	Ridge	Bi_2_Te_3_-Graphene	PVE	1.53–1.6 μm	2.54 mA W^−1^ (0 V)	4.7 GHz	Few μA	—	^275^
	Ridge	Bi_2_Te_3_	PTE	1.55 μm	60 mA W^−1^	—	Few μA	—	^271^
	Rectangular	BP	PCE	0.4–1.55 μm	147 A W^−1^ (0.5 V)	0.86 GHz	73 μA	3.04 × 10^13^ Jones	^277^
	Rectangular	SnSe_2_	PGE	0.85 μm	761.78 A W^−1^ (1 V)	—	Few μA	1.03 × 10^12^ Jones	^100^
			BOL	1.55 μm	3.13 A W^−1^ (1 V)	—		7.06 × 10^9^ Jones	
	Rectangular	MoTe_2_	PVE	1.31 μm	309 mA W^−1^ (1 V)	37 kHz	1.13 nA	3.88 pW Hz^−1/2^	^101^
				1.55 μm	13.8 mA W^−1^ (1 V)	8 kHz		0.217 nW Hz^−1/2^	
	Rectangular	MoTe_2_	PVE	1.31 μm	70 mA W^−1^ (0 V)	2.2 kHz	25 pA	7 × 10^8^ Jones	^268^
	Rectangular	MoS_2_	IPE	1.31 μm	68.7 mA W^−1^ (1 V)	—	2 pA	—	^276^
Chalcogenide glass	Rectangular	Graphene	PVE	2–2.55 μm	250 mA W^−1^ (1.5 V)	—	—	—	^41^
	Rectangular	Graphene	PTE	5.2 μm	1.5 V W^−1^	>1 GHz	—	1.1 nW Hz^−1/2^	^50^
	Rectangular	Graphene	PTE	5.2 μm	6.3 V W^−1^	1.54 GHz	None	0.34 nW Hz^-1/2^	^102^
	Rectangular	BP	PGE	2.185 μm	40 mA W^−1^ (0.2 V)	—	Few μA	23 pW Hz^−1/2^	^280^
Polymer	Rectangular	PtSe_2_	PCE	1.55 μm	12 mA W^-1^ (8 V)	35 GHz	317 nA	—	^46^
	Rectangular	WSe_2_-Graphene	PVE	0.4–1.55 μm	1.8 mA W^−1^ (0 V)	100 kHz	Few nA	—	^106^

*NEP* noise equivalent power, *PVE* photovoltaic effect, *PCE* photo-conductivity effect, *PGE* photo-gating effect, *PTE* photo-thermoelectric effect, *BOL* bolometric, *IPE* internal photon emission

## Performance comparison with commercial infrared photodetectors

With the continued development of waveguide-integrated 2D-material infrared photodetectors, these devices have exhibited distinct advantages concerning compactness and integration compatibility. To assess their practical application potential, it is essential to systematically compare their key performance metrics with those of mainstream commercial photodetectors, such as Ge, InGaAs, and Si. Such a comparison helps to clarify the advantages and limitations of 2D material-based devices, providing valuable guidance for device selection and optimization in future high-speed optical communication and integrated photonic chip applications. In this context, commercial photodetectors refer to off-the-shelf discrete devices available on the market rather than research-level on-chip waveguide-integrated detectors reported in the literature. The corresponding performance data are mainly taken from product datasheets provided on the official website of Thorlabs.

**Fig. 13:**
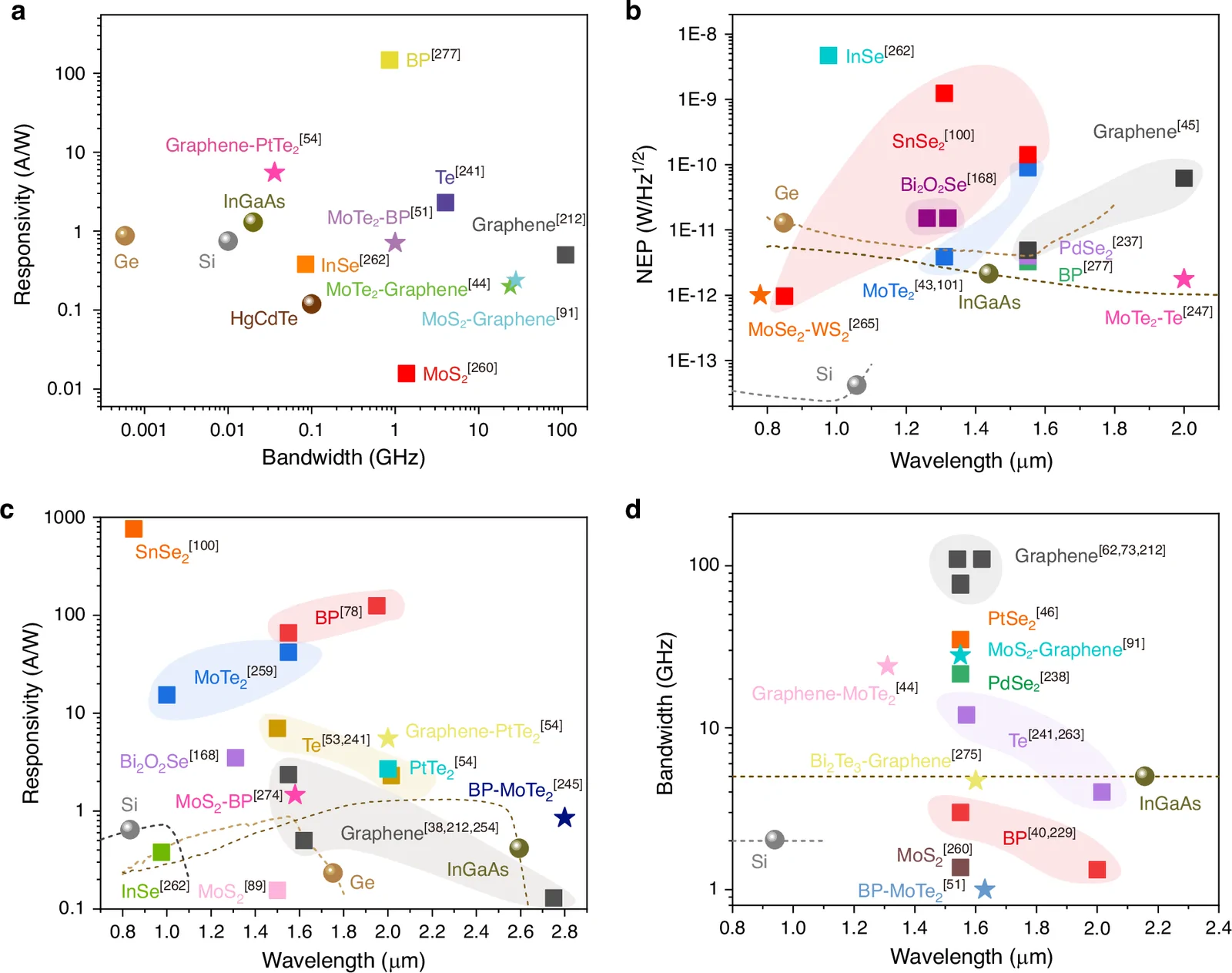
Performance comparison between waveguide-integrated 2D-material infrared photodetectors and commercial infrared photodetectors. **a** Comparison of responsivity and bandwidth. **b** Comparison of operating wavelength and NEP. **c** Comparison of responsivity and wavelength. **d** Comparison of bandwidth and wavelength. The commercial photodetector data shown in the figure were taken from the Thorlabs website, corresponding to the models PDAVJ8, PDA30B2, DET100A2, DET05D2, DET08CFC, and DET025AFC

Figure 13a presents a comparison between several high-performance waveguide-integrated 2D-material photodetectors discussed in this review and mainstream commercial photodetectors in terms of two key metrics, including responsivity and bandwidth. From the perspective of individual performance indicators, some 2D material-based photodetectors already demonstrate highly competitive characteristics. For instance, graphene-based photodetectors have achieved bandwidths up to 110 GHz, while BP-based photodetectors exhibit responsivities exceeding 100 A W^−1^. In addition, commercial photodetectors tend to show a clear trade-off between responsivity and bandwidth. For example, Ge-based detectors typically offer higher responsivity but lower bandwidth, whereas HgCdTe detectors achieve high bandwidth at the expense of lower responsivity. Notably, certain 2D materials and their heterostructures exhibit a broader distribution across both performance dimensions, with some devices even achieving a rare combination of high responsivity and high bandwidth, such as BP, PdSe_2_, MoTe_2_-Graphene, and PtTe_2_-Graphene.

Figure 13b further compares waveguide-integrated 2D-material infrared photodetectors and commercial photodetectors with respect to NEP and operating wavelength. Overall, commercial photodetectors exhibit lower NEP in the infrared region, particularly silicon-based photodetectors, which remain dominant in the market due to mature fabrication processes and excellent signal-to-noise performance. In comparison, some 2D material photodetectors still face challenges in noise control. For instance, graphene, with its zero bandgap, tends to generate higher dark currents, resulting in a relatively high NEP. However, some 2D material photodetectors have achieved NEP values in the telecommunication band (1.3–1.6 μm) that are comparable to or even lower than those of commercial photodetectors, such as devices based on BP and MoTe_2_-Te.

To provide a more intuitive comparison of photodetector performance across different wavelengths, particularly highlighting the importance of individual metrics in specific application scenarios (for example, gas sensors that require high responsivity but relatively low bandwidth), we plot the responsivity and bandwidth of commercial photodetectors and waveguide-integrated 2D-material infrared photodetectors at various wavelengths, as shown in Fig. 13c and Fig. 13d. This indicates that waveguide-integrated photodetectors exhibit outstanding advantages in single performance metrics. For instance, the responsivity of the waveguide-integrated SnSe_2_ photodetector is several hundred times higher than that of silicon-based photodetectors at the same wavelength, while the graphene-based photodetector demonstrates a bandwidth nearly one hundred times greater than that of commercial counterparts at specific wavelengths. These findings suggest that the superior performance of waveguide-integrated 2D-material photodetectors in targeted metrics offers considerable potential for applications with specific requirements in responsivity or bandwidth.

As summarized in the preceding performance comparison, certain waveguide-integrated 2D-material photodetectors have already achieved key figures of merit that are comparable to those of commercial devices. Beyond these performance metrics, they also exhibit distinctive advantages in several representative application scenarios, particularly in view of their compactness and integration compatibility. In high-speed optical communication systems, for example, conventional commercial photodetectors are typically deployed at the end of the link as discrete receiver modules, which usually require additional fiber-to-chip coupling interfaces and complex packaging. By contrast, waveguide-integrated 2D-material photodetectors can be directly embedded into PICs, enabling tight on-chip integration with waveguides, modulators, and other functional components, and thus better aligning with the requirements of chip-scale communication architectures. In recent years, these devices have moved beyond quasi-static or small-signal characterization and have been incorporated into high-speed data-transmission experiments. System-level demonstrations have been carried out under various modulation formats, including NRZ OOK and PAM4, where clear eye diagrams and acceptable bit-error-rate performance have been achieved at symbol rates from several tens up to 100 GBaud^48^. These results provide compelling evidence for the practical feasibility of waveguide-integrated 2D-material photodetectors in high-speed communication links.

On the sensing side, waveguide-integrated 2D-material photodetectors also offer advantages that are distinct from those of conventional commercial detectors. In addition to their compact footprint, the waveguide platform allows the optical mode to be engineered in a way that selectively enhances light-matter interactions, thereby improving the overall sensing sensitivity at the system level. For instance, recent studies have employed on-chip slot waveguides to strengthen the overlap between the guided field and gas molecules, enabling highly sensitive detection of gases at room temperature^209^. Micro-ring resonators represent another mature on-chip sensing structure, where sensing information is typically obtained by monitoring shifts in resonance wavelength or changes in spectral lineshape, often relying on wavelength sweeps and external spectral readout instruments^282^. If 2D-material photodetectors are further integrated with such resonant structures, variations in the resonance condition could, in principle, be directly converted into electrical readout at a fixed operating wavelength, offering a new route toward more compact and simplified on-chip sensing architectures. However, it should be emphasized that despite these promising prospects, waveguide-integrated 2D-material photodetectors still face significant challenges in terms of device stability, process uniformity, and long-term reliability. These commercialization-related issues will be discussed in more detail in the next section.

## Conclusions, challenges, and perspectives

Our review comprehensively surveys the latest advancements in waveguide-integrated 2D-material infrared photodetectors. Driven by the growing demand for on-chip, high-performance, and multifunctional optoelectronic systems, such integrated photodetectors have attracted growing research interest attributable to their unique advantages in miniaturization, high detection sensitivity, and integration compatibility. We have systematically reviewed key developments in this field, including waveguide materials and configurations, types of 2D materials, working mechanisms, device architectures, and performance metrics. Investigations have revealed that the integration of 2D materials with waveguide structures can significantly enhance light–matter interaction, thereby improving the overall photodetection performance. Moreover, through rational device engineering, such as heterostructure construction and electrode optimization, as well as material modulation strategies, including strain engineering and doping control, further improvements in responsivity, detectivity, and response speed can be achieved. Although waveguide-integrated 2D-material infrared photodetectors are still in the preliminary development phase, existing research has demonstrated their ability to rival, and in some cases even surpass, commercial photodetectors in several key aspects. Nonetheless, the field still faces numerous challenges, which include the creation and characterization of novel 2D materials, the controlled synthesis of high-quality films, low-loss transfer techniques, precise device engineering in complex heterostructures, and the expansion of waveguide material platforms along with optimized structural designs. Beyond optimizing individual figures of merit and managing their mutual trade-offs, future research should increasingly move toward application-oriented, system-level optimization of waveguide-integrated 2D-material infrared photodetectors. Ultimately, overcoming these challenges across the materials, device, and system levels, while ensuring stability, process uniformity, long-term reliability, and manufacturability, will be essential for translating waveguide-integrated 2D-material photodetectors from laboratory prototypes into commercially viable technologies.

Firstly, although a few representative 2D materials, such as graphene, have demonstrated promising potential in photodetector applications, their intrinsic limitations continue to hinder further improvements in device performance and large-scale deployment in practical scenarios. For instance, graphene’s zero bandgap results in a high dark current, making it less suitable for low-noise detection. BP, while offering high carrier mobility and a tunable bandgap, suffers from poor environmental stability due to its sensitivity to oxygen and moisture. TMDs such as MoS_2_ exhibit notable photoresponse, but their relatively low mobility limits the speed and efficiency of the devices. Therefore, the exploration of emerging 2D materials that simultaneously possess broadband photoresponse, environmental stability, and high carrier mobility has become a key strategy for advancing 2D-material-based photodetector technologies. Recently, emerging 2D materials with superior optoelectronic properties, like Te and Bi_2_O_2_Se, have been successfully incorporated into waveguide-integrated photodetector architectures and have demonstrated excellent infrared performance, highlighting their potential for on-chip integrated infrared detection^54,168^. Nevertheless, several other promising 2D materials remain underexplored. For example, 2D perovskites, known for their high carrier mobility, tunable bandgap, and outstanding photoelectric conversion efficiency, hold significant promise for infrared sensing, yet their integration with photonic waveguide platforms is still rare^283^. The underlying mechanisms and device performance metrics of such systems require further experimental investigation. Apart from the design and discovery of new materials, optimizing the performance of existing 2D materials remains an important direction. Recent advances in doping engineering^71^, strain tuning^43^, and twist-angle stacking^55^ have been extensively utilized to modulate the band structure and optoelectronic properties of 2D materials. Through precise control over their physical parameters, these approaches can enhance device performance, extend spectral response, and even enable novel functionalities.

Secondly, the practical implementation of 2D materials still faces significant challenges related to quality control during synthesis and transfer processes, which remain critical bottlenecks for their widespread adoption in photodetector applications. Currently, several fabrication approaches are utilized to prepare 2D materials, including mechanical exfoliation, liquid-phase exfoliation, and CVD, each with its benefits and drawbacks. Mechanical exfoliation, one of the earliest and most widely used methods, can yield high-crystalline-quality flakes with low defect densities. However, this method suffers from poor control over flake size and thickness, low throughput, and limited reproducibility, making it unsuitable for large-scale production^122^. Encouragingly, recent studies have reported mechanically exfoliated 2D materials with improved size uniformity and layer control, offering new possibilities for scaling up this technique^284^. Liquid-phase exfoliation, on the other hand, offers greater scalability and cost-efficiency and is well-suited for the mass production of 2D nanosheets. Nonetheless, it often results in materials with lower crystalline quality, higher defect densities, and variability in flake thickness. In addition, the use of organic solvents in this process can pose toxicity and environmental concerns, limiting its applicability in high-performance electronic and optoelectronic systems^285^. Among the various approaches, CVD is widely regarded as the most viable approach for achieving large-area, uniform synthesis of 2D materials. For example, graphene grown on copper substrates via CVD exhibits high carrier mobility over large areas, although the material quality is generally inferior to that of mechanically exfoliated samples^286^. Typically, CVD-grown films require wet transfer to the target photonic structures, a process that can introduce polymer residues, metallic contaminants, or microscopic cracks, ultimately degrading surface cleanliness and device performance^287^. Moreover, certain 2D materials, like BP, are highly sensitive to moisture and oxygen, making them susceptible to degradation during transfer or storage, which further compromises material quality and consistency. Consequently, achieving controlled, high-quality synthesis of 2D materials alongside low-loss, low-contamination, and high-fidelity transfer processes remains a critical technological challenge that must be addressed to fully unlock the potential of 2D materials in photonic integration.

Besides the remaining challenges associated with expanding the library of 2D materials, tuning their properties, and optimizing fabrication processes, waveguides, as essential structures for guiding optical modes and regulating light-matter interactions, also critically influence the performance of photodetectors through their material characteristics and structural design. Commonly employed waveguide materials, like silicon nitride and lithium niobate, face intrinsic bandgap limitations, hindering broadband photodetection capabilities. As a consequence, these waveguides often rely on heterogeneous integration approaches to incorporate additional photoactive materials. However, such integration methods introduce increased fabrication complexity, interfacial mismatches, and optical coupling losses between different device components^288^. Recently, the introduction of 2D-material waveguides has expanded the design space for simultaneously achieving optical transmission and efficient detection within the same material platform, thereby advancing waveguide-integrated photodetectors towards higher integration density and enhanced performance. For instance, combining graphene with metallic nanostructures enables efficient optical guiding in graphene waveguides, paving the way toward all-graphene-integrated PIC^289^. Additionally, recent achievements involving wafer-scale fabrication of MoS_2_ δ waveguides have significantly expanded the applicability of 2D materials in waveguide-integrated devices^290^. Atomically thin MoS_2_ behaves analogously to a quantum-mechanical delta potential, effectively confining optical fields within its bulk and surface regions and enabling millimeter-scale optical transmission from visible to near-infrared wavelengths. Moreover, owing to its near-zero structural dispersion, MoS_2_ waveguides inherently achieve impedance matching with free-space light, thus preserving waveform stability during optical coupling and propagation and circumventing the need for complex mode-conversion components, demonstrating substantial potential for on-chip interconnects and integrated photonic devices. Despite these promising attributes regarding optical manipulation and integration potential, systematic studies on utilizing 2D-material waveguides specifically for photodetection applications remain scarce. Hence, further expansion of waveguide material systems and achieving integrated functional coupling represent critical avenues for future breakthroughs in the field.

Apart from selecting suitable waveguide materials, the structural design of waveguides plays an equally critical role in developing high-performance photodetectors. Each type of waveguide structure exhibits distinct characteristics in terms of optical-field control, fabrication complexity, and suitability for different applications; thus, careful consideration and trade-offs are required based on specific use scenarios. For example, traditional rectangular and ridge waveguides feature mature fabrication processes and simplified manufacturing, making them highly suitable for large-scale production. Slot waveguides significantly enhance optical-field localization within their low-index gaps, greatly strengthening light-matter interactions, thereby making them especially advantageous for high-sensitivity applications such as gas sensing^209^. Photonic crystal waveguides utilize their unique slow-light effects to substantially reduce group velocities, prolonging interaction times between light and materials, and thus enhancing optical utilization. Combined with their inherent low transmission losses, photonic crystal waveguides are particularly well-suited for scenarios involving limited optical power budgets, such as long-distance optical communications^291^. Microring resonators leverage high-Q resonant modes to achieve strong optical confinement at specific wavelengths, demonstrating clear advantages in biosensing applications where signal recognition is achieved by monitoring resonant wavelength shifts^292^. Surface plasmon polariton waveguides, benefiting from their low group-velocity dispersion and inherently integrated metallic electrode configurations, enable ultra-broadband photodetection. Particularly when combined with high-mobility graphene, such devices can achieve bandwidths exceeding 100 GHz^212^. However, intrinsic metallic absorption losses remain a notable drawback, making surface plasmon polariton waveguides more appropriate for applications demanding extremely high bandwidth but moderate optical power, such as high-speed optical communication receivers or terahertz signal detection. Furthermore, analogous to the strategy of constructing heterostructures with different 2D materials to integrate diverse functionalities, researchers have explored hybrid waveguide structures that combine the strengths of different waveguide types. For instance, integrating surface plasmon polariton slot waveguides with silicon slot waveguides can simultaneously achieve high bandwidth and reduce metal-induced losses^73^. Nonetheless, the pursuit of specialized optical properties through unique waveguide designs often necessitates extremely high fabrication precision, which directly conflicts with the requirements of large-scale integration. Consequently, selecting an optimal waveguide structure that effectively balances performance metrics with fabrication complexity remains an urgent and challenging task, one that must be addressed through careful consideration of practical application requirements.

Whether by improving the properties of 2D materials or optimizing waveguide structures, the ultimate goal is to substantially improve key performance metrics of photodetectors, including responsivity, bandwidth, and dark current. As previously discussed, researchers have proposed numerous strategies to boost the capabilities of waveguide-integrated 2D-material infrared photodetectors, thereby improving their effectiveness in practical applications. Responsivity, a fundamental metric of photodetector performance, quantifies the efficiency with which optical signals are converted into electrical signals, directly determining the device’s capability for weak-light detection or operation in low-signal-to-noise-ratio environments. One effective strategy for enhancing responsivity involves employing 2D materials with inherently strong light absorption, such as TMDs. Additionally, combining 2D materials with zero-dimensional materials, like quantum dots or nanoparticles, can exploit quantum confinement effects or plasmonic resonance phenomena to significantly improve optical absorption, thus further elevating device responsivity^293^. Bandwidth, which defines the photodetector’s response speed, indicates the maximum frequency at which a detector can effectively respond to optical signals and is especially critical for high-speed signal processing and optical communication applications. The bandwidth of photodetectors strongly depends on carrier mobility within the chosen 2D material, carrier transit lengths, and the overall RC time constants of the device. To enhance bandwidth, researchers typically select high-mobility 2D materials such as graphene as active absorbing media. Additionally, shortening carrier transit paths effectively reduces the electron transit time within the device, thus improving response speed. For instance, vertical heterostructures can significantly enhance bandwidth by reducing source-drain distances^248^. Furthermore, achieving optimal Ohmic contacts helps minimize contact resistance and parasitic capacitances, facilitating rapid carrier injection or extraction, which further increases response speed^294^. Despite substantial progress in enhancing responsivity and bandwidth individually, simultaneously achieving both high responsivity and wide bandwidth within the same device remains challenging. Specifically, extended carrier lifetimes beneficial for responsivity enhancement typically result in reduced response speeds^208^. Thus, achieving an optimal balance between these two competing metrics remains a central challenge in the design of high-performance photodetectors.

Dark current is one of the key challenges limiting both the performance and practical deployment of 2D-material photodetectors, as it directly impacts the static power consumption, signal-to-noise ratio, and detection limit under low-light conditions. An increase in dark current not only raises the power dissipation but also introduces stronger noise, which severely degrades signal discrimination at low illumination levels and ultimately compromises overall device performance^149^. To address this issue, a variety of suppression strategies have been proposed from the perspectives of material selection, device architecture, and operating scheme. From the material side, choosing wider-bandgap 2D semiconductors, such as MoTe_2_, or employing gate control to tune the carrier density and Fermi level can effectively increase the potential barrier and suppress intrinsic leakage currents arising from thermally activated carriers and tunneling processes^258^. From the device-structure side, engineering heterojunctions has proven to be an effective approach, including 2D material-metal Schottky contacts^268^, 2D material-2D material heterostructures^265^, and hybrid 2D material-silicon configurations^38^. The built-in electric field formed at these interfaces enables efficient separation of photogenerated carriers without the need for large external bias, thereby maintaining strong photoresponse while substantially reducing the dark current. At the level of operation mechanism, photodetectors based on photothermal effects (such as PTE and BOL) offer an intrinsic advantage for dark-current suppression. In these devices, the output signal is mainly governed by temperature gradients induced by light or by conductivity variations with temperature, rather than by continuous carrier injection and drift in the dark^257^. As a result, they can operate at zero or low bias and significantly mitigate bias-dependent dark current and its associated noise. For waveguide-integrated 2D-material photodetectors, additional degrees of freedom arise from the use of resonant and dispersive photonic structures such as micro-ring resonators and photonic crystal waveguides. On the one hand, resonance enhancement in micro-rings and slow-light effects in photonic crystal waveguides can greatly strengthen the interaction between the guided optical mode and the 2D material, allowing sufficient photocurrent to be generated at lower input powers and reduced operating voltages, thus relaxing the need for high-bias operation. On the other hand, the markedly increased absorption per unit length makes it possible to shorten the effective interaction length or reduce the thickness of the 2D material while maintaining the desired responsivity, so that device miniaturization itself helps suppress dark current^49^. It is worth noting that several dark-current suppression strategies that have already proved effective in non-waveguide-integrated 2D photodetectors, such as intercalation engineering^295^ and defect passivation^296^, can in many cases be directly transferred to waveguide-integrated platforms and may provide additional room for performance enhancement.

Although performance optimization at the level of individual devices remains a fundamental aspect of research on waveguide-integrated 2D-material photodetectors, certain devices have already achieved remarkably competitive figures of merit, as discussed in Section 6. In parallel, the rapid development of on-chip systems for high-speed optical communication, optical computing, and artificial intelligence (AI) is placing increasingly stringent system-level requirements on photodetectors. As a result, the focus of the field is gradually shifting from the continuous improvement of isolated device metrics toward application-driven, system-level optimization. In this context, low-power operation, on-chip integration capability, and support for reconfigurable and multifunctional operation are emerging as particularly important directions. In on-chip optical computing systems, low-power operation is no longer only a desirable device metric. It has become a key constraint that directly determines the scalable computing capacity, the degree of parallelism, and the long-term operating stability of the system. For example, Tian et al. recently integrated a ferroelectric dielectric with graphene to realize a non-volatile graphene p-i-n homojunction on a waveguide platform^249^. In this device, the remanent polarization field of the ferroelectric layer stabilizes the carrier distribution without the need for a continuous gate bias or external driving voltage. Combined with the photothermoelectric response of graphene, this configuration enables zero-bias photodetection, substantially reducing power consumption and dark current while maintaining high responsivity and high-speed operation. This work provides a representative example of a device concept tailored for low-power on-chip integrated photodetection.

At the same time, in on-chip architectures driven by AI and optical computing, large-scale parallel processing imposes severe requirements on both the number of devices and the complexity of interconnects^297^. Therefore, high-density, system-level on-chip integration becomes a prerequisite for managing architectural complexity and improving the spatial and structural efficiency of the system. From an architectural perspective, waveguide-integrated 2D-material photodetectors are naturally suited to high-density on-chip integration. Light is confined within the waveguide, which allows multiple channels and degrees of freedom to be implemented within a compact footprint. This provides the physical basis for embedding photodetection functions into complex on-chip systems in a highly integrated manner. In high-speed optical communication, waveguide structures have already been used to implement key receiver-side functions such as polarization multiplexing^287^ and coherent detection^298^, with the associated photodetection processes integrated directly on chip. For instance, by incorporating 2D grating couplers or on-chip optical hybrid structures on a silicon waveguide platform, the polarization state or phase information of the incident light can be demultiplexed and demodulated on chip. Parallel arrays of integrated 2D-material photodetectors can then perform electrical readout. In this way, receiver chains that traditionally rely on multiple discrete optical components can be compressed onto a single chip, significantly reducing the system footprint and increasing the density of spatial integration. This demonstrates the potential of waveguide-integrated 2D-material photodetectors for realizing compact, highly integrated receiver architectures in high-speed optical communication. Building on this, multifunctionality at the level of a single device offers an additional route to further enhance system integration density. Owing to the strong tunability of the electrical and optical responses of 2D materials with respect to bias conditions, a single waveguide-integrated device can assume multiple functional roles under different operating states. Youngblood et al. showed that a graphene-based waveguide-integrated device can be switched between optical modulation and photodetection by electrostatic gating, allowing modulation and detection functions that are typically implemented by separate components in a communication link to be realized within a single device^299^. In another example, Bie *et al*. demonstrated that by adjusting the carrier injection scheme and bias polarity, a MoTe_2_ waveguide-integrated device can be reversibly switched between photodetection and electroluminescence operation, further highlighting the potential of 2D-material devices for functional reuse^300^. As the complexity of on-chip systems continues to grow, the co-integration of multiple functional devices on the same chip is also becoming an important trend. Recently, Huang et al. realized the on-chip co-integration of a 2D-material photodetector and an optical modulator on a silicon waveguide platform, enabling an optical signal to undergo modulation and subsequent detection within a single chip^301^. This work verifies the integration compatibility of different active components on a unified platform and illustrates the potential of such approaches for compact, functionally dense photonic systems.

Finally, in emerging on-chip applications related to AI and optical computing, computational tasks are often diverse and dynamic, with operators, weight configurations, and dataflow patterns changing frequently over time^302^. Under these conditions, photonic and optoelectronic systems that rely solely on static device configurations become increasingly limited in terms of flexibility and resource utilization, and reconfigurability is therefore emerging as an important direction for waveguide-integrated 2D-material photodetector systems. This trend is enabled by the strong sensitivity of the optoelectronic response of 2D materials to external electric fields, carrier injection schemes, and operating conditions. Recent studies have begun to exploit this tunability in application scenarios related to AI and optical computing, using waveguide-integrated 2D-material optoelectronic devices as functional elements in sensing front-ends for tasks such as target recognition, feature extraction, and multi-source information fusion^244^. These approaches emphasize performing part of the information processing at the point where data are generated or sensed, thereby reducing the computational and data-transport load on the electronic back end at the system level. Overall, a key challenge for the future will be to extend such reconfigurable behavior into scalable and programmable system-level implementations, while maintaining device stability and uniformity, so that waveguide-integrated 2D-material photodetectors can play a practical role in emerging applications in AI and optical computing.

As research on waveguide-integrated 2D-material photodetectors moves beyond materials and device-level performance optimization toward application-oriented and system-level design, it also becomes important to examine their status and challenges from the perspectives of engineering implementation and commercialization. By contrast, traditional photodetector technologies, such as Ge-based and III-V semiconductor detectors, have already been widely deployed in PICs and are supported by relatively mature industrial manufacturing ecosystems. These devices rely on standardized material growth and device fabrication processes, along with well-established engineering practices for performance stability, device uniformity, and reliability qualification. As a result, they serve as an important technological benchmark for photodetection functions in current integrated photonic systems. When viewed against these industrially established technologies, waveguide-integrated 2D-material photodetectors still face several key challenges on the path toward practical deployment, particularly in terms of large-scale integration, packaging reliability, manufacturing cost, and performance uniformity^303,304^. At the integration level, although recent studies have demonstrated wafer-scale or large-area integration of 2D optoelectronic devices, most of these advances focus on planar devices or free-space optical architectures. In comparison, waveguide-integrated 2D photodetectors impose more stringent requirements on material placement accuracy, evanescent-field coupling, and process co-design with the underlying photonic platform. As a consequence, current demonstrations remain largely at the laboratory level and are often limited to a small number of devices, and the capability for true large-scale integration still needs to be further developed. At the system-integration level, packaging and environmental stability remain nontrivial issues for 2D-material devices. Although a variety of encapsulation strategies can improve the environmental robustness of 2D materials to some extent, they still face limitations in long-term reliability, performance retention, and compatibility with large-scale manufacturing. For example, polymer encapsulation typically suffers from limited durability, whereas dielectric deposition or chemical functionalization may introduce performance degradation^305^. In addition, encapsulation layers can modify the optical mode profile or weaken electrical coupling, leading to trade-offs between device protection and optoelectronic performance and thereby constraining the practical use of such devices in photonic integrated chips. From a cost perspective, waveguide-integrated 2D-material photodetectors currently involve relatively high fabrication and integration complexity. In the absence of fully established, standardized, high-volume manufacturing processes, these devices cannot yet fully benefit from the economies of scale and process optimization available in mature industrial ecosystems. Compared with traditional photodetectors that are already widely adopted in PICs, the present manufacturing cost of 2D-material waveguide-integrated devices does not yet offer a clear advantage. On the performance side, waveguide-integrated 2D-material photodetectors still face challenges in stability and reproducibility. As material synthesis, transfer, and interface engineering have not yet been fully standardized, device characteristics remain highly sensitive to material quality and processing conditions. Defects and interface states can introduce additional noise and response fluctuations, which in turn impact device-to-device and batch-to-batch consistency. Moreover, the current landscape of 2D-material systems and device architectures is highly diverse: different materials, stacking configurations, and waveguide structures can lead to markedly different performance characteristics. While this diversity greatly expands the design space, it also causes optimization efforts to be fragmented and highlights the lack of broadly applicable design guidelines that simultaneously ensure robustness and balanced overall performance. Overall, waveguide-integrated 2D-material photodetectors are still predominantly at the laboratory research stage, and their commercialization is only beginning to emerge. Nevertheless, recent efforts by several companies and industrial groups to explore the engineering realization of 2D-material optoelectronic devices indicate a nascent transition from fundamental research toward practical applications^305^. As progress continues in material synthesis, process integration, and device reliability, 2D-material photodetectors are expected to retain substantial potential for future deployment in integrated photonic systems.

Overall, the majority of reported waveguide-integrated 2D-material infrared photodetectors are still at the prototype stage of laboratory demonstrations, and a clear gap remains relative to the level of industrial maturity achieved by commercial photodetectors. Nevertheless, recent progress has been considerable. Benefiting from the strong tunability of 2D materials and their compatibility with a variety of waveguide platforms, several representative devices already approach and in some cases match commercial detectors in key figures of merit such as bandwidth, responsivity, and noise under specific wavelength ranges and operating conditions. These advances are gradually pushing waveguide-integrated 2D devices beyond isolated single-device performance optimization toward system-level validation, for example, within high-speed optical communication links. Looking ahead, waveguide-integrated 2D-material photodetectors are well positioned to act as a valuable complement to established photodetection technologies rather than a direct replacement. If future work can achieve more reliable and reproducible progress in low-power operation, high-density on-chip integration, and reconfigurable functionality, while at the same time establishing basic standards for material preparation, process integration, and reliability evaluation, this class of devices may evolve from laboratory prototypes into practically deployable options and take on specific functional roles in next-generation infrared photonic integrated systems.
